# Sex differences in muscle protein expression and DNA methylation in response to exercise training

**DOI:** 10.1186/s13293-023-00539-2

**Published:** 2023-09-05

**Authors:** Shanie Landen, Macsue Jacques, Danielle Hiam, Javier Alvarez-Romero, Ralf B. Schittenhelm, Anup D. Shah, Cheng Huang, Joel R. Steele, Nicholas R. Harvey, Larisa M. Haupt, Lyn R. Griffiths, Kevin J. Ashton, Séverine Lamon, Sarah Voisin, Nir Eynon

**Affiliations:** 1 Institute for Health and Sport (iHeS), Victoria University, Melbourne, Australia; 2 Centre for Endocrinology and Metabolism, Hudson Institute of Medical Research, Melbourne, VIC Australia; 3 Institute for Physical Activity and Nutrition, School of Exercise and Nutrition Sciences, Deakin University, Geelong, Australia; 4 Monash Proteomics and Metabolomics Facility, Monash University, Melbourne, Australia; 5 Faculty of Health Sciences and Medicine, Bond University, Gold Coast, QLD 4226 Australia; 6 Centre for Genomics and Personalised Health, Genomics Research Centre, School of Biomedical Sciences, Queensland University of Technology (QUT), 60 Musk Ave., Kelvin Grove, QLD 4059 Australia; 7 Novo Nordisk Foundation Center for Basic Metabolic Research, Faculty of Health and Medical Sciences, University of Copenhagen, Copenhagen, Denmark; 8 Australian Regenerative Medicine Institute (ARMI), Monash University, Clayton, VIC 3800 Australia

**Keywords:** Sex differences, Skeletal muscle, Proteome, DNA methylation, Epigenetics, Exercise

## Abstract

**Background:**

Exercise training elicits changes in muscle physiology, epigenomics, transcriptomics, and proteomics, with males and females exhibiting differing physiological responses to exercise training. However, the molecular mechanisms contributing to the differing adaptations between the sexes are poorly understood.

**Methods:**

We performed a meta-analysis for sex differences in skeletal muscle DNA methylation following an endurance training intervention (Gene SMART cohort and E-MTAB-11282 cohort). We investigated for sex differences in the skeletal muscle proteome following an endurance training intervention (Gene SMART cohort). Lastly, we investigated whether the methylome and proteome are associated with baseline cardiorespiratory fitness (maximal oxygen consumption; *V*O_2_max) in a sex-specific manner.

**Results:**

Here, we investigated for the first time, DNA methylome and proteome sex differences in response to exercise training in human skeletal muscle (*n* = 78; 50 males, 28 females). We identified 92 DNA methylation sites (CpGs) associated with exercise training; however, no CpGs changed in a sex-dependent manner. In contrast, we identified 189 proteins that are differentially expressed between the sexes following training, with 82 proteins differentially expressed between the sexes at baseline. Proteins showing the most robust sex-specific response to exercise include SIRT3, MRPL41, and MBP. Irrespective of sex, cardiorespiratory fitness was associated with robust methylome changes (19,257 CpGs) and no proteomic changes. We did not observe sex differences in the association between cardiorespiratory fitness and the DNA methylome. Integrative multi-omic analysis identified sex-specific mitochondrial metabolism pathways associated with exercise responses. Lastly, exercise training and cardiorespiratory fitness shifted the DNA methylomes to be more similar between the sexes.

**Conclusions:**

We identified sex differences in protein expression changes, but not DNA methylation changes, following an endurance exercise training intervention; whereas we identified no sex differences in the DNA methylome or proteome response to lifelong training. Given the delicate interaction between sex and training as well as the limitations of the current study, more studies are required to elucidate whether there is a sex-specific training effect on the DNA methylome. We found that genes involved in mitochondrial metabolism pathways are differentially modulated between the sexes following endurance exercise training. These results shed light on sex differences in molecular adaptations to exercise training in skeletal muscle.

**Supplementary Information:**

The online version contains supplementary material available at 10.1186/s13293-023-00539-2.

## Highlights

- The skeletal muscle proteome displayed robust sex differences at baseline.
- The skeletal muscle proteome responded to 4 weeks of endurance training in a sex-specific manner.
- The skeletal muscle DNA methylome responded to 4 and 8 weeks of endurance training similarly between males and females.
- *V*O_2_max levels, an indicator of cardiorespiratory fitness and lifelong training, displayed a strong, universal signature on the muscle methylome, but not on the muscle proteome; both in a sex-invariable manner.
- Both endurance training and cardiorespiratory fitness shifted the DNA methylomes to be more similar between the sexes.

**Supplementary Information:**

The online version contains supplementary material available at 10.1186/s13293-023-00539-2.

## Abstract (plain language summary)

Exercise provides health benefits to every organ of the body, with specific genes and proteins changing in response to exercise. Males and females have distinct physiology which influences the body’s responds to exercise. However, it is largely unknown whether males and females respond differently to exercise on a molecular level. To function effectively, our muscles need specific proteins whose expression is regulated by a process called epigenetics. Epigenetics refers to modifications to our DNA that occur as a result of various environmental factors, such as exercise. We investigated sex differences in two aspects of molecular response to exercise: epigenetics and protein expression. We discovered that few weeks-long exercise programmes led to significant changes in protein expression but minimal changes in epigenetics. The proteins changed after exercise differed between the sexes and were involved in metabolism. This indicates that exercise has an immediate impact on muscle proteins in a sex-specific manner, but perhaps changes in epigenetics are slower. We then wondered whether a longer period of exercise would illicit sex-specific changes in epigenetics and protein expression. We showed that fitter individuals exhibited epigenetic differences compared to less fit individuals, while protein expression remained unchanged. Fit males and females showed similar epigenetic changes compared to their unfit counterparts. This suggests that lifelong training shifts the muscle epigenetic patterns in a similar manner in both males and females. Our findings emphasise the importance of lifelong fitness for stimulating epigenetic remodelling in muscle, as well as the importance of taking sex differences into consideration.

**Supplementary Information:**

The online version contains supplementary material available at 10.1186/s13293-023-00539-2.

## Background

Regular exercise is one of the most cost-effective and accessible ways to improve and maintain health, with evident benefits across many tissues and diseases [1]. Thus, there is much interest in understanding how physical activity promotes health at the molecular level [2]. Both a single acute bout of exercise and exercise training induce epigenetic changes in skeletal muscle, the most energy-demanding tissue during exercise [3]. Various modalities of exercise training modulate the skeletal muscle methylome [3], transcriptome [4], proteome [5], and subsequent physiology [6], ultimately promoting health benefits. Although males and females differ in their physiological response to exercise [7], much of our understanding of molecular adaptations to exercise is limited to studies where the majority of participants were male or sex was not accounted for [8, 9], despite sex modulating various biological processes. Therefore, elucidating sex-specific genes and pathways following exercise training is crucial for the comprehensive understanding of the molecular benefits of exercise.

Both the skeletal muscle methylome and proteome are responsive to exercise training [4, 5, 10–14], however sex differences have yet to be investigated. At baseline, the skeletal muscle transcriptome [15–19] and DNA methylome (from our lab, meta-analysis using Gene SMART cohort) [20] differ between the sexes, particularly across genes involved in metabolic processes; while baseline proteome sex differences have not been studied. The only investigation, to our knowledge, of skeletal muscle sex differences in -omic response to exercise training, is a recent transcriptome meta-analysis, which identified 247 genes differentially expressed in skeletal muscle following exercise training in males and females, with many of these genes involved in chromatin organisation [21]. However, it is unknown whether exercise triggers sex-specific responses at the epigenetic and/or proteomic level in skeletal muscle. It is also unknown whether the same genes and pathways display a sex-specific exercise response across different -omic layers, or whether the altered genes are distinct between the different -omic layers.

Varying exercise training modalities incur a multitude of systemic molecular and physiological health benefits, which vary depending on intensity, duration, and frequency. Specifically, moderate-intensity continuous endurance training (MICT) and high-intensity interval training (HIIT) both improve cardiovascular fitness, namely maximum oxygen consumption (*V*O_2_max) [22]. *V*O_2_max is a gold-standard, commonly used measurement to represent cardiorespiratory fitness, given its positive association with several metabolic health outcomes and negative association with cardiovascular disease risk [23]. Higher *V*O_2_max levels are representative of lifelong endurance training [24–26].

In the present study, we uncovered the epigenetic (DNA methylation) and proteomic signature of lifelong training [*V*O_2_max levels, representing cardiorespiratory fitness (CRF)] in skeletal muscle, and tested, for the first time, whether this signature was sex-specific. We also examined, for the first time, sex differences in genome-wide DNA methylation and protein expression changes following 4 and 8 weeks of endurance training. We investigated differences in muscle protein expression between males and females, regardless of training or CRF. We integrated the sex-specific epigenetic and proteomic responses to exercise training to uncover biological pathways differentially activated by endurance exercise training between males and females. Finally, we investigated whether exercise training and CRF shift the methylomes of the sexes to be more similar.

## Methods

### Datasets

The E-MTAB-11282 data were publically available and accessed from Array Express. The Gene SMART data were collected in our lab, have been used for other publications [20, 27, 28], and are publically available on GEO (GSE171140). For the Gene SMART study, the exercise training protocol, study design, and methods have been extensively outlined previously [24]. Brief descriptions regarding the Gene SMART study protocol are outlined below. The DNA methylome meta-analysis was conducted on E-MTAB-11282 and the Gene SMART datasets. The proteome analysis was conducted solely on the Gene SMART dataset.

### Muscle biopsy and blood sampling

Muscle biopsies were sampled from the *vastus lateralis* muscle after an overnight fast, using a suction‐modified Bergström needle, under local anaesthesia of the skin and fascia (1% Xylocaine). The muscle samples were cleaned of excess blood, fat, and connective tissue and then flash-frozen in liquid nitrogen and stored in – 80 ºC. Intravenous blood was drawn immediately after the biopsy.

### Study design and physiological measurements

An overview of the exercise protocol used in the Gene SMART (Skeletal Muscle Adaptive Response to Training) study has been previously published [25]. The training intervention consisted of 4 weeks of a control period, followed by 4 weeks of high-intensity interval training (HIIT) performed on a cycle ergometer. The sex comparison of physiological measurements (*V*O_2_max, PP, or LT), before and after the interventions, was analysed using a linear model of the form:

$${VO}_{2}\mathrm{max} \sim \mathrm{sex}*\mathrm{time}+\mathrm{age}.$$

### Controlling for diet

Participants were provided with individualised, pre-packaged meals for the 48 h prior to the resting muscle biopsies. The energy content of the provided meals was calculated using the Mifflin St-Jeor equation and each participant’s body mass, height and age [26]. The content of the diets were constructed based on the current National Health and Medical Research Council (NHMRC) guidelines. Participants were provided with a post-training and post-testing snack consisting of protein (0.3 g kg–1 BM) and carbohydrates (0.3 g kg–1 BM) [29]. Participants were asked to refrain from alcohol and caffeine during the dietary-control period, which is 48 h prior to each resting biopsy. Outside of the dietary-control period they were asked to continue with their normal exercise and dietary habits.

### Participants and control of confounders

Females with a regular menstrual cycle (26–35 days) [30] not taking hormonal contraceptives were recruited in order to obtain a homogenous cohort, as different contraceptives have different dosage, administration patterns, and different hormone combinations causing variability in metabolism and gene expression [31]. For consistency and to control for the potential effects of hormonal fluctuations during the female menstrual cycle, all biopsies were performed during the early follicular phase (days 1–7 of cycle).

Participants (total of six females and one male) served as their own controls as they underwent 4 weeks of a control period prior to starting the training, this was done in order to assess whether DNA methylation fluctuates with regular lifestyle (diet, sleep, exercise, etc.) in the absence of the exercise training intervention (Additional file 1: Fig. S1F).

### DNA extraction and methylation

Genomic DNA was extracted from the samples using the AllPrep DNA/RNA MiniKit (Qiagen, 80204) following the user manual guidelines. Global DNA methylation profiling was generated with the Infinium MethylationEPIC 850K BeadChip Kit (Queensland University of Technology and Diagenode, Austria). The first batch contained only males, were randomised for timepoint and age and were randomised across chips to minimise batch effects. The second batch contained males and females and samples were scrambled on the chips to ensure randomness when correcting for batch effect (i.e. old and young males and females across all time points included on each chip). The genome-wide DNA methylation pattern was analysed with the Infinium MethylationEPIC BeadChip array.

### Protein extraction and proteomics

Muscle tissue was lysed in 300 µl SDS solubilisation buffer (5% SDS, 50 mM TEAB, pH 7.55), heated at 95 °C for 10 min and then probe-sonicated before measuring the protein concentration using the BCA method. A total protein amount of 100 µg (suspended in 50 µl) was used for each sample for subsequent analyses. The lysed samples were denatured and alkylated by adding TCEP (Tris(2-carboxyethyl) phosphine hydrochloride) and CAA (2-chloroacetamide) to a final concentration of 10 mM and 40 mM, respectively, and the mixture incubated at 55 °C for 15 min. Sequencing grade trypsin was added at an enzyme-to-protein ratio of 1:50 and incubated overnight at 37 °C after the proteins were trapped using S-Trap mini columns (Profiti). Tryptic peptides were eluted from the columns using (i) 50 mM TEAB, (ii) 0.2% formic acid and (iii) 50% acetonitrile, 0.2% formic acid. The fractions were pooled, concentrated in a vacuum concentrator and reconstituted in 40 µl 200 mM HEPES, pH 8.5. Using a Pierce Quantitative Colorimetric Peptide Assay Kit (Thermo Scientific), equal peptide amounts of each sample were labelled with the TMTpro 16plex reagent set (Thermo Scientific) according to the manufacturer’s instructions and considering a labelling strategy to minimise channel leakage. Individual samples were pooled and high-pH RP-HPLC was used to fractionate each pool into 12 fractions, acquired individually by LC–MS/MS to maximise the number of peptide and protein identifications.

Using a Dionex UltiMate 3000 RSLCnano system equipped with a Dionex UltiMate 3000 RS autosampler, an Acclaim PepMap RSLC analytical column (75 µm × 50 cm, nanoViper, C18, 2 µm, 100 Å; Thermo Scientific) and an Acclaim PepMap 100 trap column (100 µm × 2 cm, nanoViper, C18, 5 µm, 100 Å; Thermo Scientific), the tryptic peptides were separated by increasing concentrations of 80% acetonitrile (ACN)/0.1% formic acid at a flow of 250 nl/min for 158 min and analysed with an Orbitrap Fusion Tribrid mass spectrometer (ThermoFisher Scientific). The instrument was operated in data-dependent acquisition mode to automatically switch between full scan ms1 (in Orbitrap), ms2 (in ion trap) and ms3 (in Orbitrap) acquisition. Each survey full scan (380–1580 *m*/*z*) was acquired with a resolution of 120,000, an AGC (automatic gain control) target of 50%, and a maximum injection time of 50 ms. Dynamic exclusion was set to 60 s after one occurrence. Cycle time was fixed at 2.5 s, the most intense multiply charged ions (*z* ≥ 2) were selected for ms2/ms3 analysis. Ms2 analysis used CID fragmentation (fixed collision energy mode, 30% CID Collision Energy) with a maximum injection time of 150 ms, a “rapid” scan rate and an AGC target of 40%. Following the acquisition of each MS2 spectrum, an ms3 spectrum was acquired from multiple ms2 fragment ions using Synchronous Precursor Selection. The ms3 scan was acquired in the Orbitrap after HCD collision with a resolution of 50,000 and a maximum injection time of 250 ms.

The raw data files were analysed with Proteome Discoverer (Thermo Scientific) to obtain quantitative ms3 reporter ion intensities.

### Proteomics bioinformatics analysis

Before normalisation, proteomic intensity data were filtered for high-confidence protein observations. In addition, contaminants, proteins only identified by a single peptide and proteins not identified/quantified consistently across the experiment were removed. The remaining missing values were imputed using the missing-not-at-random (MNAR) method, assuming the missingness was due to low expression for such proteins. Intensity was log transformed and normalised using the variance–stabilising–normalisation (VSN) method, which transforms the data in such a way that the variance remains nearly constant over the whole intensity spectrum (Additional file 1: Fig. S4). Both imputations and VSN were conducted by the *DEP* package [32]. Batch effects were corrected using internal referencing scaling (IRS) method [33] by the use of reference channels.

To identify differentially expressed proteins, we used linear models implemented in the *limma* package in R [34], using the participants’ ID as a blocking variable to account for the repeated measures design. Proteins showing a π-value < 0.005 were considered significant, which was calculated using the absolute value of the logFC and the FDR as described in Xiao et al. [35]. π-value is a mathematic combination of *p*-value and log2FC for better ranking of genes (calculated according to [35]), which was used for proteomics analysis.

### DNA methylation bioinformatics analysis

The pre-processing of DNA methylation data was performed according to the bioinformatics pipeline developed for the Bioconductor project [36]. Raw methylation data were pre-processed, filtered and normalised across samples. Probes that had a detection *p-v*alue of > 0.01, located on X and Y chromosomes or cross-hybridising, or related to a SNP frequent in European populations, were removed. It is important to note that the list of cross-hybridising probes was supplied manually [37] as the list supplied to the *ChAMP* package was outdated. Specifically, there are thousands of probes in the Illumina microarrays that cross-hybridise with the X-chromosome and may lead to false discovery of autosomal sex-associated DNA methylation [38]. The BMIQ algorithm was used to correct for the Infinium type I and type II probe bias. β-values were corrected for both batch and position in the batch using *ComBat* [39].

To identify DMPs, we used linear models as implemented in the *limma* package in R [34], using the participants’ ID as a blocking variable to account for the repeated measures design. All results were adjusted for multiple testing using the Benjamini and Hochberg correction [40] and all CpGs showing an FDR < 0.005 were considered significant for the association of DNA methylation with baseline fitness [41]. When no DMPs were detected at FDR < 0.005, we examined the histogram of *p*-values to evaluate whether results were truly negative or whether we were underpowered. CRF-associated DMRs were identified using the *DMRcate* package [42]. DMRs with Stouffer, Fisher, and harmonic mean of the individual component FDRs (HMFDR) statistics < 0.005 were deemed significant. Effect sizes are reported as mean differences in DNA methylation beta values (%).

We adjusted each EWAS for bias and inflation using the empirical null distribution as implemented in *bacon* [43]. Inflation and bias in EWAS are caused by unmeasured technical and biological confounding, such as population substructure, batch effects, and cellular heterogeneity [44]. The inflation factor is higher when the expected number of true associations is high; it is also greater for studies with higher statistical power [43]. The results were consistent with the inflation factors and biases reported in an EWAS in blood [43]. Results from the independent EWAS were combined using an inverse variance weighted meta-analysis with METAL [45]. We used METAL since it does not require all DNA methylation datasets to include every CpG site on the HumanMethylation arrays. For robustness, we only included CpGs present in both cohorts (639,759 CpGs). Both MICT [46] and HIIT [47] induce skeletal muscle DNA methylation and *V*O_2_max changes, therefore we were able to meta-analyse the Gene SMART and E-MTAB-11282 cohorts.

We integrated a comprehensive annotation of Illumina HumanMethylation arrays [48] with chromatin states from the Roadmap Epigenomics Project [49] and the latest GeneHancer information [50]. Baseline fitness-DMPs that were annotated to two differing chromatin states were removed for simplicity and because there were very few such DMPs. GSEA on Reactome and GO databases was performed on DMRs using the *goregion* (for GO) and *gsameth* (for Reactome) functions in the *missMethyl* R package [51, 52]. The linear models used are in Additional file 1: Fig. S1. Integration of the DNA methylome and proteome was performed using the *Mitch* R package utilising all genes in the analysis; for DNA methylation, gene statistics were averaged across CpGs annotated to the same gene.

For the analysis of the both proteome and DNA methylome, the linear models used were are of the form:

To assess for overall proteome/DNA methylome associations with training (denoted as “timepoint”) and CRF (denoted as “baseline*V*O_2_max”), irrespective of sex:

$$\mathrm{Proteome}/\mathrm{DNA \, methylome}\sim \mathrm{timepoint}+\mathrm{age}+\mathrm{sex}+\mathrm{baseline}V{\mathrm{O}}_{2}\mathrm{max}.$$

To assess for sex differences in proteome/DNA methylome associations with training:

$${\mathrm{Proteome}}/{\mathrm{DNA}} \, \mathrm{methylome}\sim \mathrm{timepoint}*\mathrm{sex}+\mathrm{age}+\mathrm{baseline}V{\mathrm{O}}_{2}\mathrm{max}.$$

To assess for sex differences in proteome/DNA methylome associations with CRF:

$${\mathrm{Proteome}}/{\mathrm{DNA}} \, \mathrm{methylome}\sim \mathrm{timepoint}+\mathrm{sex}*\mathrm{baseline}V{\mathrm{O}}_{2}\mathrm{max}+\mathrm{age}.$$

For DNA methylation analysis, batch was also included in the linear models for Gene SMART and lean/obese for E-MTAB-11282. Timepoint refers to before and after the training intervention. Age was included in the linear models given the known effect age on the methylome and proteome [53].

To assess whether CRF and training converge sex-biased DNA methylation sites, we ran a Pearson correlation between the first dimension of the principal component analysis (PCA) of sex-biased DMPs and CRF/training intervention. Furthermore, we compared the effects of sex vs sex*training and sex vs sex*CRF at these loci. Lastly, we removed the effects of the rest of the covariates by extracting the residuals of the linear models not containing the covariate of interest (training or CRF). This allowed to visualise whether training or CRF shifted the sex-biased methylome, when all other factors such as sex and age, were removed.

## Results

### Endurance training interventions of 4–8 weeks induce physiological, proteomic, and DNA methylomic changes, irrespective of sex

**Fig. 1:**
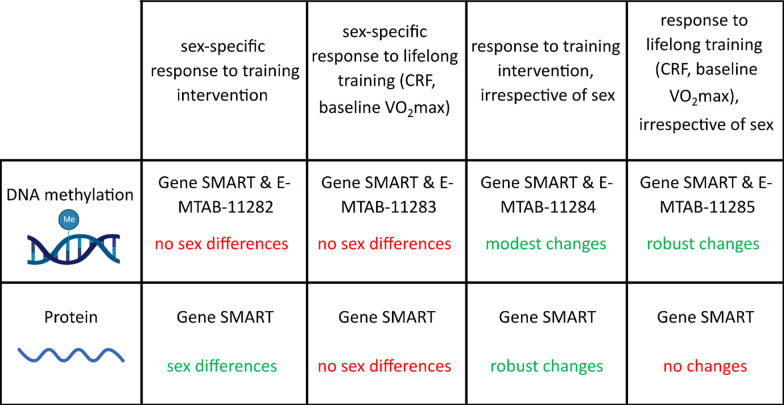
Analysis schematic. Summary of datasets used for each analysis performed in the study as well as the overall findings indicated in colour

We performed a meta-analysis of sex-specific DNA methylation response to exercise training utilising the Gene SMART cohort [24] and a recent dataset, E-MTAB-11282 [54]). The analysis of sex-specific proteome response to exercise training was performed solely on the Gene SMART cohort (Fig. 1). The Gene SMART study [24] consisted of 20 females and 45 males (aged 18–45 years) who completed 4 weeks of HIIT, and we collected measures of peak power output (PP), lactate threshold (LT), and *V*O_2_max before and after the exercise intervention. At baseline, males had higher aerobic fitness levels than females, in both absolute terms and relative to body weight (Table 1). Four weeks of HIIT elicited substantial improvements in PP and LT (*p*-value < 0.05, Table 1), but not *V*O_2_max (*p*-value = 0.109, Table 1), and there were no sex differences in the degree of response to 4 weeks of HIIT (*p*-value for interaction of time and sex > 0.05 for all fitness measurements, Table 1). A portion of the individuals completed a control period prior to the training intervention; no changes were observed in DNA methylation or physiological measurements following the control period. The E-MTAB-11282 cohort [54] consisted of five males and eight females (aged 21–54 years) who underwent 8 weeks of MICT. Participants from E-MTAB-11282 were healthy, sedentary, and either lean or obese. Similar to the Gene SMART cohort, males had higher *V*O_2_max levels than females, in both absolute terms and relative to body weight (*p* = 0.04, Table 1). *V*O_2_max did not increase significantly following the training intervention (*p* = 0.11; Table 1), with no sex differences in the degree of response (*p*-value for interaction between time and sex = 0.7; Table 1). Both males and females from the Gene SMART cohort had slightly higher cardiorespiratory fitness levels than those reported in the healthy general population for the corresponding age groups (males: 48.6 in Gene SMART vs 35–45 ml/min/kg in the general population; females: 44.1 in Gene SMART vs 30–40 for ml/min/kg in the general population) [55]. In contrast, both males and females from E-MTAB-11282 had lower cardiorespiratory levels than those reported in the healthy general population for the corresponding age groups (E-MTAB-11282 males: 23.1 ml/min/kg; females 20.3 ml/min/kg), although this cohort was made up of lean and obese individuals who were otherwise healthy.

**Table 1:** Aerobic fitness parameters of males and females from the Gene SMART and E-MTAB-11282 cohorts before and after 4 weeks of high-intensity interval training (HIIT) and 8 weeks of moderate-intensity continuous training (MICT), respectively

	Gene SMART										
	Males			Females			Males + females (combined)			Sex comparison	
	PRE	4WP	*p*-value^a^	PRE	4WP	*p*-value^a^	PRE	4WP	*p*-value (time)^a^	*p*-value (sex)^b^	*p*-value (time:sex)^b^
*V*O_2_max	48.63159	48.86683	0.6802	44.05717	45.51434	0.006209	47.18302	47.80521	0.1429	0.01584	0.7056
Lactate threshold	2.657964	2.862906	1.58 × 10^−8^	2.269863	2.462794	0.000183	2.53956	2.740838	6.93 × 10^−12^	0.009426	0.9808
Peak power	3.815436	4.035649	1.39 × 10^−8^	3.289923	3.492371	1.44 × 10^−5^	3.654392	3.869161	1.14 × 10^−12^	0.001412	0.9454

	E-MTAB-11282										
	Males			Females			Males + females (combined)			Sex comparison	
	PRE	4WP	*p*-value^a^	PRE	4WP	*p*-value^a^	PRE	4WP	*p*-value (time)^a^	p-value (sex)^b^	*p*-value (time:sex)^b^
*V*O_2_max	23.1	27	0.261	20.3	23.2	0.047	21.4	24.7	0.109	0.042	0.731

^a^Analyzed using paired *t* test

^b^Analyzed using ANOVA

Utilising data from solely the Gene SMART study, we investigated potential sex differences in the muscle proteome following an endurance training intervention and lifelong training, represented by cardiorespiratory fitness (CRF, baseline *V*O_2_max). Utilising data from the Gene SMART study and E-MTAB-11282 [54], we performed a meta-analysis of sex differences in DNA methylation following an endurance training intervention and lifelong training (CRF, baseline *V*O_2_max). Both MICT [46] and HIIT [47] induce skeletal muscle DNA methylation and *V*O_2_max changes, therefore we were able to meta-analyse the Gene SMART and E-MTAB-11282 cohorts.

**Fig. 2:**
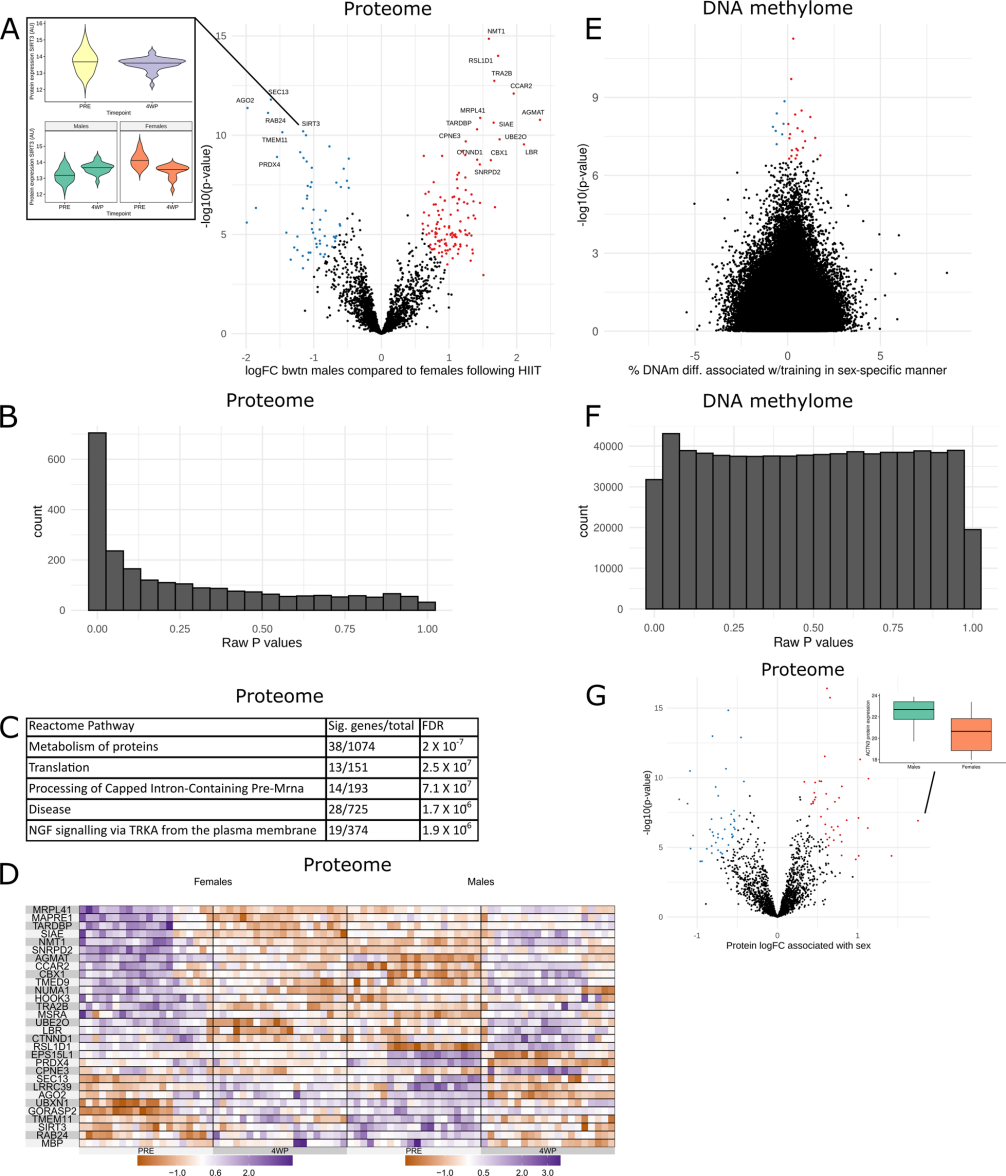
Sex-specific proteome and DNA methylome responses to an endurance training intervention and sex-specific proteome at baseline. **A** Volcano plot showing the sex-specific effect of 4 weeks of HIIT on the 2317 tested proteins. *X*-axis is the log2 of fold change in males compared to females; *y*-axis is the − log10 of unadjusted *p*-value. The 195 significant proteins (π-value < 0.005; computed according to [35]) are displayed in colours, with red dots denoting proteins with a positive coefficient in males compared to females following HIIT, and blue dots denoting those with a negative coefficient. Top 20 significant proteins are labelled. Violin plots to left indicates expression levels (arbitrary units) of SIRT3 in data pooled for sexes (upper) and stratified by sex (lower) before and after HIIT; horizontal line in violin indicates the median. **B** Histogram of raw p-values for the sex-specific effect of training on the proteome. **E **Volcano plot showing the sex-specific effect of 4 and 8 weeks of training on the 641,715 tested CpGs. The nine DMPs at a false discovery rate (FDR) < 0.005 are displayed in colorus, with red dots denoting DMPs with a larger coefficient in males, and blue dots denoting DMPs with a lower coefficient in males. **C** Top five Reactome pathways from gene set enrichment analysis of the differentially expressed proteins between the sexes following HIIT. **D** Heatmap of scaled protein expression values for the top 30 significant proteins (π-value < 0.005) that change in a sex-specific manner following HIIT. Rows are proteins ordered hierarchically according to the clustering in the female cohort; columns are female (left) and male (right) participants ordered according to hierarchical clustering within each time point. Purple denotes higher expression and orange denotes lower expression. Colour scales are separate for each sex. **F** Histogram of raw p-values for the sex-specific effect of training on the methylome. **G** Volcano plot showing the effect of sex on baseline levels 2317 tested proteins. *X*-axis is the log2 of fold change in males compared to females; *y*-axis is the − log10 of unadjusted *p*-value. The 82 significant proteins (π-value < 0.005; computed according to [35]) are displayed in colours, with red dots denoting proteins higher expression in males compared to females at baseline, and blue dots denoting those lower in males. Boxplot on right denote protein ACTN3 with higher expression in males compared with females at baseline

We identified 63 proteins differentially expressed following HIIT, irrespective of sex (π-value < 0.005; Additional file 2, see “Methods” for π-value). π-value is a mathematic combination of *p*-value and effect size that improves gene ranking [35], which was used for proteomics analysis. These proteins were mostly upregulated (77%) following the training intervention and were enriched for pathways such as metabolism of proteins and MTORC1-mediated signalling (π-value < 0.005; Additional file 2). We identified 82 proteins differentially expressed between the sexes at baseline, irrespective of training and CRF (included in model as covariates; π-value < 0.005; Additional file 2; Fig. 2G). 49% of the sex-specific proteins at baseline were more abundant in males than females. Sex-specific proteins were enriched for pathways such as metabolism of proteins and mRNA splicing (π-value < 0.005, Additional file 2; Additional file 1: Fig. S2E).

**Fig. 3:**
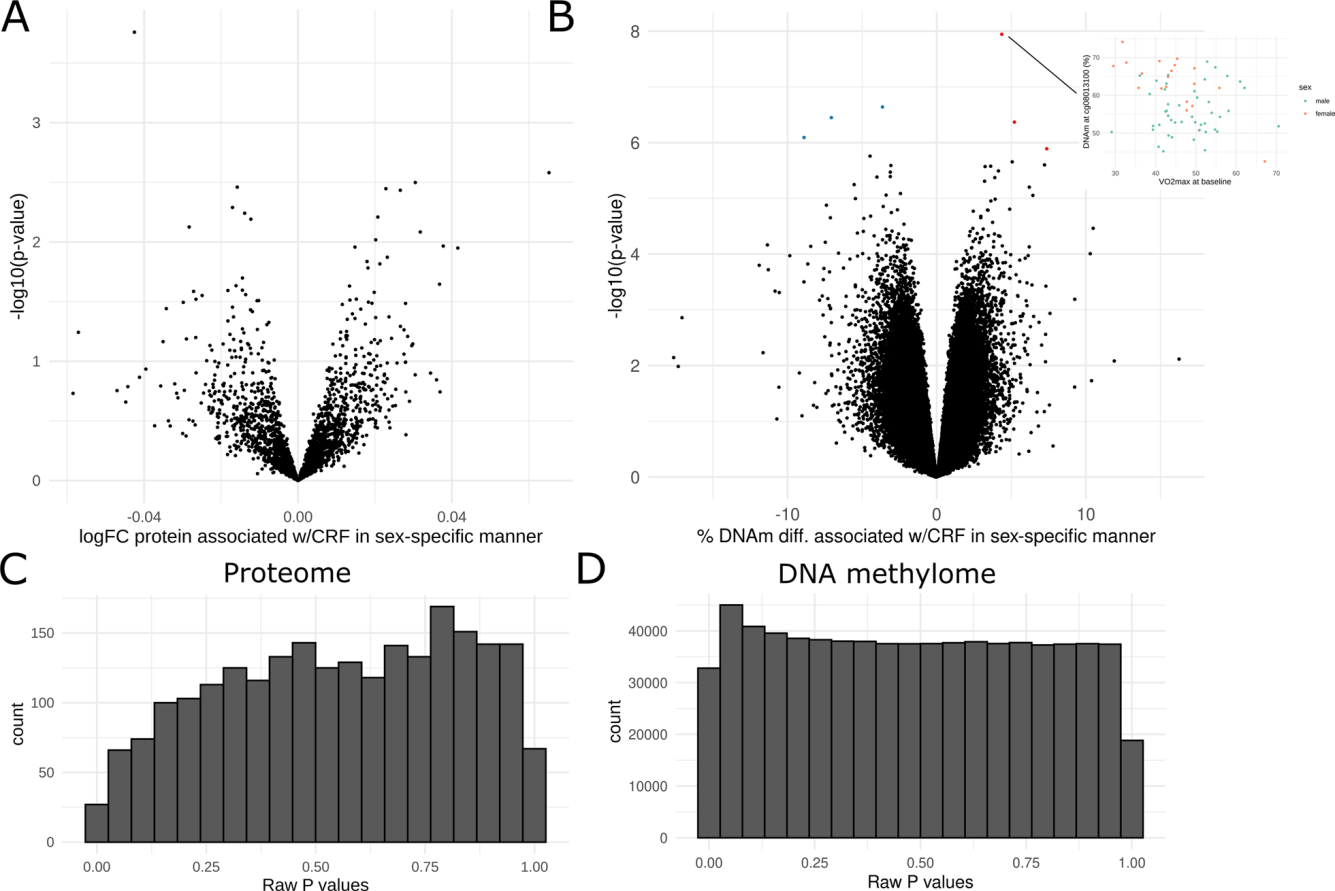
The effect of sex on the proteome and DNA methylome associations with cardiorespiratory fitness. **A** Volcano plot showing the effect of sex on cardiorespiratory fitness (CRF; baseline *V*O_2_max) on the 2317 tested proteins. *X*-axis is the log2 of fold change in males compared to females; *y*-axis is the –log10 of unadjusted *p*-value. No significant proteins were identified (π-value < 0.005; computed according to [35]). **B** Volcano plot showing the effect of sex on CRF on the 641,715 tested CpGs. The six differentially methylated positions (DMPs) at a false discovery rate (FDR) < 0.005 are displayed in colours, with red dots denoting DMPs with a larger coefficient in males, and blue dots denoting DMPs with a lower coefficient in males. Dotplot to the right displays DNAm beta-values of the denoted DMP in Gene SMART cohort individuals corresponding to CRF, coloured by sex. Plotted beta values, residuals from batch produced similar results. **C** Histogram of raw *p*-values for the sex-specific effect on the proteome. **D** Histogram of raw *p*-values for the sex-specific effect on the methylome

We identified 92 CpGs whose methylation changed after endurance exercise training (irrespective of sex) at FDR < 0.005 (Fig. 3E; Additional file 2). Thirty-eight % of the differentially methylated positions (DMPs) increased in methylation following the intervention (Additional file 2). These genes were not overrepresented in Gene Ontology (GO) terms or Reactome pathways (FDR < 0.005) (Additional file 2).

### The proteome, but not the DNA methylome, respond to a short-term endurance training intervention in a sex-specific manner

We found no sex-specific DNA methylation changes after the training intervention at FDR < 0.005 in the meta-analysis of the Gene SMART and E-MTAB-11282 cohorts (Additional file 2). A global examination of all the statistical tests performed did not reveal an inflation of near zero p-values, suggesting that males and females do not differ in their epigenetic response to 4 and 8 weeks of endurance training (Additional file 1: Fig. S1A).

In contrast, training triggered marked sex-specific proteome changes in muscle. We identified 189 proteins that showed different degrees of response between the sexes (π-value < 0.005 for interaction between sex and training; Fig. 3A). Approximately two-thirds (67%) of the proteins showing a sex-specific response displayed a larger increase in males compared with females. Proteins showing the most robust (i.e. largest π-value) sex-specific response to HIIT include SIRT3, MRPL41, and MBP (Fig. 3B). For example, SIRT3 showed an overall small and insignificant 0.13 logFC following HIIT when males and females are pooled together (π value = 0.85), but analysing the data for a sex-specific response revealed that HIIT-induced changes in SIRT3 levels were ~ 3 times greater in males compared with females (π value = 7.5 × 10^−10^; 1.2 logFC in males compared with females; Fig. 3A). Proteins showing a sex-specific training response were enriched for Reactome pathways involved in protein metabolism and mRNA splicing/binding (Fig. 3C; Additional file 2). To understand whether this sex-specific response may be due to sex-specific differences in fibre type proportions (as sex differences in fibre type proportions have been previously identified in the Gene SMART cohort [20] and other cohorts [56]), we overlapped the proteins we identified as showing a sex-specific response to training with those reported to differ between type I and type II muscle fibres in response to training [5] (134 fibre-specific proteins). For example, if the sex-specific protein changes were driven by fibre-specific responses to exercise, we would expect fibre-specific proteins to be overrepresented among the 189 proteins we identified as showing a sex-specific response. There were only eight fibre-specific proteins among the 189 proteins, which was a non-significant overlap (hypergeometric test *p*-value = 0.26).

### Cardiorespiratory fitness has a strong, universal signature on the muscle methylome, but not on the muscle proteome

Despite the lack of convincing evidence for a sex-specific DNA methylation response to short-term training, it is possible that 4/8 weeks of exercise training may have been too short to reliably detect sex-specific epigenetic responses to exercise training. Therefore, we then assessed whether lifelong training was associated with a sex-specific epigenetic signature in skeletal muscle. Baseline levels of cardiorespiratory fitness, or *V*O_2_max, reflect a combination of lifelong training, genetics, and other variables (age, sex, etc.). The signature of CRF on the muscle methylome was similar in males and females (FDR < 0.005 for sex-by-fitness interaction; Additional file 2). A global examination of all the statistical tests performed genome-wide did not reveal an inflation of near zero *p*-values, supporting results from the training intervention indicating a lack of sex-specific epigenetic response to exercise training (Additional file 1: Fig. S1D).

Irrespective of sex, we observed a strong signature of CRF, represented by baseline *V*O_2_max, on the skeletal muscle methylome (Additional file 2). We found 19,257 DMPs associated with CRF (FDR < 0.005), with moderate-to-large effect sizes (− 0.8% to 0.5% DNA methylation difference per unit of *V*O_2_max (Fig. 3A; Additional file 1: Fig. S1E, S2B). Given that *V*O_2_max ranged from 22 to 65 (Gene SMART) and 16.0–34.6 (EMTAB-11282) min/L/kg, a CRF-associated CpG differed up to ~ 30% between the fittest and least fit individuals of the cohorts. 31% of DMPs increased in methylation in fitter individuals and were underrepresented in CpG islands and active promoters while overrepresented in enhancers and in regions flanking active promoters (Fig. 3B, C; Additional file 1: Fig. S3) (χ^2^ *p*-value < 2.2e−16). DMPs clustered into 1948 differentially methylated regions (DMRs) located in 1873 unique genes (Differentially Methylated Genes, DMGs) (Additional file 2)*.* DMPs were involved in Reactome pathway muscle contraction, (FDR = 0.03; Additional file 2), as well as several skeletal muscle-related GO terms such as actin filament-based process, myofibril, and muscle contraction (Additional file 2). There was a significant overlap between the differentially methylated genes we identified, and genes previously reported to display DNA methylation and transcriptional changes after 3 months of leg-extensor training [4] (hypergeometric test *p*-value = 4.0 × 10^–14^), such as *SMAD3*.

We did not observe a distinct signature of CRF on the skeletal muscle proteome, with no proteins associated with CRF (π-value < 0.005; Fig. 3A). Furthermore, we found no proteins to be differentially associated with CRF between the sexes (Additional file 1: Fig. S1J).

### Integration of sex differences in the DNA methylome and proteome associated with training and cardiorespiratory fitness

**Fig. 4:**
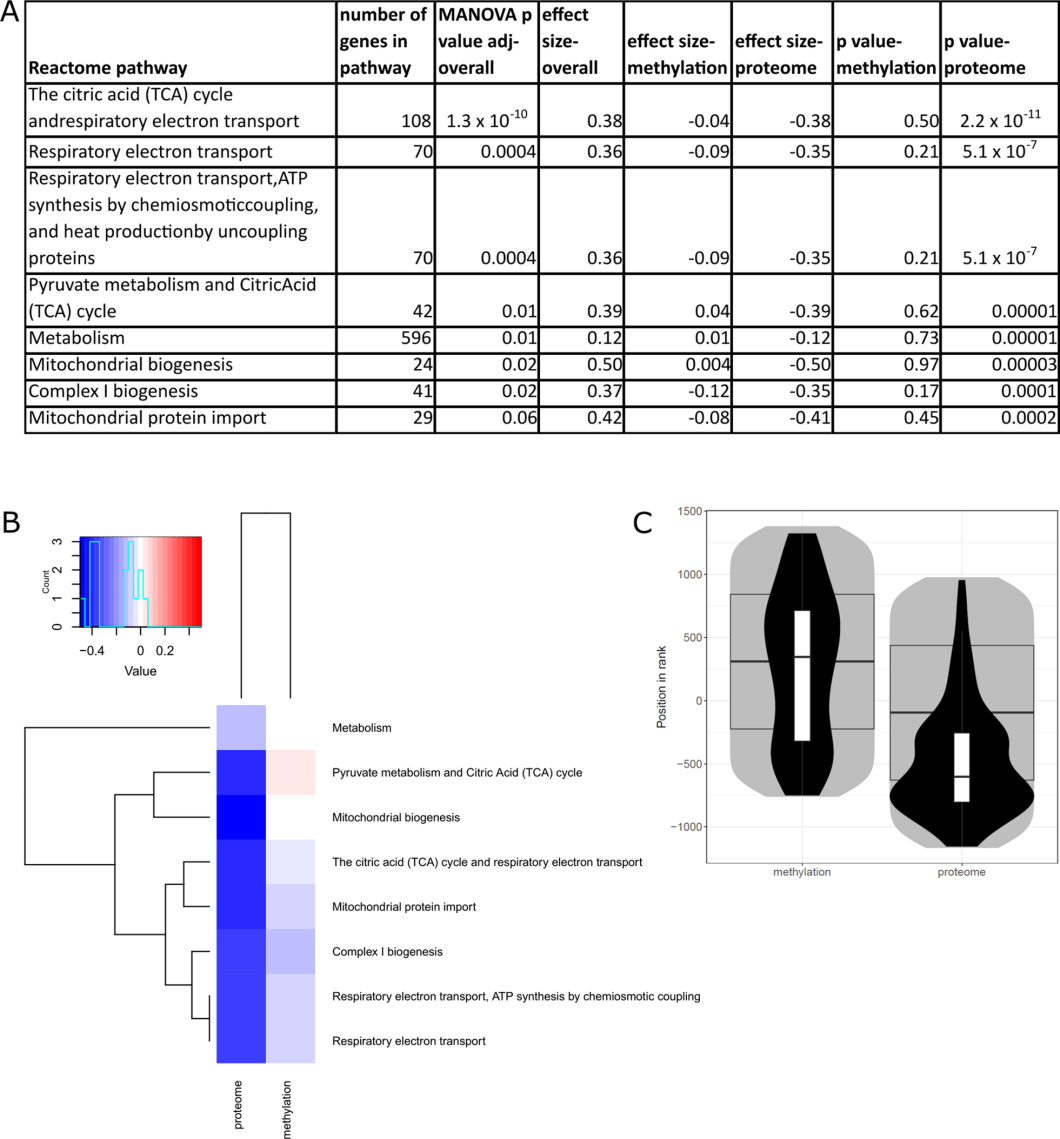
Integration of sex-specific proteome and DNA methylome responses to training. **A** Summary statistics of the Reactome pathways enriched among the sex-specific response to training in the integration of the proteome and DNA methylome (FDR < 0.005). **B** Heatmap of the pathways in **A**; colour key denotes direction of effect size. **C** The distribution of genes in the most significant pathway, TCA cycle and respiratory electron transport pathway; grey areas denote the distribution of ranks of all detected genes, with median and quartiles depicted by the wide boxplot. Distribution of Reactome pathway members is shown by the black violin, with median and interquartile ranges given by the narrow boxplot

**Fig. 5:**
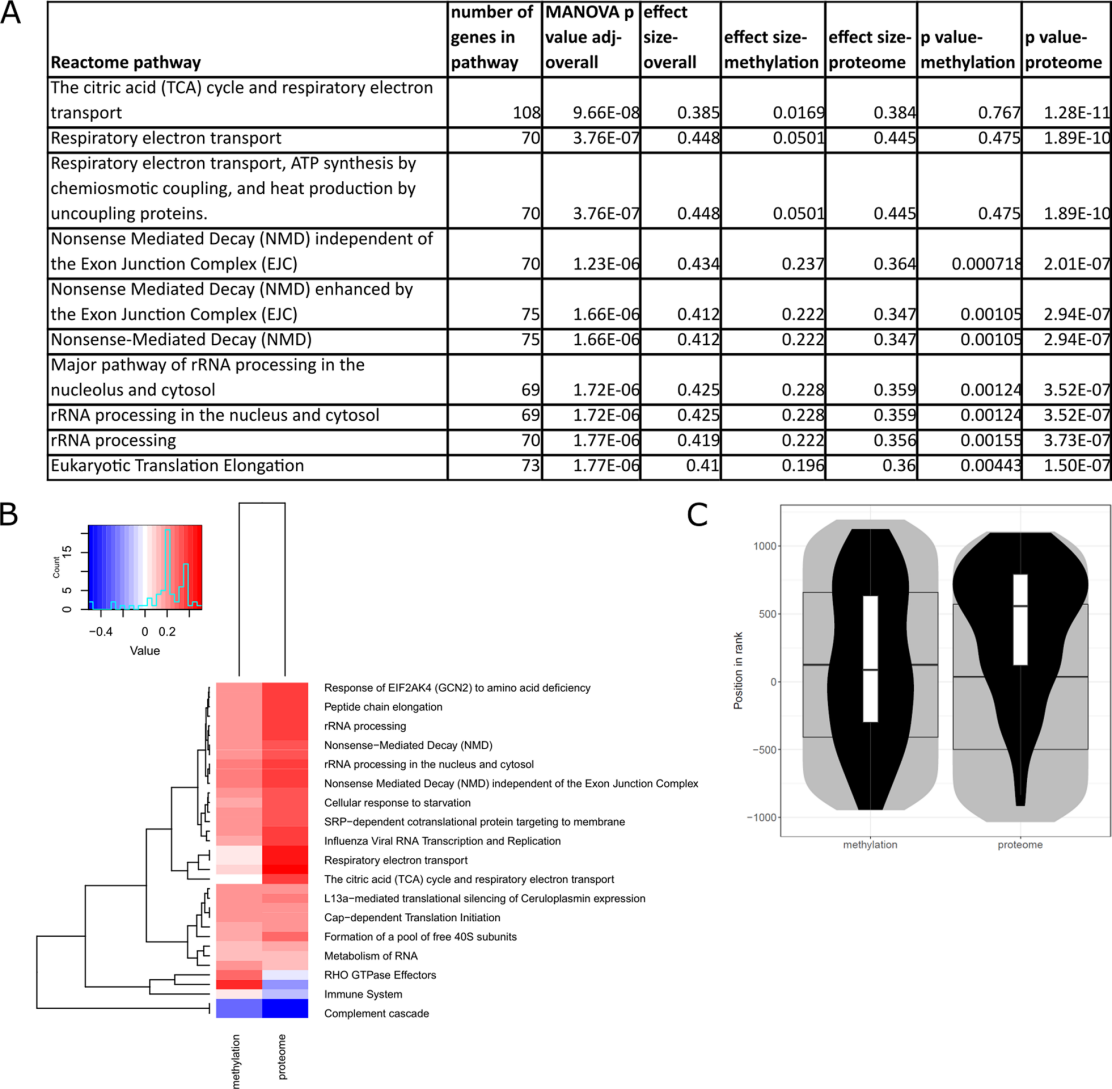
Integration of sex-specific proteome and DNA methylome association with cardiorespiratory fitness. **A** Summary statistics of the Reactome pathways enriched among the sex-specific association with cardiorespiratory fitness (CRF) in the integration of the proteome and DNA methylome (FDR < 0.005). **B** Heatmap of the pathways in **A**; colour key denotes direction of effect size. **C** The distribution of genes in the most significant pathway, TCA cycle and respiratory electron transport pathway; grey areas denote the distribution of ranks of all detected genes, with median and quartiles depicted by the wide boxplot. Distribution of Reactome pathway members is shown by the black violin, with median and interquartile ranges given by the narrow boxplot

We integrated the summary statistics for sex-specific DNA methylation (meta-analysis of Gene SMART study and E-MTAB-11282) with summary statistics for sex-specific proteome (Gene SMART) both following training and in association with CRF (Figs. 4, 5). The integration ranks the genes in each -omic layer and considers all genes tested, allowing an integration despite lack of significant findings, and outputs enriched pathways. Reactome pathways TCA cycle, respiratory electron transport, pyruvate metabolism, metabolism, and mitochondrial biogenesis were enriched among the integration of the sex-specific proteome and methylome following training (MANOVA *p* value < 0.005; Fig. 4A, B; Additional file 3). The most significant pathway in the integration of sex-specific training response of the methylome and proteome was TCA cycle and respiratory electron transport (MANOVA *p* value 1.1 × 10^–7^; effect size = 0.4; Fig. 4C), with the top three genes being NDUFS6, NDUFS2, and TACO1.

We next performed the same -omics integration for associations with CRF. Reactome pathways such as the TCA cycle, respiratory electron transport, nonsense mediated decay, and rRNA processing were enriched among the integration of sex-specific association with CRF of the proteome and methylome (MANOVA *p* value < 0.005, Fig. 5A, B; Additional file 4). The most significant pathway in the integration of sex-specific association with CRF in methylome and proteome was TCA cycle and respiratory electron transport (MANOVA p value 9.7 × 10^–8^; effect size = 0.4; Fig. 5C), with the top three genes being NDUFS7, NDUFC2, and NDUFS4.

### Training and cardiorespiratory fitness converge the sex-biased DNA methylomes of males and females

**Fig. 6:**
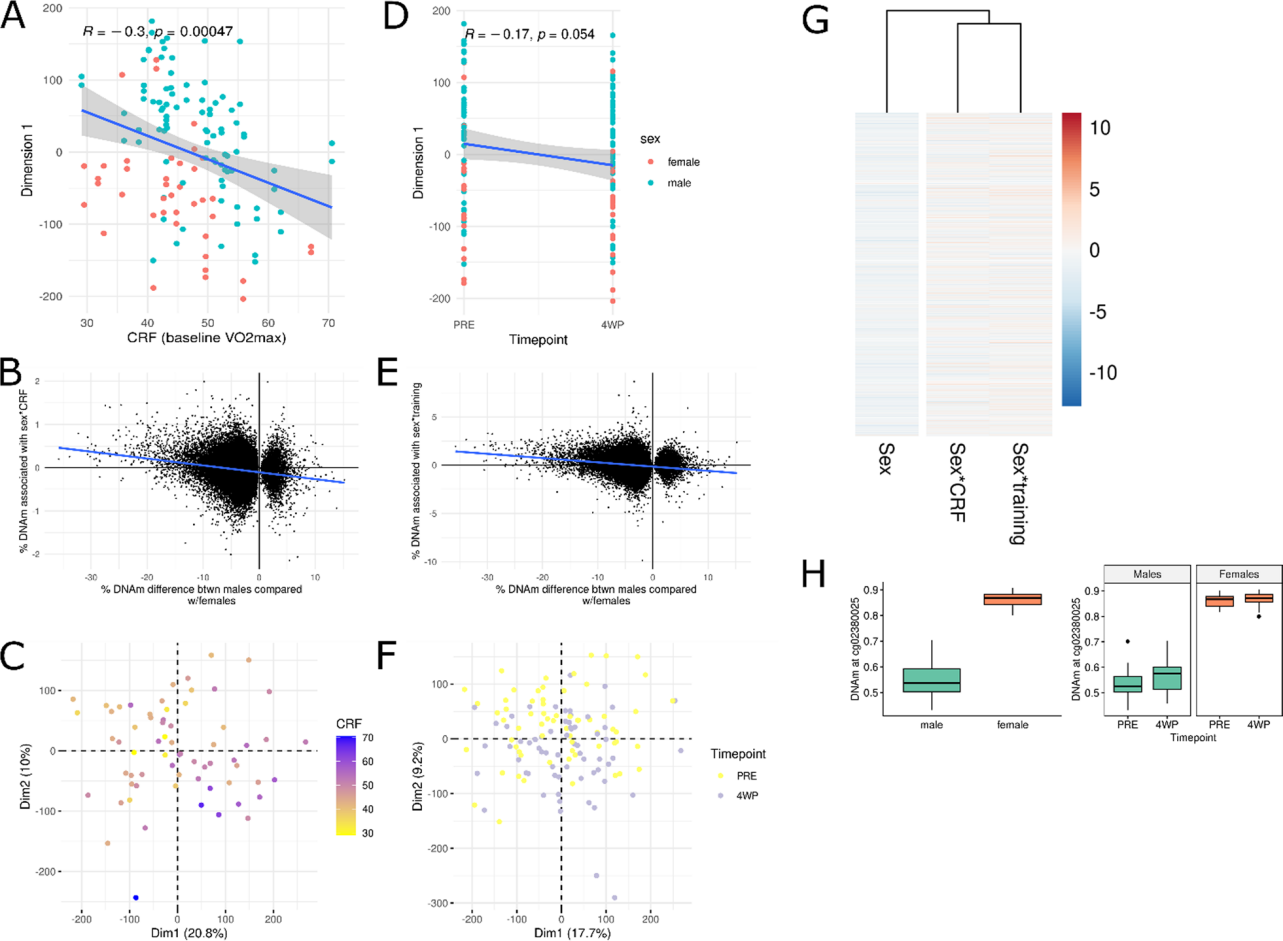
Effect of training and cardiorespiratory fitness (CRF) on sex-biased DNA methylation sites. Pearson correlation between the first dimension of the principal component analysis (PCA) of the 56,798 sex-differentially methylated positions (DMPs, at baseline, from [20]) and **A** cardiorespiratory fitness (baseline *V*O_2_max) or **D** timepoint (of training intervention). “PRE” denotes before the training intervention and “4WP” denotes following the training intervention. Data represented are from the Gene SMART study. Correlation between the effect sizes of sex versus **B** sex*CRF and versus **E** sex*training for the 56,798 sex-DMPs. Principal component analysis of residuals of model for **C** CRF and **F** training at the 56,798 sex-DMPs. Each dot is an individual and colours denote *V*O_2_max (mL/min/kg; **C**) and timepoint (**F**). **G** Heatmap of the effect sizes of the sex-DMPs for sex, sex*CRF, and sex*training. Each row is a sex-DMP; red denotes a positive effect size and blue denotes a negative effect size (scaled). **H** Boxplot of a CpG site (cg02380025) which has opposite effect sizes for the effect of sex versus sex*training. The left boxplot represents the effect of sex and the right boxplot represents the effect of sex*training. *Y*-axis is the DNAm beta values

Given that the muscle methylomes display profound sex bias at rest (56,798 DMPs [20]) and that exercise training and CRF modulate the methylome similarly in males and females, we next wondered whether methylomes of males and females converge or stay equally dissimilar with exercise training. In other words, whether trained males and trained females are more similar than untrained males and untrained females, on an epigenetic level. To address this, we investigated the effect of training and CRF on sex-biased DMPs. We found that the first dimension of the principal component analysis (PCA) of sex-biased DMPs was negatively correlated with CRF (*R* = − 0.3, *p* = 0.0005; Fig. 6A) and slightly negatively correlated with the training intervention (*R* = − 0.17, *p* = 0.05; Fig. 6D). To further corroborate that training and CRF have negative (i.e. opposite) effects on sex-biased DNA methylation loci, which would suggest that training and CRF have the opposite effect of sex at these loci and that sex methylomes converge with fitness, we compared the effects of sex vs sex*training and sex vs sex*CRF at these loci. We found that both sex*CRF and sex*training effect sizes were negatively correlated with sex effect sizes at sex-biased DNA methylation loci (Fig. 6B, E, G). Lastly, to visualise whether training and CRF modulate the sex-biased methylome, we compared the residuals of the linear models not containing the covariate of interest (training or CRF). This allowed to visualise whether training or CRF shifted the sex-biased methylome, when all other factors such as sex and age, were removed. Residuals of sex-biased DMPs clustered according to CRF and timepoint on both of the first dimensions (Fig. 6C, F), suggesting that CRF and timepoint contribute to the variance of sex-DMPs.

## Discussion

We investigated whether genome-wide DNA methylation and protein-wide expression in skeletal muscle have sex-specific responses to endurance training and whether male and female muscle displays distinct DNA methylation or protein signatures of baseline CRF. Finally, we investigated whether training or CRF converge the sex-biased DNA methylomes of males and females. The endurance training interventions induced modest changes in the DNA methylome, and we detected no sex-specific response; whereas, a training intervention induced more robust changes in the proteome as well as a sex-specific response. CRF at baseline was associated with widespread DNA methylation changes and minimal protein changes in muscle, but these were independent of sex. Proteins associated with a sex-specific response to training were involved in biological processes known to display sex differences among other gene regulatory levels, such as protein metabolism and mRNA splicing/binding pathways. The integrations of the DNA methylome and proteome both following training and in association with cardiorespiratory revealed sex-specific regulation of TCA and electron transport pathways. Finally, CRF and training converged the methylomes of males and females. Altogether, this proposes that long-term training, as is represented by CRF, induces lasting effects in the methylome, but that perhaps the proteome is more transient in nature. Furthermore, short-term training (4 or 8 weeks of endurance) may elicit a greater acute proteomic response than the methylome, given its more transient nature. These data suggest that while the acute response to training is more pronounced in the proteome and is sex-specific, the long-term effects are better portrayed by the methylome and converge the methylomes of the sexes. Overall, our findings imply that long-term training shifts the molecular profiles of males and females similarly.

### DNA methylome and training

Four and 8 weeks might be relatively short training interventions to elicit changes in DNA methylation, in particular for Gene SMART’s recreationally active participants, nonetheless, we detected small overall changes in the muscle methylome after training, most of which were hypomethylated. This was corroborated by our observation that fitter individuals displayed hypomethylation compared with less fit individuals. A handful of studies have reported DNA methylation changes in skeletal muscle after short-term (< 6 months) resistance or endurance exercise training [4, 46, 57]. Conversely to our results, existing studies reported equal global fractions of hypo- and hyper-methylation following training [4, 46, 57]. Two of these studies contained only males, and in the only study containing females [4], sex was confounded with batch and therefore could not be statistically taken into account. Batch effects in the Illumina arrays can significantly confound results and it is often not possible to resolve signal when batches are confounded with variables of interest [58], therefore samples should be strategically positioned in the array. Thus, the disparity in fractions of global hyper- and hypo-methylation between our study and those in the literature may be due to the inclusion of both sexes and the subsequent statistical adjustment. One study failed to detect changes in the muscle methylome after HIIT/resistance/combined training [47], but their analysis was restricted to promoters and to DNA methylation changes > 5%. In the present study, fitness-related DMPs were depleted across active promoters, which is consistent with enrichment reported by Lindholm et al*.* among enhancers, gene bodies, and intergenic regions [4]. In addition, we, as well as others [4, 57], detected only modest (< 6%) effect sizes with training, suggesting that Robinson et al. were unable to detect exercise-induced changes because of their stringent effect size threshold and limited genome coverage.

### Proteome and training

Despite the DNA methylome not exhibiting a sex-specific response to training, the proteome responded in a sex-specific manner. Studies in males [5, 10, 12–14] and one study including both males and females [11] have found that various exercise training interventions induce skeletal muscle proteomic changes, however no studies to our knowledge have investigated proteomic sex differences at baseline or in response to exercise training in skeletal muscle [59]. Apart from Hostrup et al. which included 21 males, the abovementioned proteome-wide studies had relatively small sample sizes (< 12 total participants), and the only study including both sexes comprised six healthy controls and six type II diabetics (overall only four females); therefore making it challenging to elucidate potential sex differences with the existing proteomic data available. In the current study, the proteins which changed following training in a sex-specific manner were enriched for pathways involved in protein metabolism, which has frequently been shown to display sex differences at rest as well as during/following exercise across various layers of physiology and gene regulation [60]. Specifically, at the RNA level, male-biased gene transcripts in resting skeletal muscle are enriched for protein catabolism [15]. At the more fundamental physiological level, males excrete less urea in urine than females following exercise [61], indicating higher oxidation of proteins in males urea excretion, as urea excretion is used to estimate the relative contribution of amino acid oxidation to intermediary metabolism [60, 61]. In the sex-specific proteomic response to training, we found that ribosomal, mitochondrial, and RNA binding/splicing pathways were enriched among proteins associated with sex and training, shedding light on mechanisms in which sex-specific regulation should be further investigated. Sex-specific mRNA splicing [15] and transcription factor binding [17] have been reported in human skeletal muscle, suggesting that various aspects of gene regulation display sex bias. However, our data suggest that sex-specific gene regulation via DNA methylation is not apparent following training. The lack of overlap between sex-specific training proteins (current study) and previously identified sex-specific training mRNA [21] suggests that the various layers of gene regulation (i.e. DNA methylation, transcription, translation) have intricate roles in affecting consequent biological function and that they do not mirror one another. This is likely conserved across species, as studies in bacterial growth report low correlation between transcriptome and proteome, indicating that changes in the proteome are highly influenced by pre-translational regulators, such as small non-coding RNAs [62]. In addition to the effect of sex on the muscle proteome, the effect of fibre- and cell-type must also be taken into consideration in protein-wide analysis. Given fibre type-specific protein expression patterns both at rest and in response to training [5], it was important to elucidate whether the differences in fibre type proportions between the sexes contributed to the observed proteomic sex differences. The insignificant overlap between proteins differentially expressed between the sexes following training (current study) and those differentially expressed between the fibre types following training [5] implies that the observed sex-specific proteomic response to training was not due to differences in fibre type proportions.

### DNA methylome and CRF

In our cross-sectional sample of healthy individuals, CRF reflects years, if not lifelong patterns of regular physical activity. Although *V*O_2_max capacity is partially (~ 22–57%) inherited [63], nevertheless, it is representative of lifelong endurance training in various cohorts [64–66]. Individuals with higher CRF displayed distinct DNA methylation patterns, with no sex-specific differences. This suggests that lifelong physical activity induces similar changes in the male and female muscle methylomes, which is consistent with the lack of sex-specific response to training observed. CRF-associated regions were mostly found to be hypermethylated and were enriched in enhancers and regions flanking active promoters while depleted in active promoters. In contrast, Sailani et al. found that lifelong physical activity was associated with promoter hypomethylation in older healthy men [67]; discrepancies between our results and theirs may be due to the differences in genomic coverage owing to the utilisation of a different DNA methylation technique, as well as age and sex of participants. Studies comparing other -omic levels across people of varying fitness levels is warranted to understand the effect of lifelong training on molecular signatures in skeletal muscle.

### Proteome and CRF

There are limited studies which have investigated the effect of training status in males [68] (*n* = 5 trained; 5 untrained) or in both sexes [69] (*n* = 42 total; 11 trained/young, 11 sedentary/young, 10 trained/old, 10 sedentary/old) on protein-wide expression and none have investigated for potential sex differences. Both studies identified proteins which differ between trained and untrained individuals. The only study to include females did not state whether they took sex into consideration in their statistical analysis [69], despite the recognised effect of sex on other skeletal muscle-omic profiles [19]. In the current study, we did not identify sex-specific proteomic patterns associated with CRF; moreover, the absence of changes associated with CRF suggest that protein expression in the muscle may not reflect lifelong adaptations.

### Integration of -omic layers (CRF and training)

Integration of -omic layers may provide deeper insight on affected molecular pathways than a single -omic level, as all tested genes are inputted and ranked for the integration analysis (as opposed to simply identifying significant genes in one layer). The integration of proteome and methylome identified mitochondrial energy metabolism-related pathways, such as TCA cycle, respiratory electron transport, and mitochondrial biogenesis, which may be regulated differently between males and females both following a training intervention and in association with CRF. Similar pathways were enriched among sex-specific training and sex-specific CRF genes. This suggests that while the proteome appears to respond to acute adaptations in a sex-specific manner for the abovementioned pathways, the sex-specific effect of sustained training is only observed with the integration, and affects the same pathways as acute training. Irrespective of sex, the modest effect of exercise training and the robust effect of CRF on the DNA methylome suggest that exercise-induced DNA methylation changes are dependent on the sustained stimuli (i.e. training over more extended periods leads to more pronounced epigenetic changes in muscle); wheras the robust effect of exercise training and the absence of effect of CRF on the proteome suggest that exercise-induced protein changes are more acute and transient in nature. Altogether, this sheds light on the differing and complex roles of various -omic layers in the molecular response and adaptation to exercise.

One other study investigated whether CRF modulates sex differences in skeletal muscle-omic profiles. Chapman et al*.* [70] reported that lifelong endurance training diminishes transcriptomic sex differences in skeletal muscle. However, this study did not analyse for an interaction between training status and sex, rather, the number of differentially expressed genes between trained males and females was compared to the number of differentially expressed genes between untrained males and females. Therefore, no firm conclusions can be drawn from the study. For the first time, we report that exercise training shifts the DNA methylomes of males and females to appear more similar to one another.

The transcriptome has been thoroughly investigated for sex-specific changes following exercise training. In a single cohort (12 males and 11 females), no transcriptomic differences were observed between the sexes following training despite baseline transcriptomic differences [4]. However, utilising a meta-analysis (409 males and 310 females), Amar et al*.* detected sex differences in the transcriptomic response to training (247 genes). Thus, it cannot be excluded that additional cohorts could provide sufficient power to detect sex differences in the DNA methylome in response to training. Altogether, our findings indicate that short-term training similarly alters the male and female skeletal muscle methylomes. However, the paucity of studies on the topic means it is too early to draw firm conclusions.

### Strengths and limitations

Participants in our human cohort ranged from sedentary to recreationally active, to exceptionally active (one male and one female outliers). This heterogeneity in baseline fitness levels may limit our ability to detect changes in the DNA methylome and proteome following training, as changes in physiological and molecular measures may differ in magnitude depending on the exercise training history of the individual. To address this potential limitation, we assessed whether DNA methylation and protein changes associated with training were also associated with CRF (time-by-CRF interaction). Although we found no significant loci, the inflation of near zero *p*-values for the methylome, but not the proteome, suggests that there may be an association between baseline fitness levels and level of DNA methylation training response but that we were underpowered to detect it. This further supports our observation that lifelong fitness is better-characterised by the DNA methylome, as the DNA methylomic response to training was potentially influenced by baseline CRF, and not the same is true of the more transient proteome. In contrast, a heterogeneous cohort might be advantageous as it better reflects the general population. Given that baseline CRF is associated with sex, the study presents collinearity issues. The inherent link between sex and CRF may explain why some of the presented p-value distributions are not flat and seem to have less low and less high p-values than expected (e.g., Fig. 2F), suggesting over-correction of the data. This is not a shortcoming of the analysis, rather, a limitation of the data due to small sample sizes. Although the current study had a sample size considerably larger than previous studies with human muscles (typically *n* = 7–10), larger sample sizes, multi-site studies and initiatives, and open access data sharing, are required to detect the shifts in the proteome and methylome achieved with exercise training.

### Perspectives and significance

Overall, these findings emphasise the need for taking sex into consideration in exercise physiology studies as we found that biological sex affects the proteome at baseline and its response to exercise training. Furthermore, our study sheds light on the similar epigenetic response between males and females to lifelong, sustained cardiorespiratory fitness. These findings support that biological sex has a large impact on skeletal muscle physiology, gene regulation, and exercise metabolism. A recent meta-analysis of the muscle transcriptome response to exercise training reports hundreds of genes differentially expressed between males and females in response to exercise training [21]. Thus, our study investigates two other important layers of genomic regulation that have yet to be investigated for potential sex differences in response to training. These findings highlight the complexity of various genomic layers in modulating the molecular response to exercise, as well as highlight the effect of sex on each -omic layer and its modulatory behaviour in exercise adaptations.

## Conclusions

Short-term exercise training induced robust and sex-specific changes in the proteome with few and sex-independent changes in the muscle methylome. In contrast, CRF, which represents lifelong physical activity patterns, was associated with marked and sex-independent DNA methylation signatures. Altogether, our study elucidated the effect of sex on the DNA methylome and proteome responses to exercising training in human skeletal muscle. Given that the majority of studies investigating the molecular response to exercise training have not taken sex into consideration, this study is pivotal in advancing our current knowledge of molecular exercise physiology.

### Supplementary Information

**Additional file 1**: **Figure S1.** Histogram of p-values for DNA methylation and protein expression for all tested CpGs and proteins. DNA methylation (meta-analysis) histograms are A-F; proteomics (Gene SMART) histograms are G-K. (A) P-value histogram for the effect of training, model DNAm ~ sex + timepoint + batch (Gene SMART) + lean/obese (E-MTAB-11282) + age + baseline VO2. (B) P-value histogram for the interaction of sex and the training, model DNAm ~ sex * timepoint + batch (Gene SMART) + lean/obese (E-MTAB-11282) + age + baseline VO2. (C) P-value histogram for the interaction of baseline VO2 (CRF) and training, model DNAm ~ sex + batch (Gene SMART) + lean/obese (E-MTAB-11282) + age + baseline VO2 * timepoint. (D) P-value histogram for the interaction of sex and baseline VO2 (CRF), model DNAm ~ timepoint + batch + age + baseline VO2 * sex. (E) P-value histogram for baseline VO2 (CRF), model DNAm ~ timepoint + batch + age + baseline VO2 + sex. (F) P-value histogram for the control time point (“CON”; one month control period) relative to the PRE time point (Gene SMART only, before starting the HIIT intervention), model DNAm ~ sex + timepoint + batch + age + baseline VO2. (G) P-value histogram for the effect of training, model protein expression ~ sex + timepoint + age + baseline VO2. (H) P-value histogram for the interaction of sex and the training, model protein expression ~ sex * timepoint + age + baseline VO2. (I) P-value histogram for the interaction of baseline VO2 (CRF) and training, model protein expression ~ sex + age + baseline VO2 * timepoint. (J) P-value histogram for the interaction of sex and baseline VO2 (CRF), model protein expression ~ timepoint + age + baseline VO2 * sex. (K) P-value histogram for baseline VO2 (CRF), model protein expression ~ timepoint + age + baseline VO2 + sex. **Figure S2.** Volcano plots for proteomics and DNA methylation associations with training regardless of sex, sex, and cardiorespiratory fitness (CRF) regardless of sex. (A) Volcano plot of DNA methylome association with training, irrespective of sex. Red dots denote differentially methylated positions (DMPs) whose methylation increased with training; blue dots denote DMPs whose methylation decreased with training; black dots denote insignificant CpGs. Boxplots are the methylation levels (beta values residuals for batch) of the DMP pointed to, before and after training in the Gene SMART cohort. (B) Volcano plot of DNA methylome association with CRF, irrespective of sex. Red dots denote differentially methylated positions (DMPs) whose methylation increased with baseline VO2max (CRF); blue dots denote DMPs whose methylation decreased with baseline VO2max (CRF); black dots denote insignificant CpGs. Scatterplots are the methylation levels of the DMP pointed to (beta values residuals for batch), plotted against baseline VO2max in the Gene SMART cohort. (C) Volcano plot of proteome association with training in the Gene SMART cohort, irrespective of sex. Red dots denote differentially expressed proteins whose expression increased with training; blue dots denote proteins whose expression decreased with training; black dots denote insignificant proteins. Boxplots are the protein levels of the protein pointed to, before and after training. (D) Volcano plot of proteome association with CRF, irrespective of sex in the Gene SMART cohort. Black dots denote insignificant proteins. **Figure S3.** Correlation plots of residuals from the chi2 test for baseline fitness-DMPs enriched among the differing (A) Roadmap Epigenome project chromatin states and (B) CpG island locations. Blue denotes enrichment and red denotes depletion. **Figure S4.** Proteomics data before and after normalisation and plex correction. Log_2_ intensities (A) before and (B) after normalisation. (C) PCA plot of all samples in Gene SMART cohort after VSN normalisation and plex correction, each colour denotes a plex. Samples used in the manuscript were subsetted from a larger proteomics study. Figure S5 Sensitivity analyses for DNA methylation analysis. (A) Sensitivity analysis for using all covariates in one linear model or using a separate linear model (original) to detect delicate interaction between CRF and sex. 2D plot of effect sizes of each CpG for sex:CRF when comparing using one model (~ timepoint*sex + age + batch + baselineVO2*sex) vs a separate model (~ timepoint + age + batch + baselineVO2*sex). (B) Sensitivity analysis for using all covariates in one linear model or using a separate linear model (original) to detect delicate interaction between training and sex. 2D plot of effect sizes of each CpG for sex:training when comparing using one model (~ timepoint*sex + age + batch + baselineVO2*sex) vs a separate model (~ timepoint*sex + age + batch + baselineVO2). (C/D) Sensitivity analysis to ensure that a potential batch effect was not influencing our main findings, as one of the two batches in the Gene SMART data contained only males. We limited our analysis to only batch 2 in the Gene SMART study, which contained both males and females. We compared the effect sizes for coefficients of (C) sex:training and (D) sex:CRF (baseline VO2max) for all Gene SMART data (original) vs batch 2 of Gene SMART data at all CpGs. (E/F) Sensitivity analysis for effect of CRF on the DNA methylome by comparing to PRE-training samples only. 2D plot of effect sizes of (E) each CpG and (F) DMPs (FDR < 0.005) for effect of CRF on the DNA methylome, regardless of sex in Gene SMART cohort in all samples vs the Gene SMART cohort limited to PRE samples. Effect sizes are of beta values, FDR from M values.

**Additional file 2:** All results for: (1) proteins associated with sex*training. (2) proteins associated with sex, regardless of training or CRF. (3) proteins associated with training, regardless of sex. (4) proteins associated with CRF, regardless of sex. (5) proteins associated with sex*CRF. (6) DMPs associated with sex*training. (7) DMPs associated with sex*CRF. (8) DMPs associated with training, regardless of sex. (9) DMPs associated with CRF, regardless of sex. (10) enriched GO terms for DMPs with training, regardless of sex. (11) enriched GO terms for DMPs with CRF, regardless of sex. (12) enriched Reactome pathways for DMPs with training, regardless of sex. (13) enriched Reactome pathways for DMPs with CRF, regardless of sex. (14) enriched Reactome pathways for proteins with sex*training. (15) enriched GO terms (molecular function, cellular component, biological process) for proteins with sex*training. (16) enriched KEGG pathways for proteins with sex*training. (17) enriched Reactome pathways for proteins with training, regardless of sex. (18) enriched GO terms for proteins with training, regardless of sex. (19) enriched KEGG pathways for proteins with training, regardless of sex. (20) enriched Reactome pathways for proteins with sex, regardless of training or CRF. (21) enriched GO terms for proteins with sex, regardless of training or CRF. (22) enriched KEGG pathways for proteins with sex, regardless of training or CRF.

**Additional file 3:** Integration of DNA methylome and proteome for sex*training, output from MITCH package.

**Additional file 4:**  Integration of DNA methylome and proteome for sex*CRF, output from MITCH package.

## Data Availability

Raw data are publically available on GEO (GSE171140) and code is available on GitHub (https://github.com/shanie-landen/sex-specific-exercise).

## Abbreviations

CRF: Cardiorespiratory fitness
DMG: Differentially methylated gene
DMP: Differentially methylated position
DMR: Differentially methylated region
FDR: False discovery rate
Gene SMART: Skeletal Muscle Adaptive Response to Training
GO: Gene Ontology
HIIT: High-intensity interval training
LT: Lactate threshold
MICT: Moderate-intensity continuous endurance training
PCA: Principal component analysis
PP: Peak power
*V*O_2_max: Maximum oxygen consumption
