# Supplementary material for: Sex differences in muscle protein expression and DNA methylation in response to exercise training
Source: Biol Sex Differ. 2023 Sep 5;14:56. doi: 10.1186/s13293-023-00539-2 (PMC10478435; doi:10.1186/s13293-023-00539-2)
Supplement: Supplementary file 1 — Additional file 1: Figure S1. Histogram of p-values for DNA methylation and protein expression for all tested CpGs and proteins. DNA methylation (meta-analysis) histograms are A-F; proteomics (Gene SMART) histograms are G-K. (A) P-value histogram for the effect of training, model DNAm ~ sex + timepoint + batch (Gene SMART) + lean/obese (E-MTAB-11282) + age + baseline VO2. (B) P-value histogram for the interaction of sex and the training, model DNAm ~ sex * timepoint + batch (Gene SMART) + lean/obese (E-MTAB-11282) + age + baseline VO2. (C) P-value histogram for the interaction of baseline VO2 (CRF) and training, model DNAm ~ sex + batch (Gene SMART) + lean/obese (E-MTAB-11282) + age + baseline VO2 * timepoint. (D) P-value histogram for the interaction of sex and baseline VO2 (CRF), model DNAm ~ timepoint + batch + age + baseline VO2 * sex. (E) P-value histogram for baseline VO2 (CRF), model DNAm ~ timepoint + batch + age + baseline VO2 + sex. (F) P-value histogram for the control time point (“CON”; one month control period) relative to the PRE time point (Gene SMART only, before starting the HIIT intervention), model DNAm ~ sex + timepoint + batch + age + baseline VO2. (G) P-value histogram for the effect of training, model protein expression ~ sex + timepoint + age + baseline VO2. (H) P-value histogram for the interaction of sex and the training, model protein expression ~ sex * timepoint + age + baseline VO2. (I) P-value histogram for the interaction of baseline VO2 (CRF) and training, model protein expression ~ sex + age + baseline VO2 * timepoint. (J) P-value histogram for the interaction of sex and baseline VO2 (CRF), model protein expression ~ timepoint + age + baseline VO2 * sex. (K) P-value histogram for baseline VO2 (CRF), model protein expression ~ timepoint + age + baseline VO2 + sex. Figure S2. Volcano plots for proteomics and DNA methylation associations with training regardless of sex, sex, and cardiorespiratory fitness (CRF) regardless of sex. (A) Vol [file 13293_2023_539_MOESM1_ESM.docx]

## Additional file 1: Figures


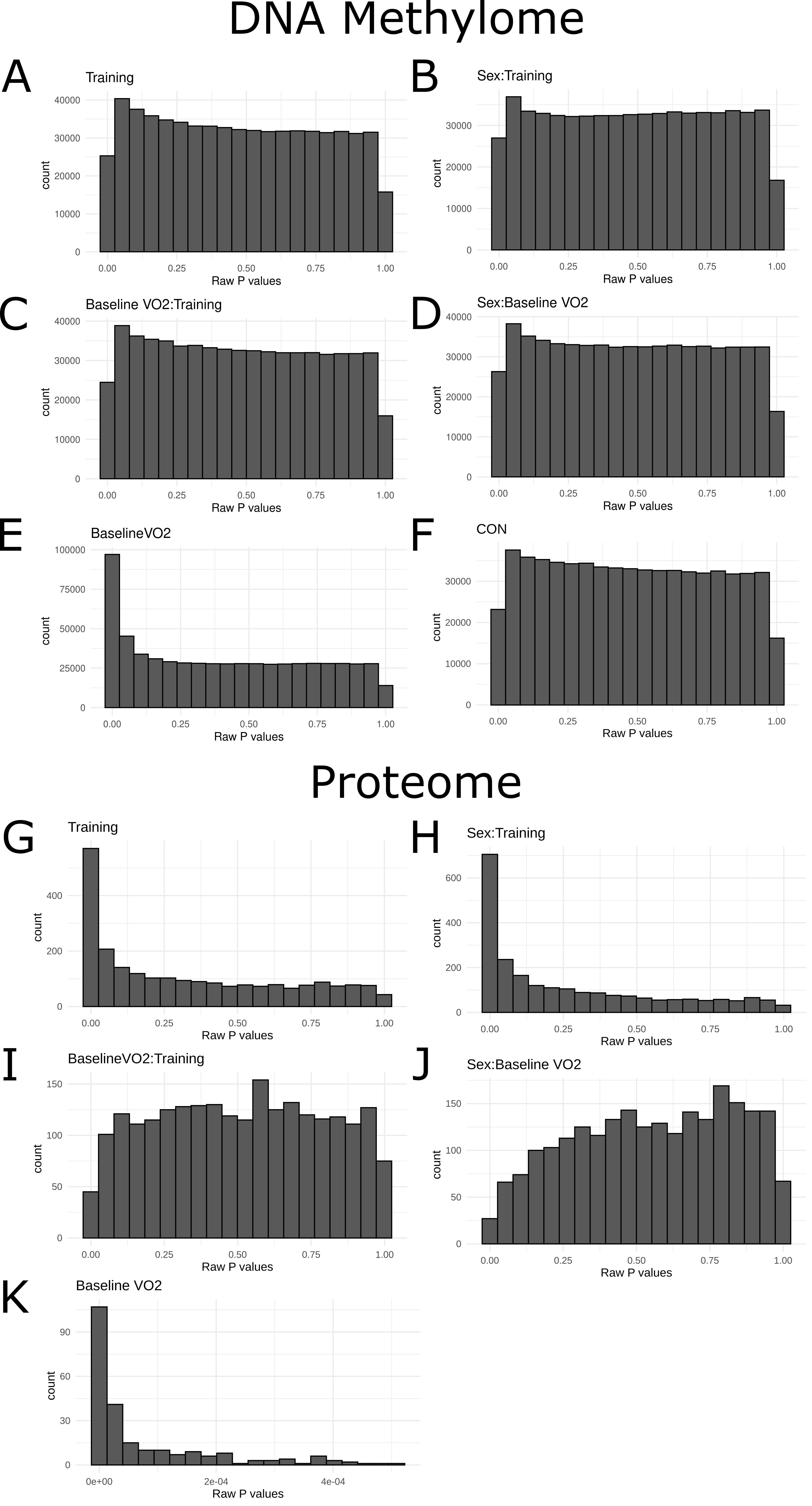


Figure S1. Histogram of p-values for DNA methylation and protein expression for all tested CpGs and proteins.

DNA methylation (meta-analysis) histograms are A-F; proteomics (Gene SMART) histograms are G-K. (A) P-value histogram for the effect of training , model DNAm ~ sex + timepoint + batch (Gene SMART) + lean/obese (E-MTAB-11282) + age + baseline VO2. (B) P-value histogram for the interaction of sex and the training, model DNAm ~ sex * timepoint + batch (Gene SMART) + lean/obese (E-MTAB-11282) + age + baseline VO2. (C) P-value histogram for the interaction of baseline VO2 (CRF) and training, model DNAm ~ sex + batch (Gene SMART) + lean/obese (E-MTAB-11282) + age + baseline VO2 * timepoint. (D) P-value histogram for the interaction of sex and baseline VO2 (CRF), model DNAm ~ timepoint + batch + age + baseline VO2 * sex. (E) P-value histogram for baseline VO2 (CRF), model DNAm ~ timepoint + batch + age + baseline VO2 + sex. (F) P-value histogram for the control time point (“CON”; one month control period) relative to the PRE time point (Gene SMART only, before starting the HIIT intervention), model DNAm ~ sex + timepoint + batch + age + baseline VO2. (G) P-value histogram for the effect of training, model protein expression ~ sex + timepoint + age + baseline VO2. (H) P-value histogram for the interaction of sex and the training, model protein expression ~ sex * timepoint + age + baseline VO2. (I) P-value histogram for the interaction of baseline VO2 (CRF) and training, model protein expression ~ sex + age + baseline VO2 * timepoint. (J) P-value histogram for the interaction of sex and baseline VO2 (CRF), model protein expression ~ timepoint + age + baseline VO2 * sex. (K) P-value histogram for baseline VO2 (CRF), model protein expression ~ timepoint + age + baseline VO2 + sex.


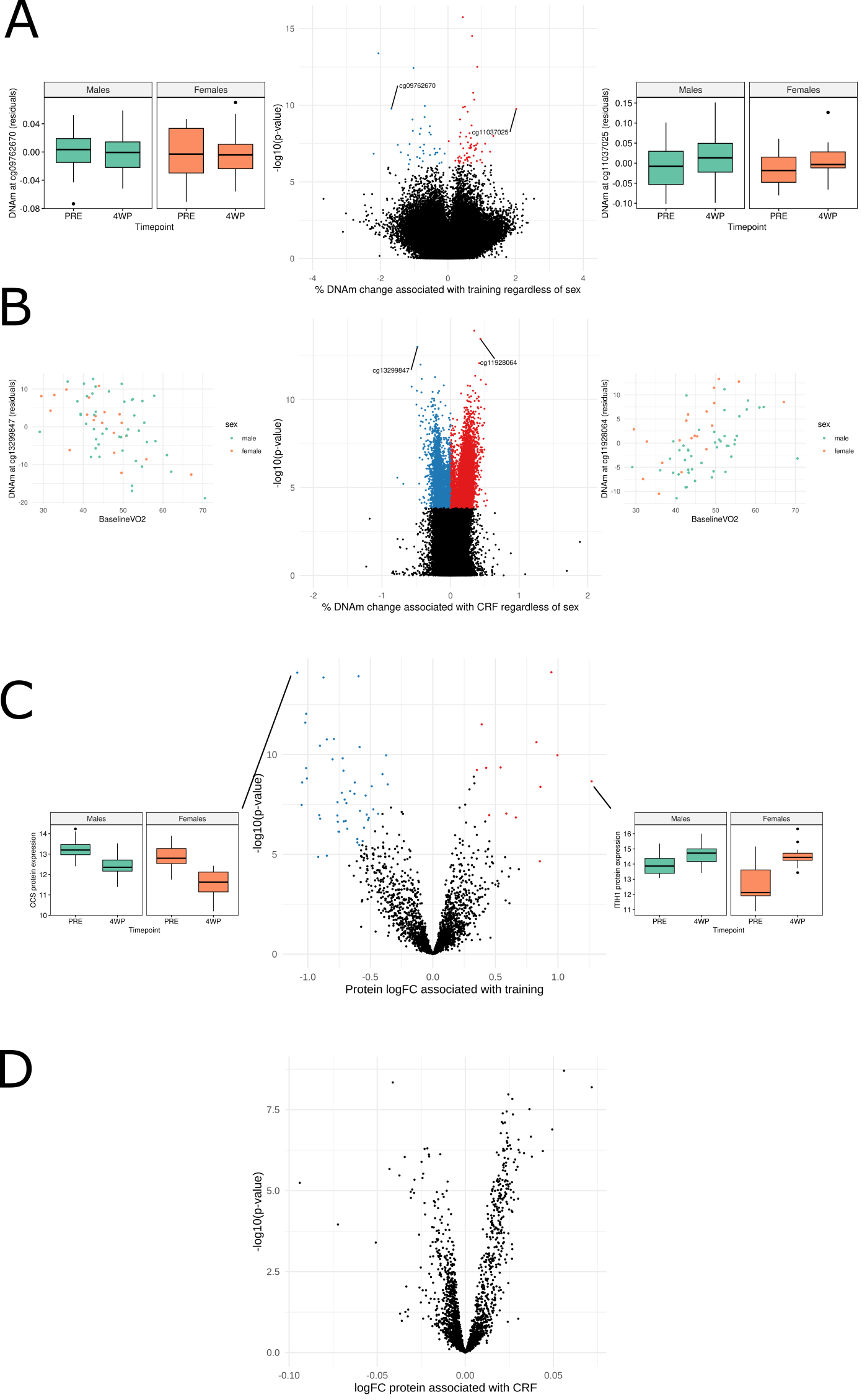


Figure S2 Volcano plots for proteomics and DNA methylation associations with training regardless of sex, sex, and cardiorespiratory fitness (CRF) regardless of sex.

**(A)** Volcano plot of DNA methylome association with training, irrespective of sex. Red dots denote differentially methylated positions (DMPs) whose methylation increased with training; blue dots denote DMPs whose methylation decreased with training; black dots denote insignificant CpGs. Boxplots are the methylation levels (beta values residuals for batch) of the DMP pointed to, before and after training in the Gene SMART cohort. **(B)** Volcano plot of DNA methylome association with CRF, irrespective of sex. Red dots denote differentially methylated positions (DMPs) whose methylation increased with baseline VO2max (CRF); blue dots denote DMPs whose methylation decreased with baseline VO2max (CRF); black dots denote insignificant CpGs. Scatterplots are the methylation levels of the DMP pointed to (beta values residuals for batch), plotted against baseline VO2max in the Gene SMART cohort. **(C)** Volcano plot of proteome association with training in the Gene SMART cohort, irrespective of sex. Red dots denote differentially expressed proteins whose expression increased with training; blue dots denote proteins whose expression decreased with training; black dots denote insignificant proteins. Boxplots are the protein levels of the protein pointed to, before and after training. **(D)** Volcano plot of proteome association with CRF, irrespective of sex in the Gene SMART cohort. Black dots denote insignificant proteins.


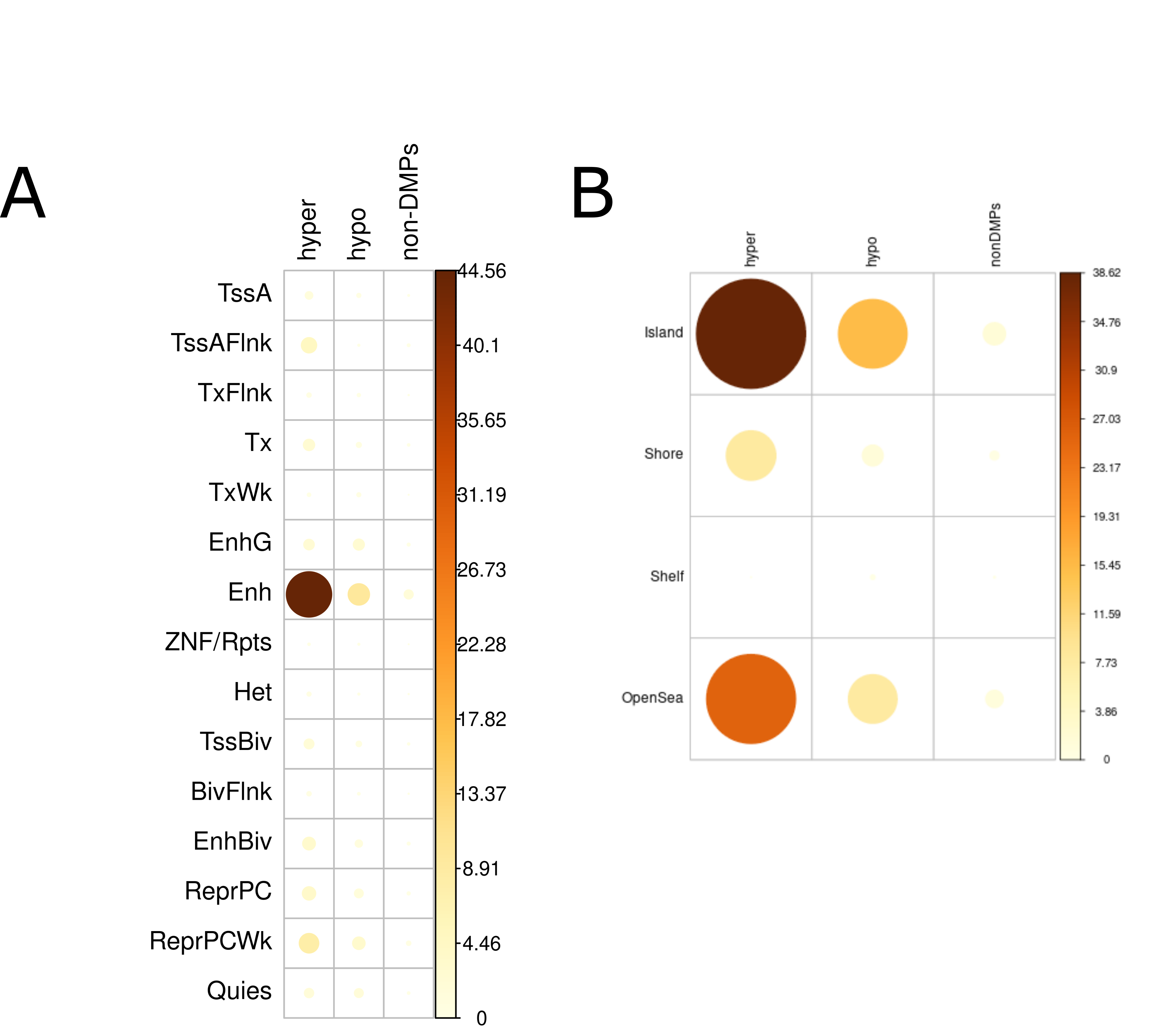


Figure S3 Correlation plots of residuals from the chi2 test for baseline fitness-DMPs enriched among the differing (A) Roadmap Epigenome project chromatin states and (B) CpG island locations.

Blue denotes enrichment and red denotes depletion.


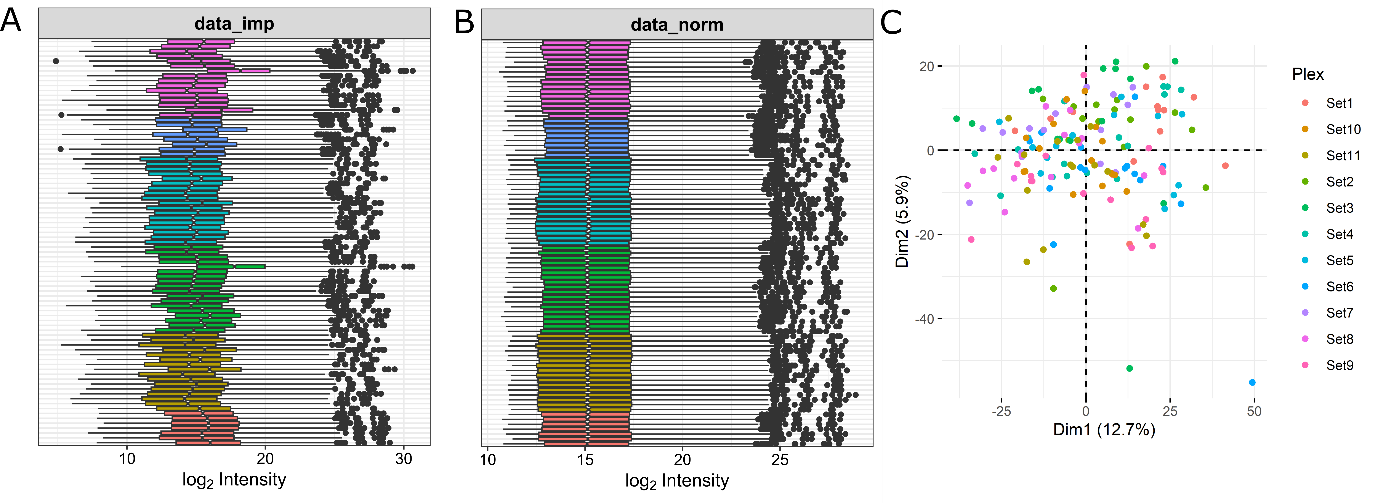


Figure S4 Proteomics data before and after normalization and plex correction. Log_2_ intensities (A) before and (B) after normalization. (C) PCA plot of all samples in Gene SMART cohort after VSN normalization and plex correction, each color denotes a plex. Samples used in the manuscript were subsetted from a larger proteomics study.


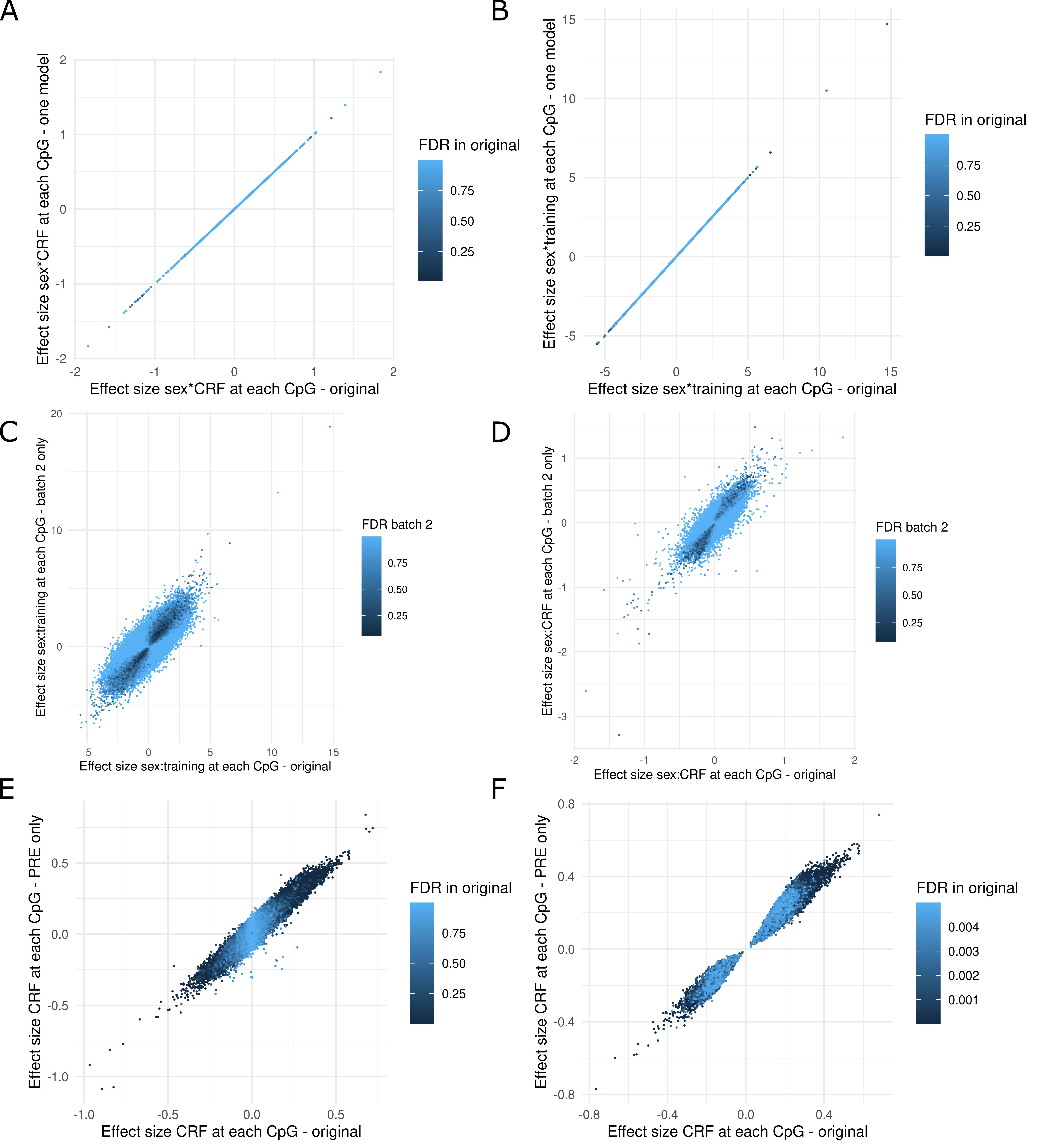


**Figure S5 Sensitivity analyses for DNA methylation analysis. (A) Sensitivity analysis for using all covariates in one linear model or using a seperate linear model (original) to detect delicate interaction between CRF and sex. 2D plot of effect sizes of each CpG for sex:CRF when comparing using one model (~ timepoint*sex + age + batch+ baselineVO2*sex) vs a separarate model (~ timepoint + age + batch + baselineVO2*sex). (B) Sensitivity analysis for using all covariates in one linear model or using a seperate linear model (original) to detect delicate interaction between training and sex. 2D plot of effect sizes of each CpG for sex:training when comparing using one model (~ timepoint*sex + age + batch+ baselineVO2*sex) vs a separarate model (~ timepoint*sex + age + batch + baselineVO2). (C/D) Sensitivity analysis to ensure that a potential batch effect was not influencing our main findings, as one of the two batches in the Gene SMART data contained only males. We limited our analysis to only batch 2 in the Gene SMART study, which contained both males and females. We compared the effect sizes for coefficients of (C) sex:training and (D) sex:CRF (baseline VO2max) for all Gene SMART data (original) vs batch 2 of Gene SMART data at all CpGs. (E/F) Sensitivity analysis for effect of CRF on the DNA methylome by comparing to PRE-training samples only. 2D plot of effect sizes of (E) each CpG and (F) DMPs (FDR < 0.005) for effect of CRF on the DNA methylome, regardless of sex in Gene SMART cohort in all samples vs the Gene SMART cohort limited to PRE samples. Effect sizes are of beta values, FDR from M values.**

# References

1. Fiuza-Luces, C., et al., *Exercise is the real polypill.* Physiology, 2013.

2. Sanford, J.A., et al., *Molecular transducers of physical activity consortium (MoTrPAC): Mapping the dynamic responses to exercise.* Cell, 2020. **181**(7): p. 1464-1474.

3. Jacques, M., et al., *Epigenetic changes in healthy human skeletal muscle following exercise–a systematic review.* Epigenetics, 2019. **14**(7): p. 633-648.

4. Lindholm, M.E., et al., *An integrative analysis reveals coordinated reprogramming of the epigenome and the transcriptome in human skeletal muscle after training.* Epigenetics, 2014. **9**(12): p. 1557-1569.

5. Deshmukh, A., et al., *Deep muscle-proteomic analysis of freeze-dried human muscle biopsies reveals fiber type-specific adaptations to exercise training.* Nature communications, 2021. **12**(1): p. 1-15.

6. Hughes, D.C., S. Ellefsen, and K. Baar, *Adaptations to endurance and strength training.* Cold Spring Harbor perspectives in medicine, 2018. **8**(6): p. a029769.

7. Landen, S., et al., *Physiological and molecular sex differences in human skeletal muscle in response to exercise training.* The Journal of Physiology, 2021.

8. Costello, J.T., F. Bieuzen, and C.M. Bleakley, *Where are all the female participants in Sports and Exercise Medicine research?* European Journal of Sport Science, 2014. **14**(8): p. 847-851.

9. Hagstrom, A.D., et al., *Sex bias in cohorts included in sports medicine research.* Sports Medicine, 2021: p. 1-6.

10. Granata, C., et al., *High-intensity training induces non-stoichiometric changes in the mitochondrial proteome of human skeletal muscle without reorganisation of respiratory chain content.* Nature communications, 2021. **12**(1): p. 1-18.

11. Hussey, S.E., et al., *Effect of exercise on the skeletal muscle proteome in patients with type 2 diabetes.* Medicine and science in sports and exercise, 2013. **45**(6): p. 1069.

12. Holloway, K.V., et al., *Proteomic investigation of changes in human vastus lateralis muscle in response to interval‐exercise training.* Proteomics, 2009. **9**(22): p. 5155-5174.

13. Egan, B., et al., *2-D DIGE analysis of the mitochondrial proteome from human skeletal muscle reveals time course-dependent remodelling in response to 14 consecutive days of endurance exercise training.* Proteomics, 2011. **11**(8): p. 1413-28.

14. Hostrup, M., et al., *Chronic β2‐adrenoceptor agonist treatment alters muscle proteome and functional adaptations induced by high intensity training in young men.* The Journal of physiology, 2018. **596**(2): p. 231-252.

15. Lindholm, M.E., et al., *The human skeletal muscle transcriptome: sex differences, alternative splicing, and tissue homogeneity assessed with RNA sequencing.* Faseb j, 2014. **28**(10): p. 4571-81.

16. Welle, S., R. Tawil, and C.A. Thornton, *Sex-Related Differences in Gene Expression in Human Skeletal Muscle.* PLoS One, 2008. **3**(1).

17. Lopes-Ramos, C.M., et al., *Sex Differences in Gene Expression and Regulatory Networks across 29 Human Tissues.* Cell Reports, 2020. **31**(12): p. 107795.

18. Gershoni, M. and S. Pietrokovski, *The landscape of sex-differential transcriptome and its consequent selection in human adults.* BMC Biology, 2017. **15**(1): p. 7.

19. Oliva, M., et al., *The impact of sex on gene expression across human tissues.* Science, 2020. **369**(6509): p. eaba3066.

20. Landen, S., et al., *Skeletal muscle methylome and transcriptome integration reveals profound sex differences related to muscle function and substrate metabolism.* Clinical epigenetics, 2021. **13**(1): p. 1-20.

21. Amar, D., et al., *Time trajectories in the transcriptomic response to exercise-a meta-analysis.* Nature communications, 2021. **12**(1): p. 1-12.

22. Milanović, Z., G. Sporiš, and M. Weston, *Effectiveness of high-intensity interval training (HIT) and continuous endurance training for VO2max improvements: a systematic review and meta-analysis of controlled trials.* Sports medicine, 2015. **45**(10): p. 1469-1481.

23. McMURRAY, R.G., et al., *Is physical activity or aerobic power more influential on reducing cardiovascular disease risk factors?* Medicine and science in sports and exercise, 1998. **30**(10): p. 1521-1529.

24. Voisin, S., et al., *An epigenetic clock for human skeletal muscle.* Journal of cachexia, sarcopenia and muscle, 2020. **11**(4): p. 887-898.

25. Landen, S., et al., *Genome-wide DNA methylation and transcriptome integration reveal distinct sex differences in skeletal muscle.* bioRxiv, 2021.

26. Voisin, S., et al., *Exercise is associated with younger methylome and transcriptome profiles in human skeletal muscle.* Aging Cell, 2023: p. e13859.

27. Yan, X., et al., *The gene SMART study: method, study design, and preliminary findings.* BMC Genomics, 2017. **18**(Suppl 8): p. 821.

28. Eynon, N. 2018; Available from: [www.vu.edu.au/speed-gene](file:///D:\Paper%203%20for%20submission\Paper%203\BOSD%20edits\www.vu.edu.au\speed-gene).

29. Mifflin, M.D., et al., *A new predictive equation for resting energy expenditure in healthy individuals.* The American journal of clinical nutrition, 1990. **51**(2): p. 241-247.

30. Burke, L., *Fasting and recovery from exercise.* British Journal of Sports Medicine, 2010. **44**(7): p. 502-508.

31. Mihm, M., S. Gangooly, and S. Muttukrishna, *The normal menstrual cycle in women.* Anim Reprod Sci, 2011. **124**(3-4): p. 229-36.

32. Godsland, I.F., et al., *The Effects of Different Formulations of Oral Contraceptive Agents on Lipid and Carbohydrate Metabolism.* New England Journal of Medicine, 1990. **323**(20): p. 1375-1381.

33. Smyth, G.K., *limma: Linear Models for Microarray Data*, in *Bioinformatics and Computational Biology Solutions Using R and Bioconductor*, R. Gentleman, et al., Editors. 2005, Springer New York: New York, NY. p. 397-420.

34. Xiao, Y., et al., *A novel significance score for gene selection and ranking.* Bioinformatics, 2014. **30**(6): p. 801-807.

35. Tian, Y., et al., *ChAMP: updated methylation analysis pipeline for Illumina BeadChips.* Bioinformatics, 2017. **33**(24): p. 3982-3984.

36. Pidsley, R., et al., *Critical evaluation of the Illumina MethylationEPIC BeadChip microarray for whole-genome DNA methylation profiling.* Genome biology, 2016. **17**(1): p. 1-17.

37. Chen, Y.-a., et al., *Cross-reactive DNA microarray probes lead to false discovery of autosomal sex-associated DNA methylation.* The American Journal of Human Genetics, 2012. **91**(4): p. 762-764.

38. Leek, J.T., et al., *Package ‘sva’.* 2014.

39. Benjamini, Y. and Y. Hochberg, *Controlling the false discovery rate: a practical and powerful approach to multiple testing.* Journal of the royal statistical society. Series B (Methodological), 1995: p. 289-300.

40. Benjamin, D.J., et al., *Redefine statistical significance.* Nature Human Behaviour, 2018. **2**(1): p. 6.

41. Peters, T.J., et al., *De novo identification of differentially methylated regions in the human genome.* Epigenetics & chromatin, 2015. **8**(1): p. 6.

42. van Iterson, M., E.W. van Zwet, and B.T. Heijmans, *Controlling bias and inflation in epigenome-and transcriptome-wide association studies using the empirical null distribution.* Genome biology, 2017. **18**(1): p. 1-13.

43. Leek, J.T., et al., *Tackling the widespread and critical impact of batch effects in high-throughput data.* Nature Reviews Genetics, 2010. **11**(10): p. 733-739.

44. Willer, C.J., Y. Li, and G.R. Abecasis, *METAL: fast and efficient meta-analysis of genomewide association scans.* Bioinformatics, 2010. **26**(17): p. 2190-2191.

45. Nitert, M.D., et al., *Impact of an exercise intervention on DNA methylation in skeletal muscle from first-degree relatives of patients with type 2 diabetes.* Diabetes, 2012. **61**(12): p. 3322-3332.

46. Robinson, M.M., et al., *Enhanced protein translation underlies improved metabolic and physical adaptations to different exercise training modes in young and old humans.* Cell metabolism, 2017. **25**(3): p. 581-592.

47. Zhou, W., P.W. Laird, and H. Shen, *Comprehensive characterization, annotation and innovative use of Infinium DNA methylation BeadChip probes.* Nucleic acids research, 2017. **45**(4): p. e22-e22.

48. Kundaje, A., et al., *Integrative analysis of 111 reference human epigenomes.* Nature, 2015. **518**(7539): p. 317-330.

49. Fishilevich, S., et al., *GeneHancer: genome-wide integration of enhancers and target genes in GeneCards.* Database, 2017. **2017**.

50. Phipson, B., J. Maksimovic, and A. Oshlack, *missMethyl: an R package for analyzing data from Illumina’s HumanMethylation450 platform.* Bioinformatics, 2016. **32**(2): p. 286-288.

51. Maksimovic, J., A. Oshlack, and B. Phipson, *Gene set enrichment analysis for genome-wide DNA methylation data.* bioRxiv, 2020.

52. Voisin, S., et al., *Meta‐analysis of genome‐wide DNA methylation and integrative omics of age in human skeletal muscle.* Journal of Cachexia, Sarcopenia and Muscle, 2021. **12**(4): p. 1064-1078.

53. Garcia, L.A., et al., *Can Exercise Training Alter Human Skeletal Muscle DNA Methylation?* Metabolites, 2022. **12**(3).

54. Rapp, D., et al., *Reference values for peak oxygen uptake: cross-sectional analysis of cycle ergometry-based cardiopulmonary exercise tests of 10 090 adult German volunteers from the Prevention First Registry.* BMJ open, 2018. **8**(3): p. e018697.

55. Haizlip, K., B. Harrison, and L. Leinwand, *Sex-based differences in skeletal muscle kinetics and fiber-type composition.* Physiology, 2015. **30**(1): p. 30-39.

56. Seaborne, R.A., et al., *Human skeletal muscle possesses an epigenetic memory of hypertrophy.* Scientific reports, 2018. **8**(1): p. 1-17.

57. Price, E.M. and W.P. Robinson, *Adjusting for batch effects in DNA methylation microarray data, a lesson learned.* Frontiers in genetics, 2018. **9**: p. 83.

58. Lindholm, M.E., et al., *An integrative analysis reveals coordinated reprogramming of the epigenome and the transcriptome in human skeletal muscle after training.* Epigenetics, 2014. **9**(12): p. 1557-69.

59. Srisawat, K., et al., *A systematic review and meta-analysis of proteomics literature on the response of human skeletal muscle to obesity/type 2 diabetes mellitus (T2DM) versus exercise training.* Proteomes, 2017. **5**(4): p. 30.

60. Tarnopolsky, M., *Sex differences in exercise metabolism and the role of 17-beta estradiol.* Medicine & Science in Sports & Exercise, 2008. **40**(4): p. 648-654.

61. Lamont, L.S., A.J. McCullough, and S.C. Kalhan, *Gender differences in the regulation of amino acid metabolism.* Journal of Applied Physiology, 2003. **95**(3): p. 1259-1265.

62. Bathke, J., et al., *Comparative analyses of the variation of the transcriptome and proteome of Rhodobacter sphaeroides throughout growth.* BMC genomics, 2019. **20**(1): p. 1-13.

63. Zadro, J.R., et al., *The Beneficial Effects of Physical Activity: Is It Down to Your Genes? A Systematic Review and Meta-Analysis of Twin and Family Studies.* Sports Medicine - Open, 2017. **3**(1): p. 4.

64. Carrick-Ranson, G., et al., *The effect of lifelong exercise dose on cardiovascular function during exercise.* Journal of Applied Physiology, 2014. **116**(7): p. 736-745.

65. Gates, P.E., et al., *Left ventricular structure and diastolic function with human ageing: relation to habitual exercise and arterial stiffness.* European heart journal, 2003. **24**(24): p. 2213-2220.

66. Ogawa, T., et al., *Effects of aging, sex, and physical training on cardiovascular responses to exercise.* Circulation, 1992. **86**(2): p. 494-503.

67. Sailani, M.R., et al., *Lifelong physical activity is associated with promoter hypomethylation of genes involved in metabolism, myogenesis, contractile properties and oxidative stress resistance in aged human skeletal muscle.* Scientific reports, 2019. **9**(1): p. 1-11.

68. Schild, M., et al., *Basal and exercise induced label-free quantitative protein profiling of m. vastus lateralis in trained and untrained individuals.* J Proteomics, 2015. **122**: p. 119-32.

69. Lanza, I.R., et al., *Endurance exercise as a countermeasure for aging.* Diabetes, 2008. **57**(11): p. 2933-2942.

70. Chapman, M.A., et al., *Skeletal Muscle Transcriptomic Comparison between Long-Term Trained and Untrained Men and Women.* Cell Reports, 2020. **31**(12): p. 107808.
