# Supplementary material for: Sex differences in muscle protein expression and DNA methylation in response to exercise training
Source: Biol Sex Differ. 2023 Sep 5;14:56. doi: 10.1186/s13293-023-00539-2 (PMC10478435; doi:10.1186/s13293-023-00539-2)
Supplement: Supplementary file 3 — Additional file 3: Integration of DNA methylome and proteome for sex*training, output from MITCH package. [file 13293_2023_539_MOESM3_ESM.pdf]

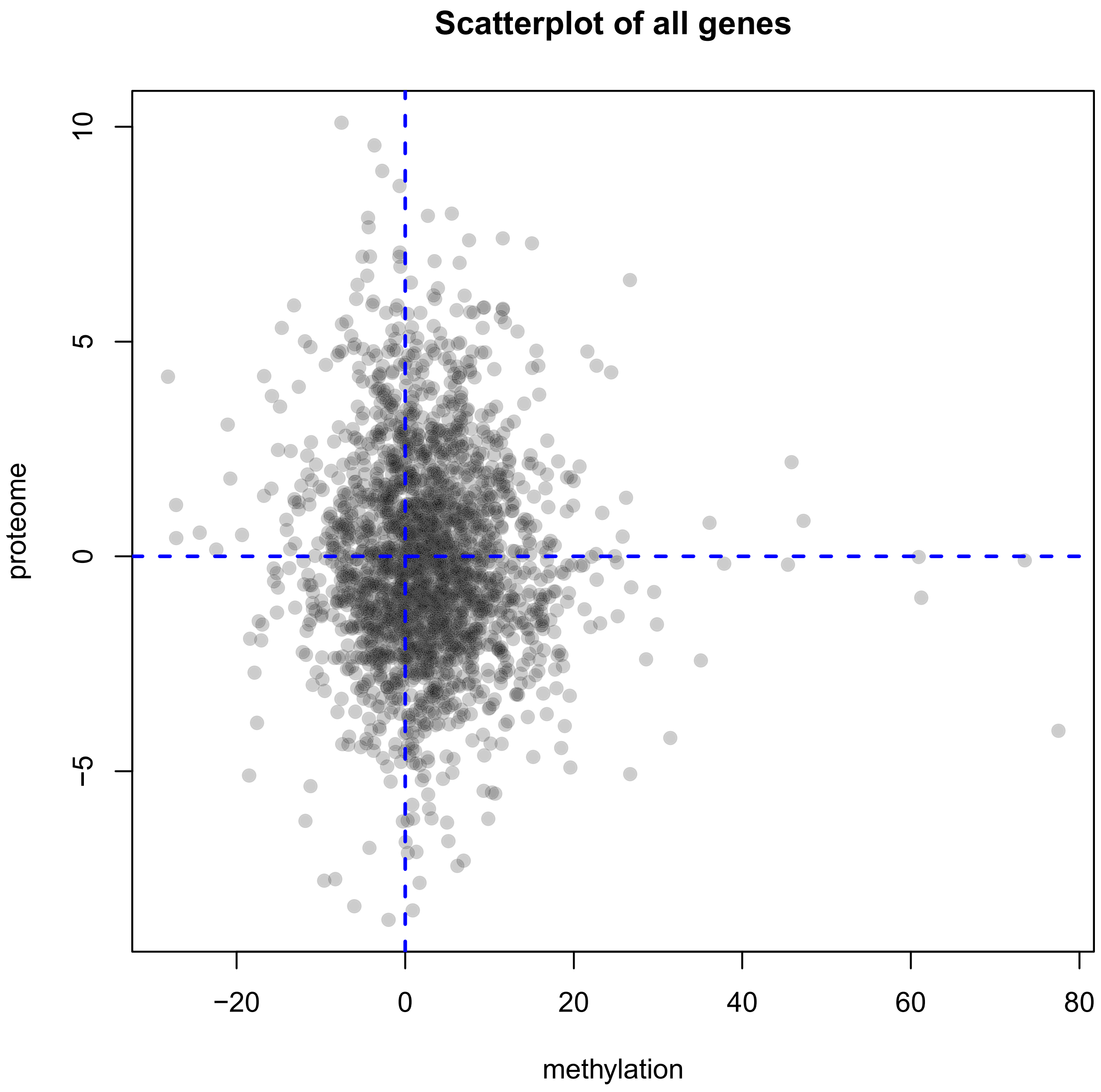
Scatterplot of all genes
proteome
methylation
10
5
0
−5
−20
0
20
40
60
80

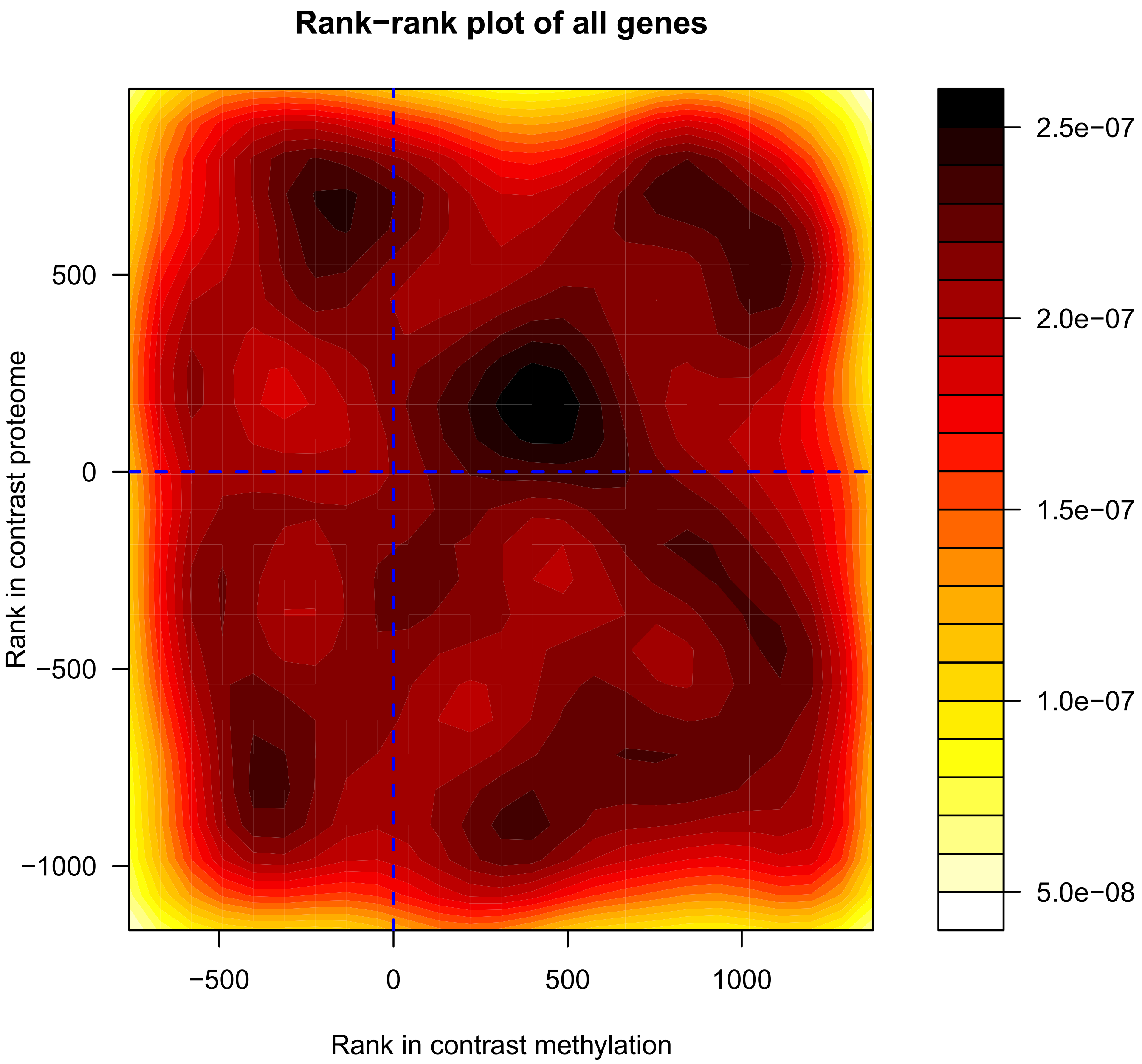

Rank−rank plot of all genes
Rank in contrast proteome
Rank in contrast methylation
500
0
−500
−1000
−500
0
500
1000
2.5e−07
2.0e−07
1.5e−07
1.0e−07
5.0e−08

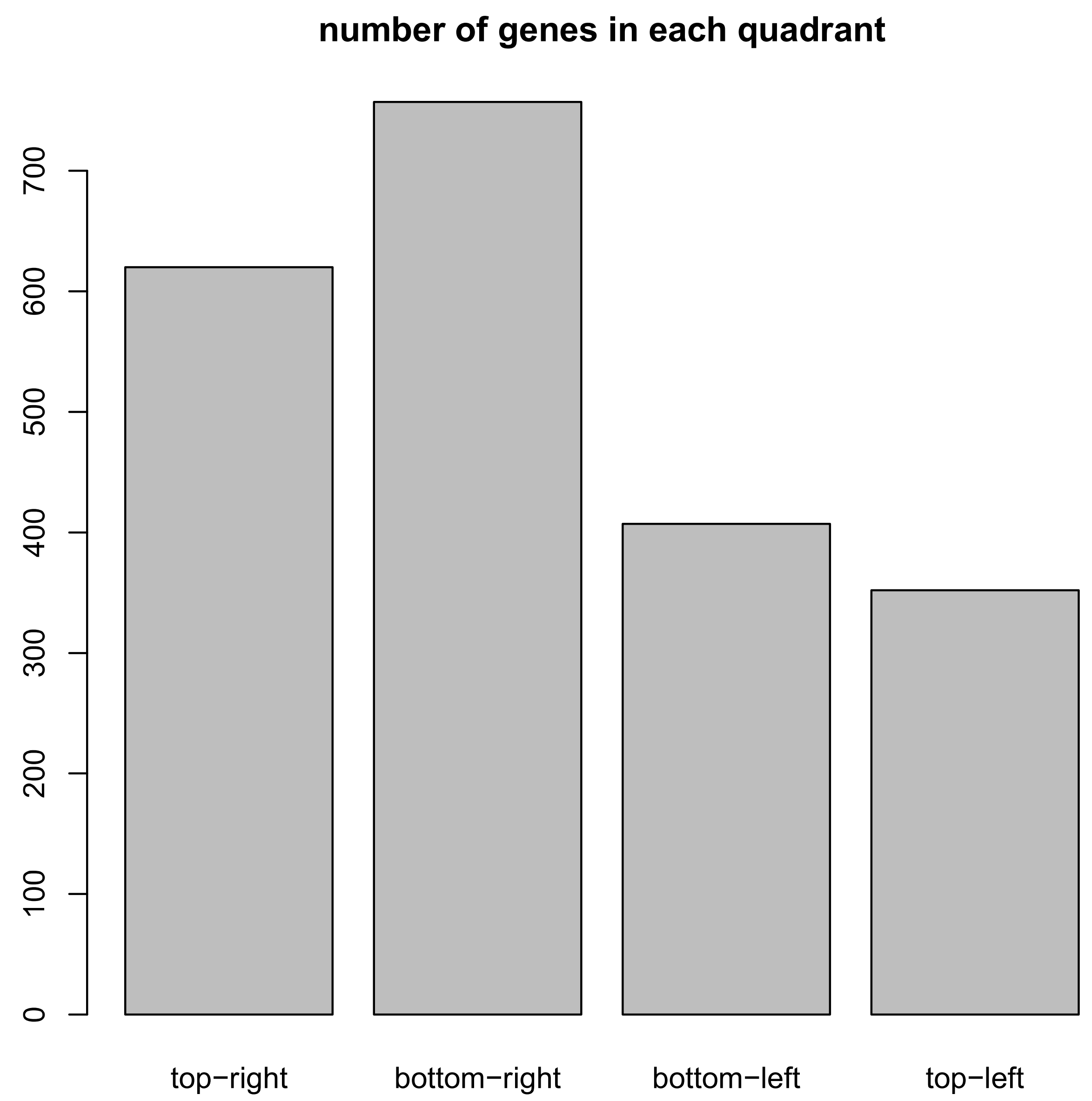
number of genes in each quadrant
0
100
200
300
400
500
600
700
top−right
bottom−right
bottom−left
top−left

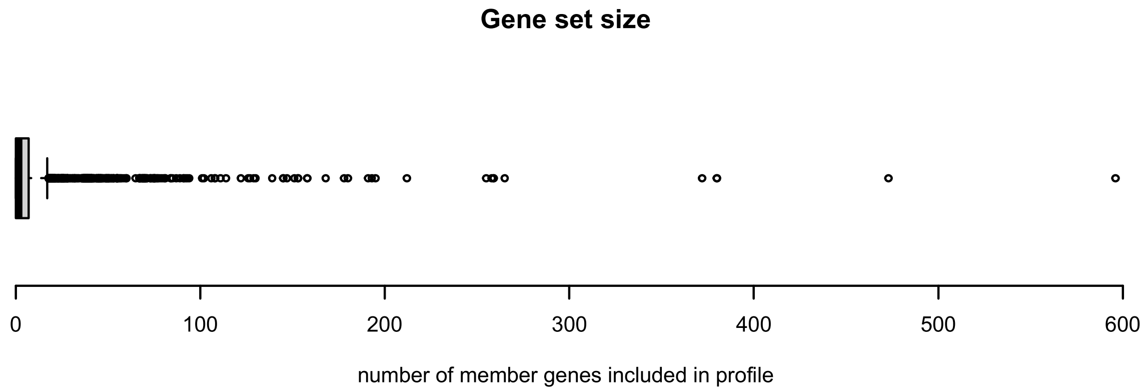
Gene set size
0
100
200
300
400
500
600
number of member genes included in profile

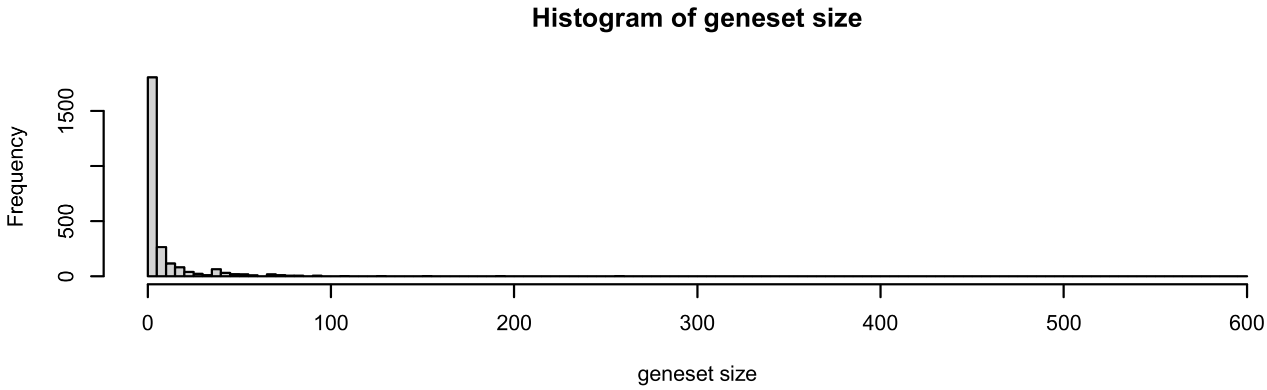
Histogram of geneset size
Frequency
0
500
1500
0
100
200
300
400
500
600
geneset size

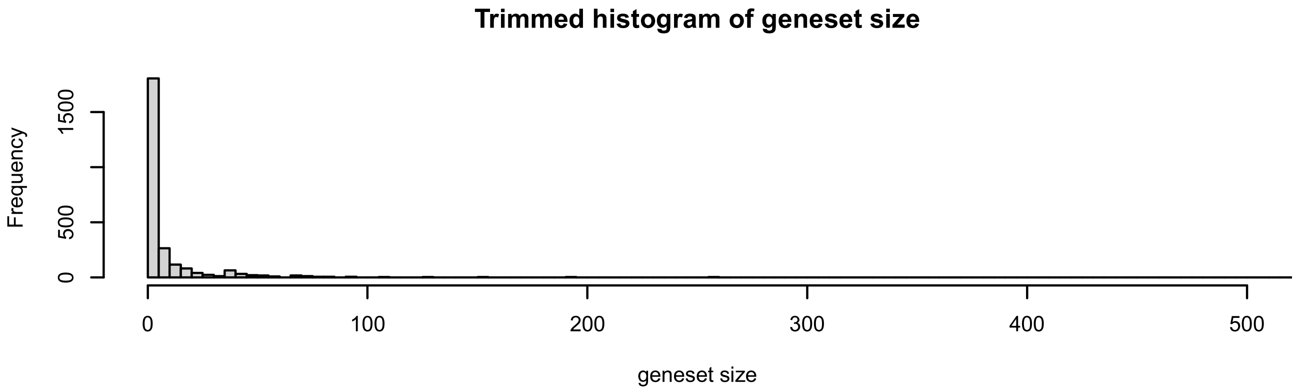
Trimmed histogram of geneset size
Frequency
0
500
1500
0
100
200
300
400
500
geneset size

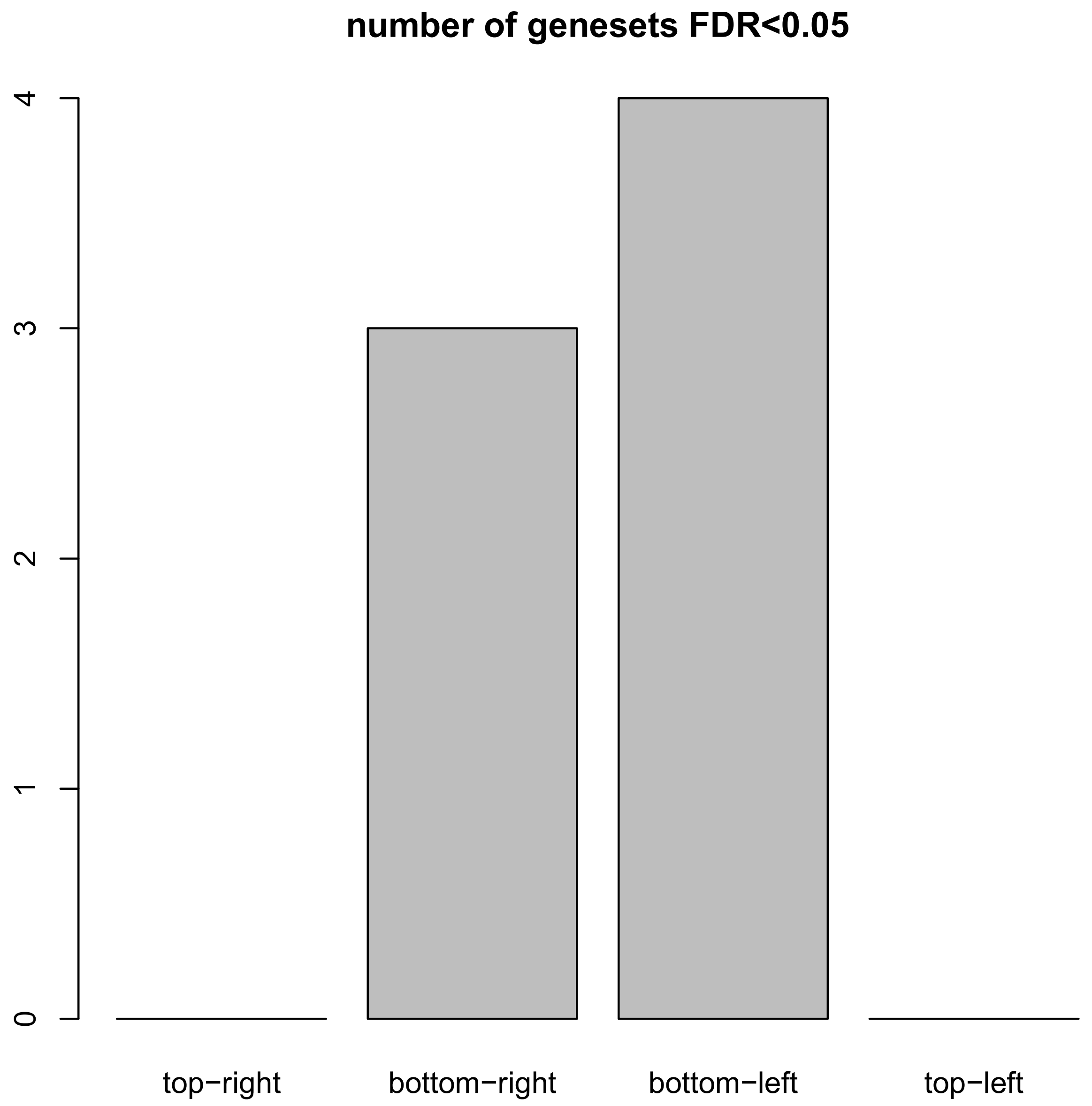
number of genesets FDR<0.05
0
1
2
3
4
top−right
bottom−right
bottom−left
top−left

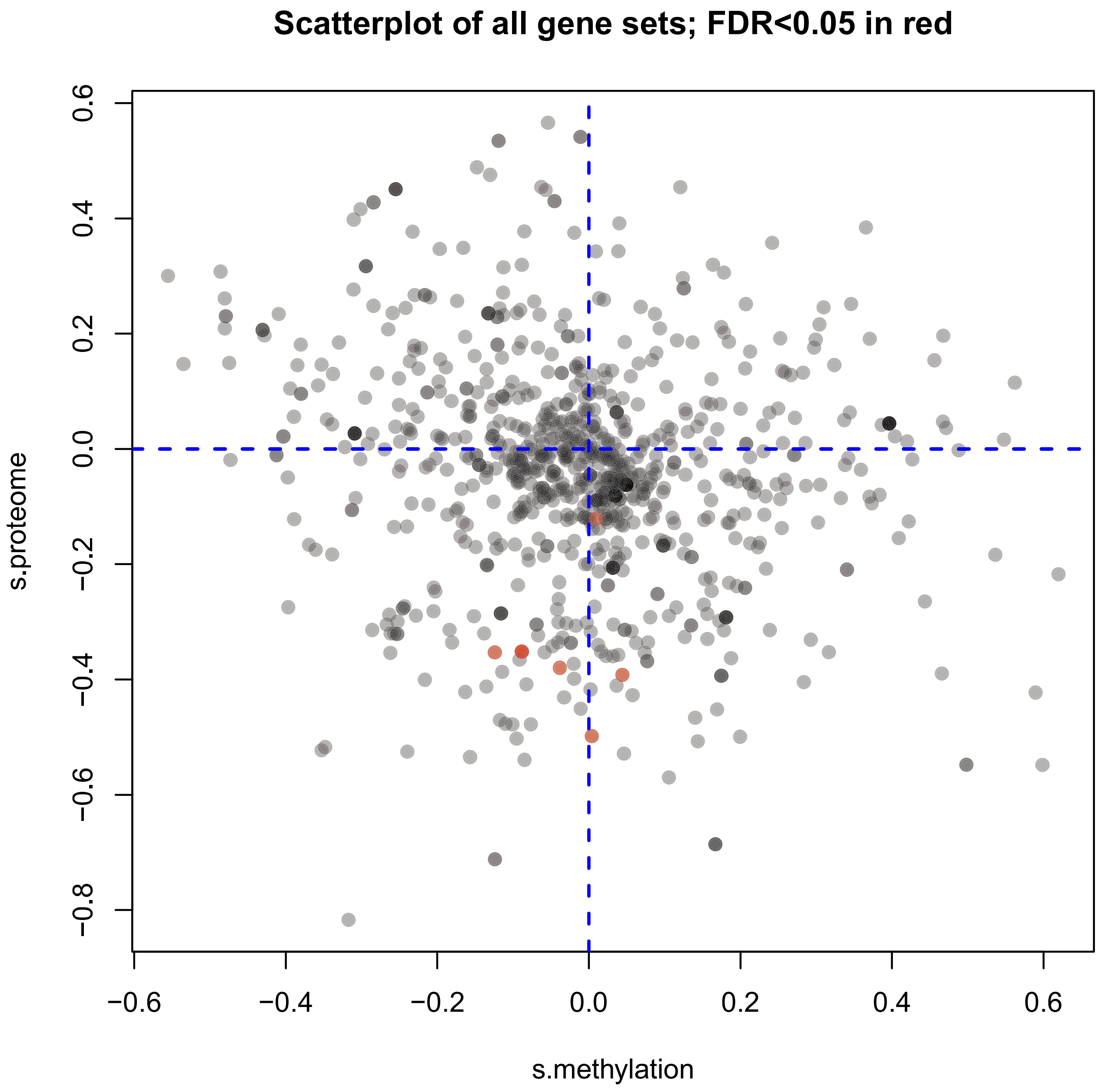

Scatterplot of all gene sets; FDR<0.05 in red
s.proteome
s.methylation
0.6
0.4
0.2
0.0
−0.2
−0.4
−0.6
−0.8
−0.6
−0.4
−0.2
0.0
0.2
0.4
0.6

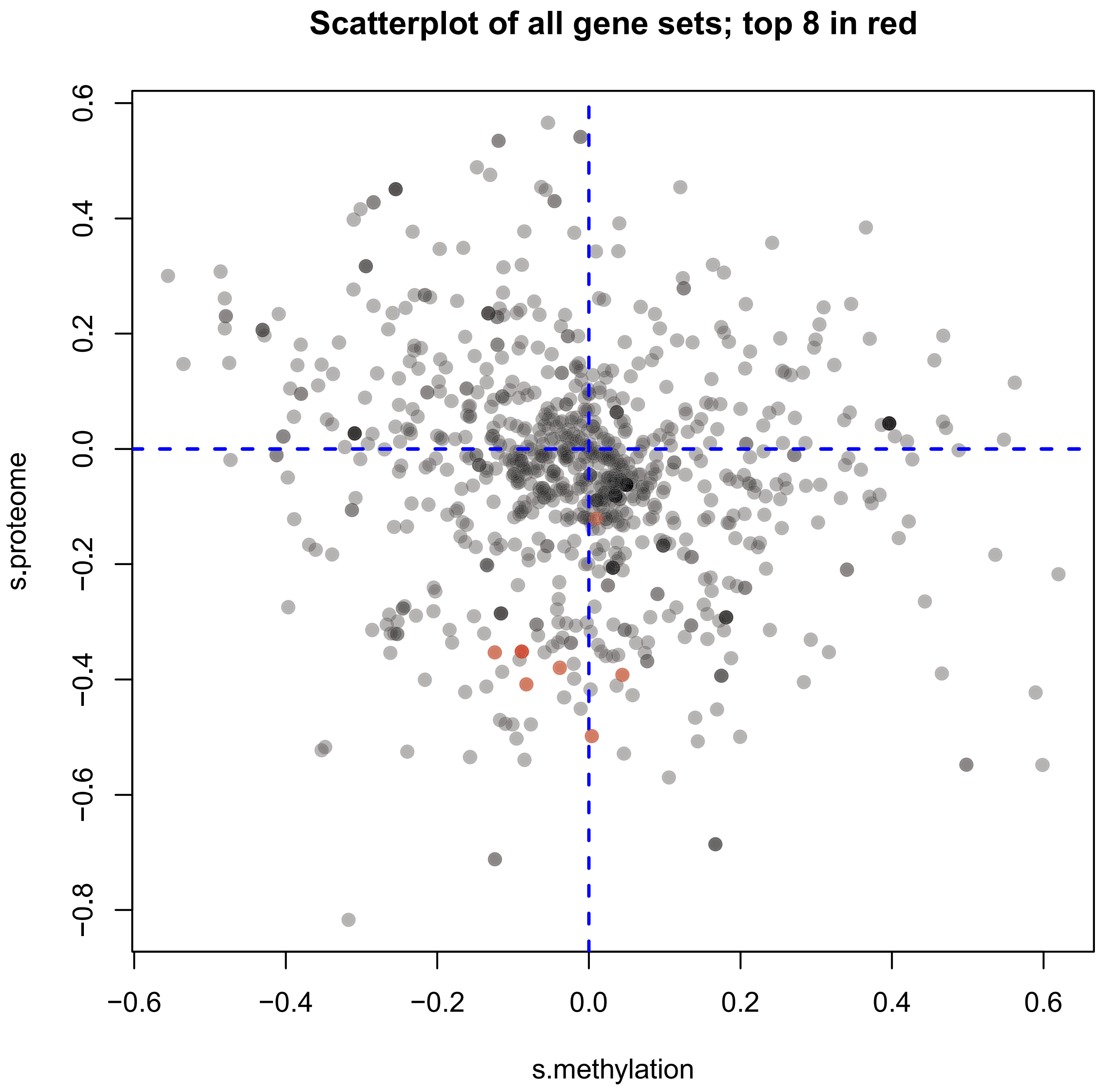
Scatterplot of all gene sets; top 8 in red
s.proteome
s.methylation
0.6
0.4
0.2
0.0
−0.2
−0.4
−0.6
−0.8
−0.6
−0.4
−0.2
0.0
0.2
0.4
0.6

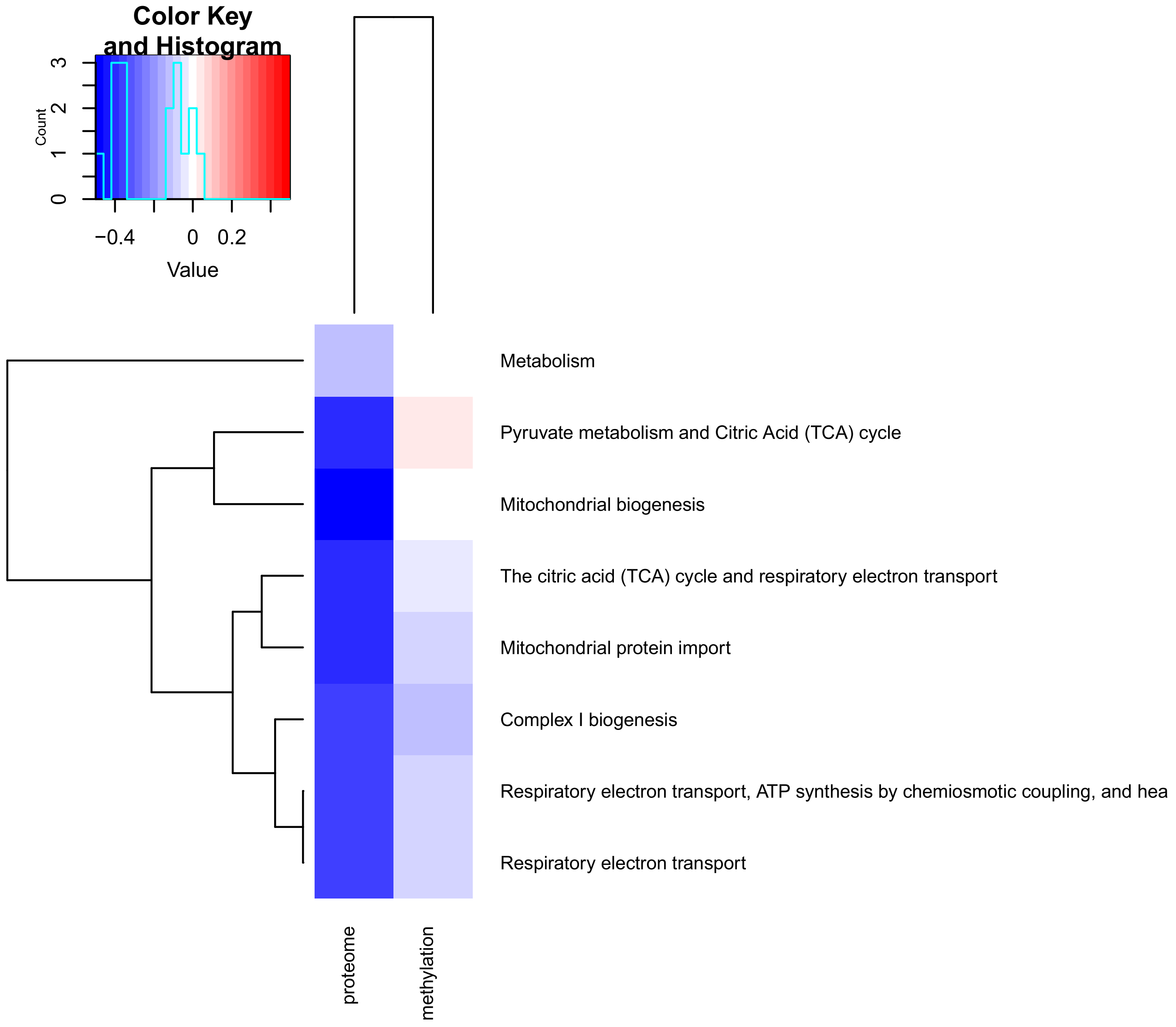

Color Key
and Histogram
Count
0
1
2
3
−0.4
0
0.2
Value
Metabolism
Pyruvate metabolism and Citric Acid (TCA) cycle
Mitochondrial biogenesis
The citric acid (TCA) cycle and respiratory electron transport
Mitochondrial protein import
Complex I biogenesis
Respiratory electron transport, ATP synthesis by chemiosmotic coupling, and hea
Respiratory electron transport
proteome
methylation

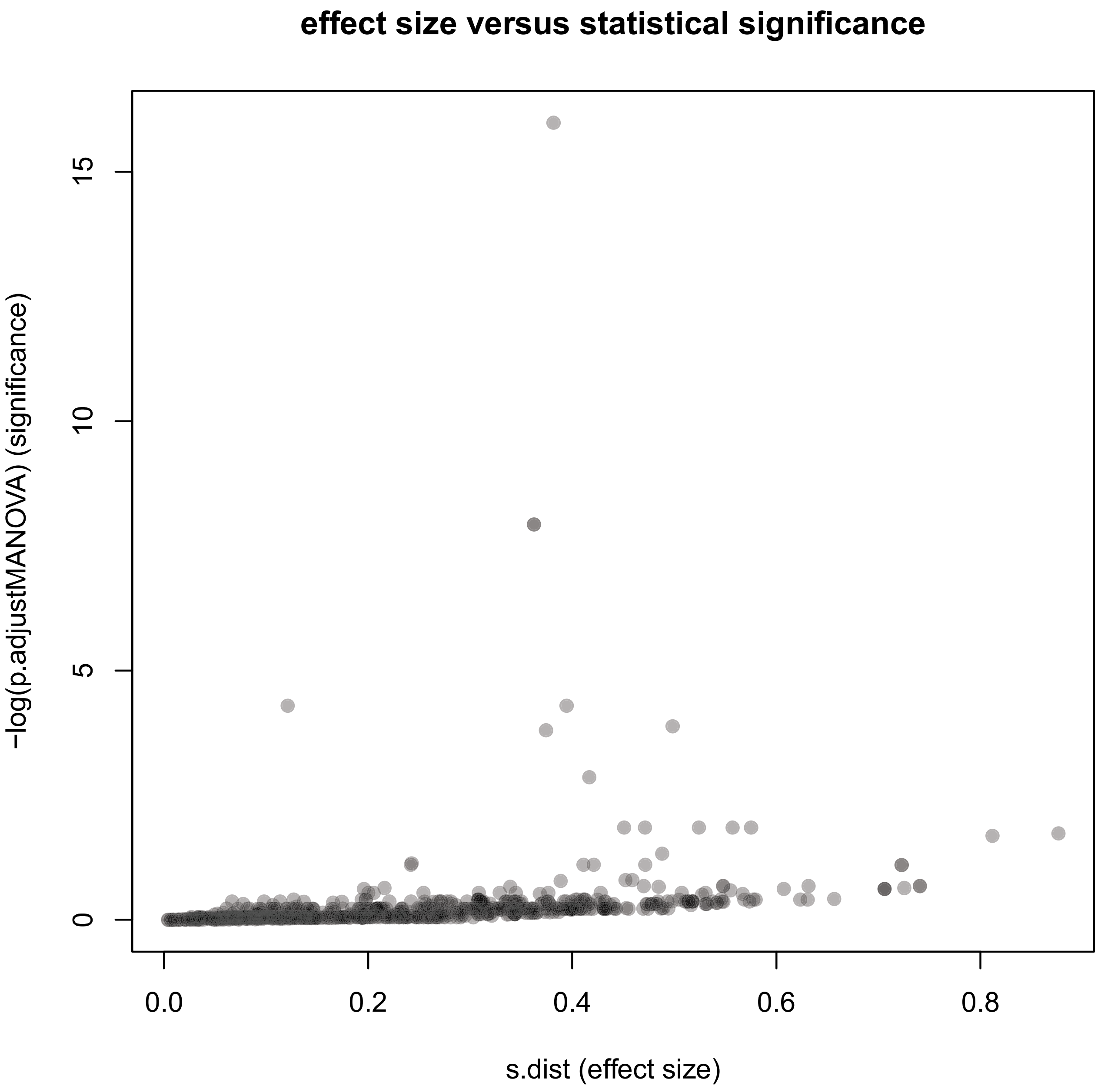
effect size versus statistical significance
−log(p.adjustMANOVA) (significance)
s.dist (effect size)
0
5
10
15
0.0
0.2
0.4
0.6
0.8

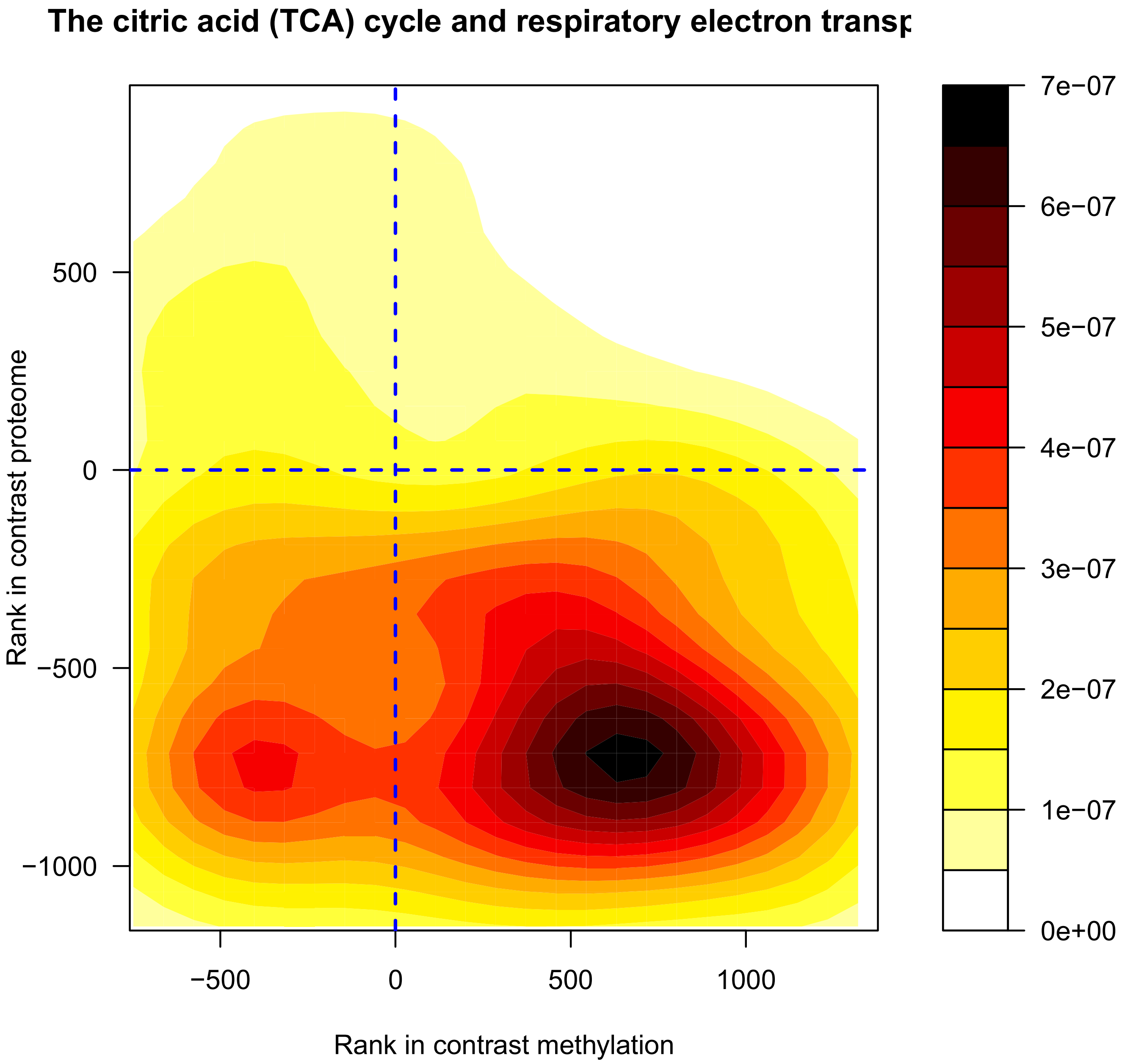

The citric acid (TCA) cycle and respiratory electron transp
Rank in contrast proteome
Rank in contrast methylation
500
0
−500
−1000
−500
0
500
1000
7e−07
6e−07
5e−07
4e−07
3e−07
2e−07
1e−07
0e+00

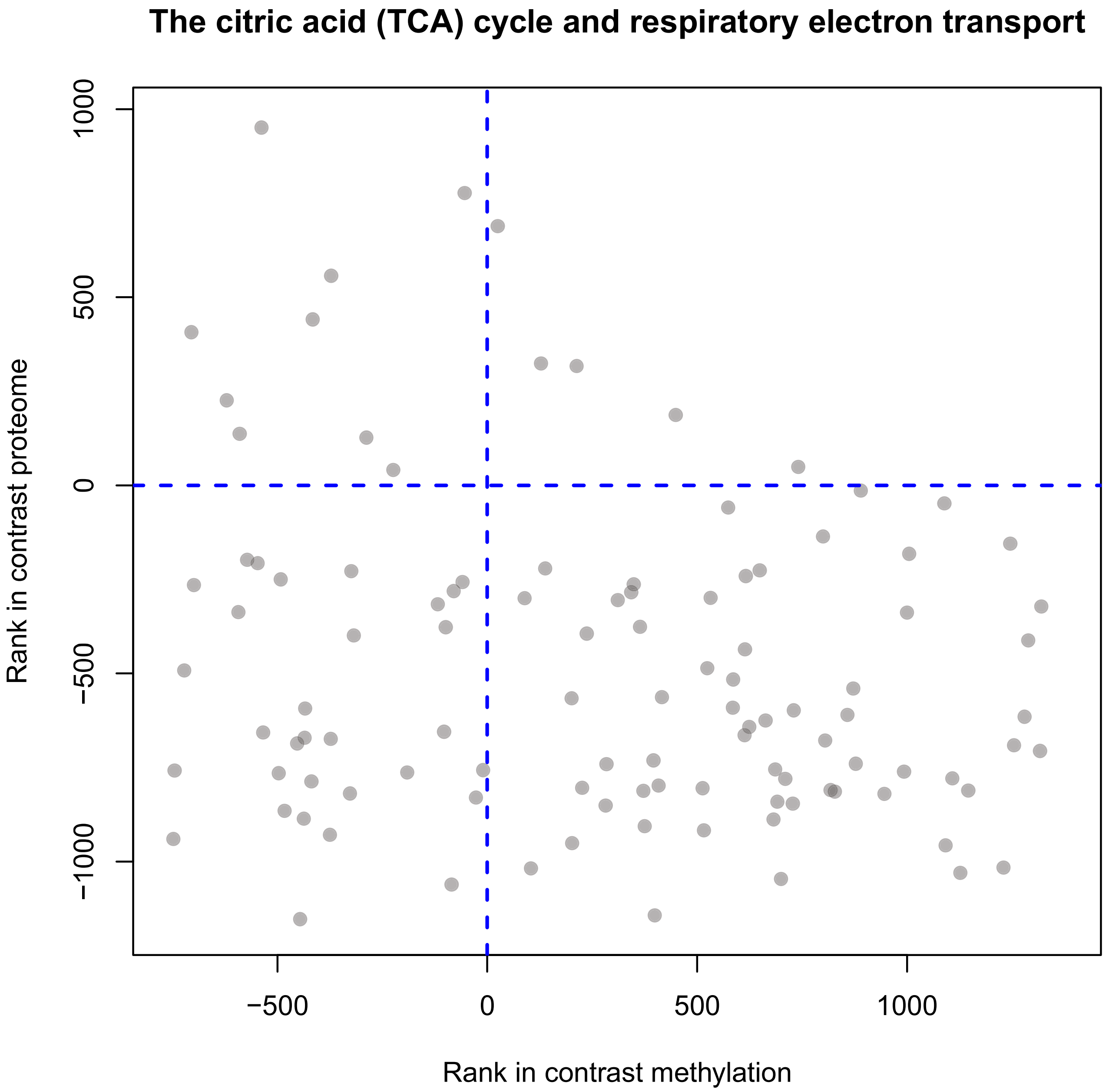
The citric acid (TCA) cycle and respiratory electron transport
Rank in contrast proteome
Rank in contrast methylation
1000
500
0
−500
−1000
−500
0
500
1000

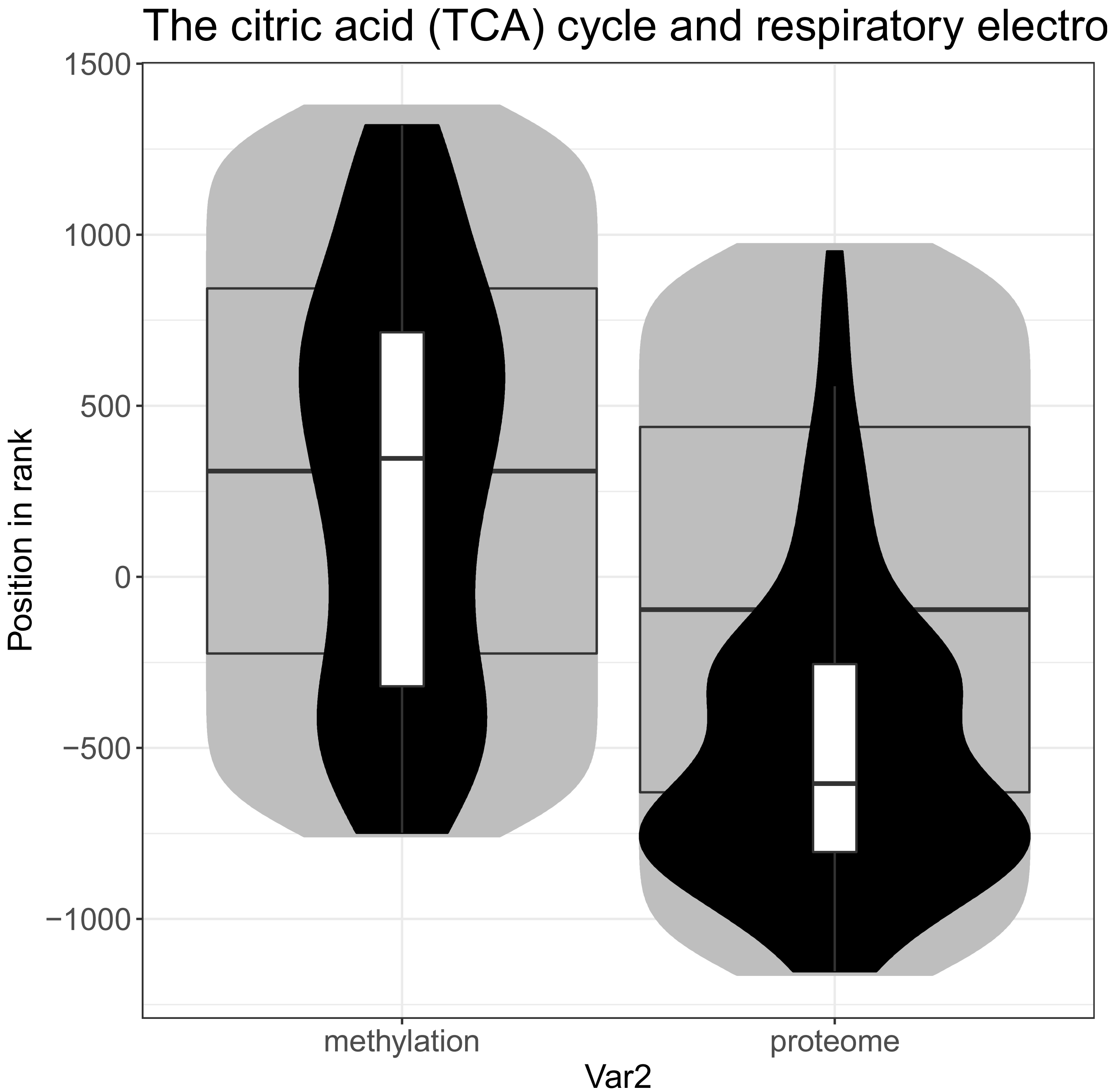

The citric acid (TCA) cycle and respiratory electro
1500
1000
500
0
−500
−1000
Position in rank
methylation
proteome
Var2

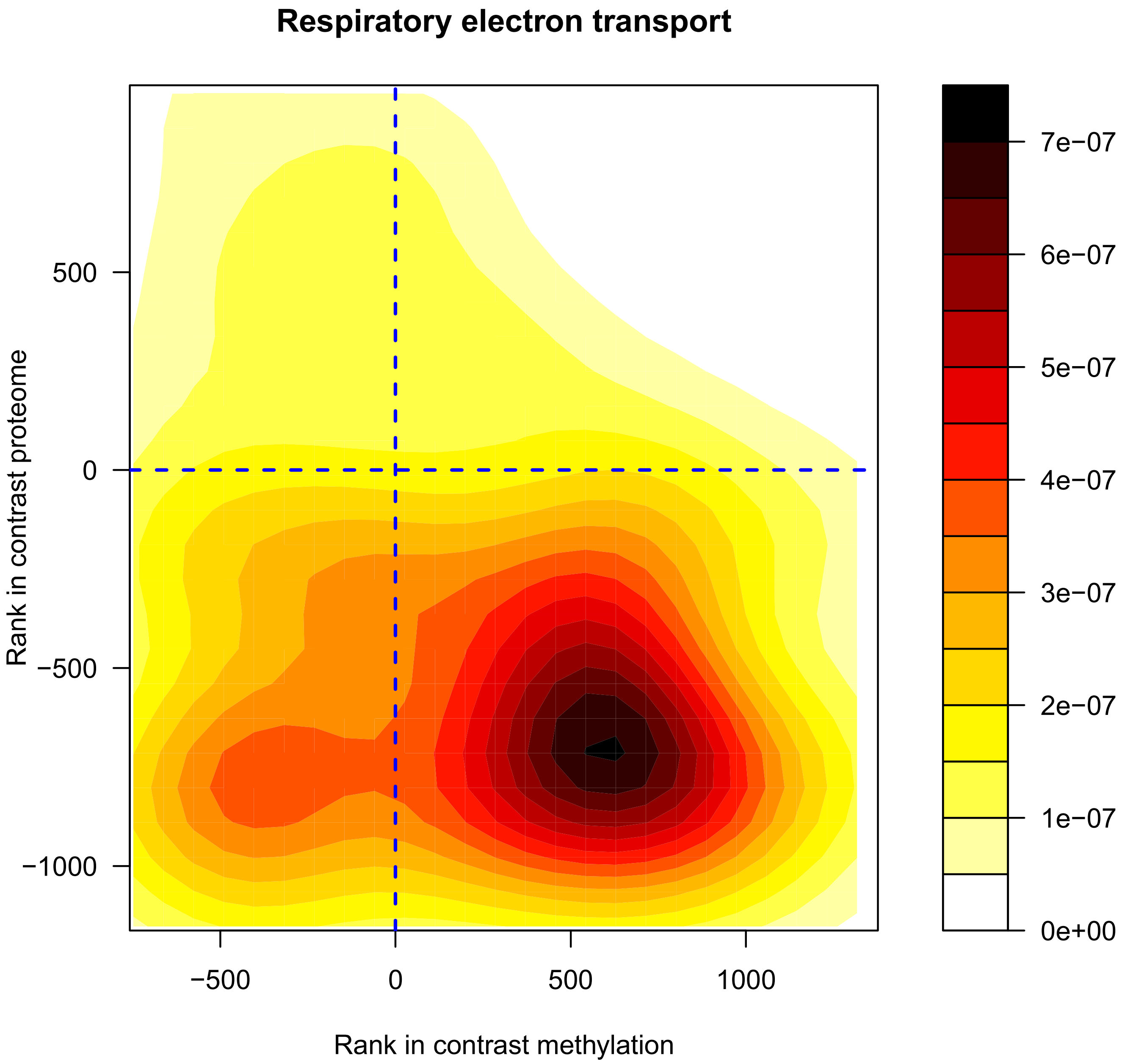

Respiratory electron transport
Rank in contrast proteome
500
0
−500
−1000
−500
0
500
1000
Rank in contrast methylation
7e−07
6e−07
5e−07
4e−07
3e−07
2e−07
1e−07
0e+00

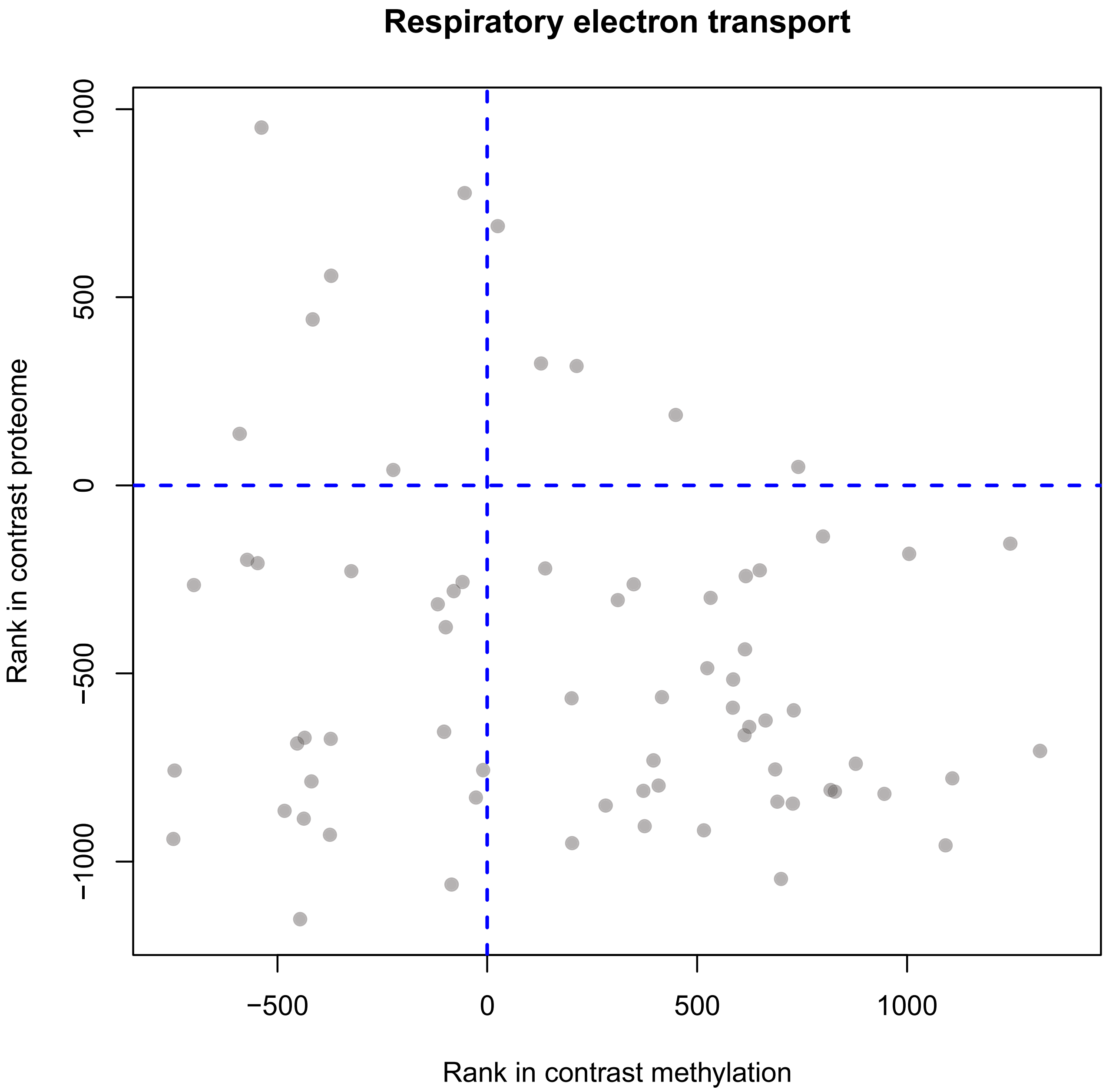
Respiratory electron transport
Rank in contrast proteome
Rank in contrast methylation
1000
500
0
−500
−1000
−500
0
500
1000

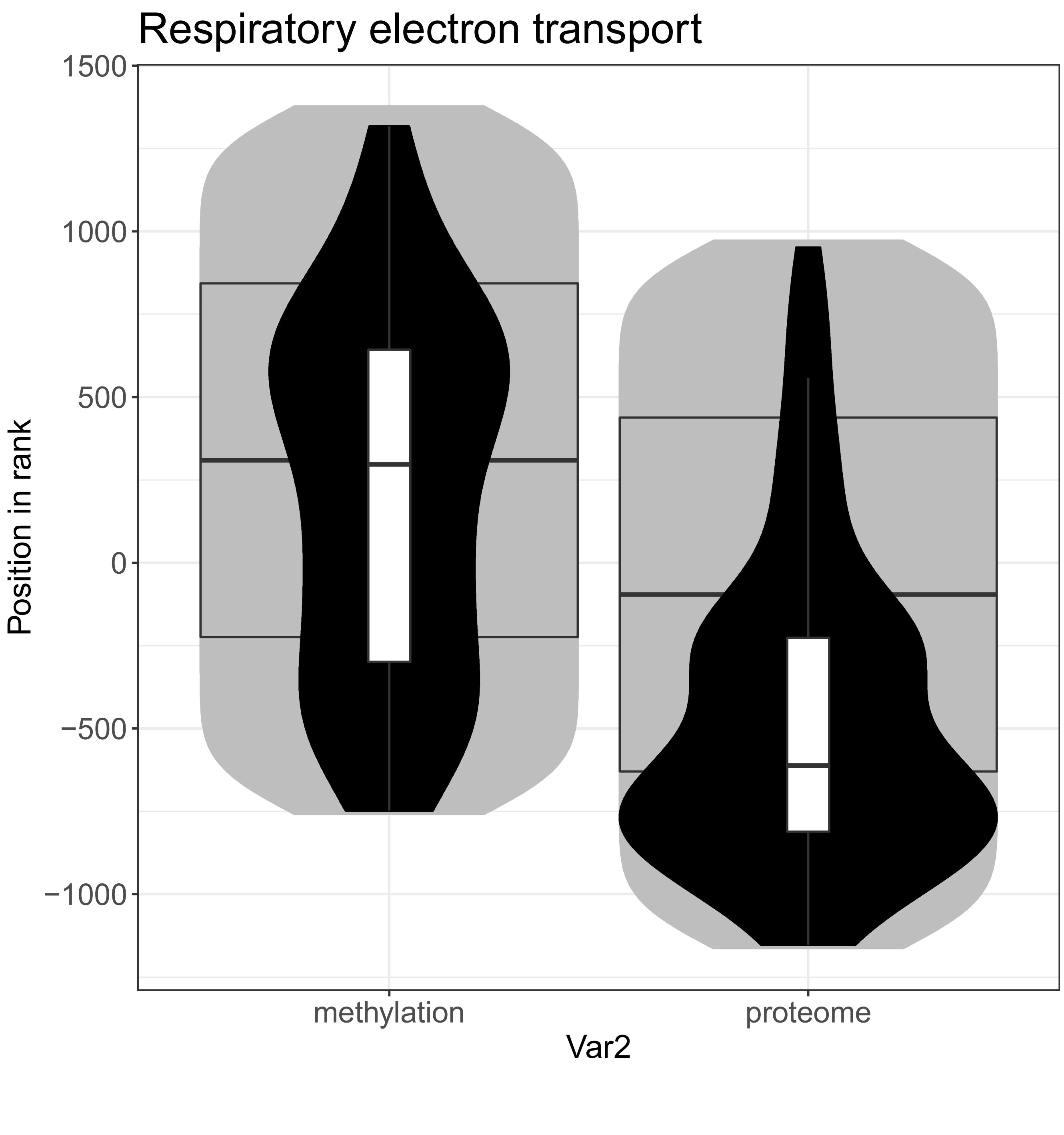
Respiratory electron transport
1500
1000
500
0
−500
−1000
Position in rank
methylation
proteome
Var2
