# Supplementary material for: Sex differences in muscle protein expression and DNA methylation in response to exercise training
Source: Biol Sex Differ. 2023 Sep 5;14:56. doi: 10.1186/s13293-023-00539-2 (PMC10478435; doi:10.1186/s13293-023-00539-2)
Supplement: Supplementary file 4 — Additional file 4: Integration of DNA methylome and proteome for sex*CRF, output from MITCH package. [file 13293_2023_539_MOESM4_ESM.pdf]

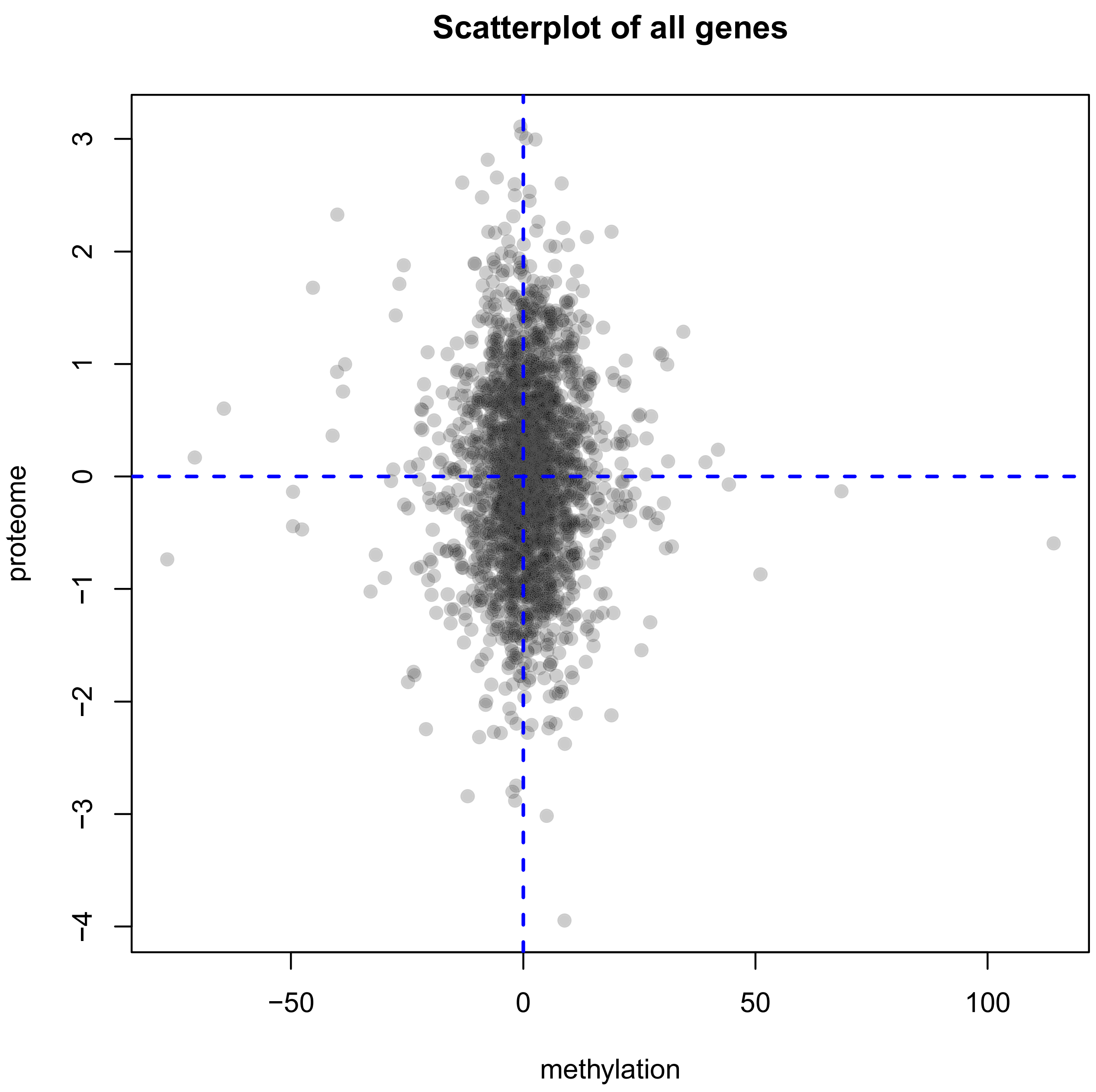
Scatterplot of all genes
proteome
methylation
3
2
1
0
−1
−2
−3
−4
−50
0
50
100

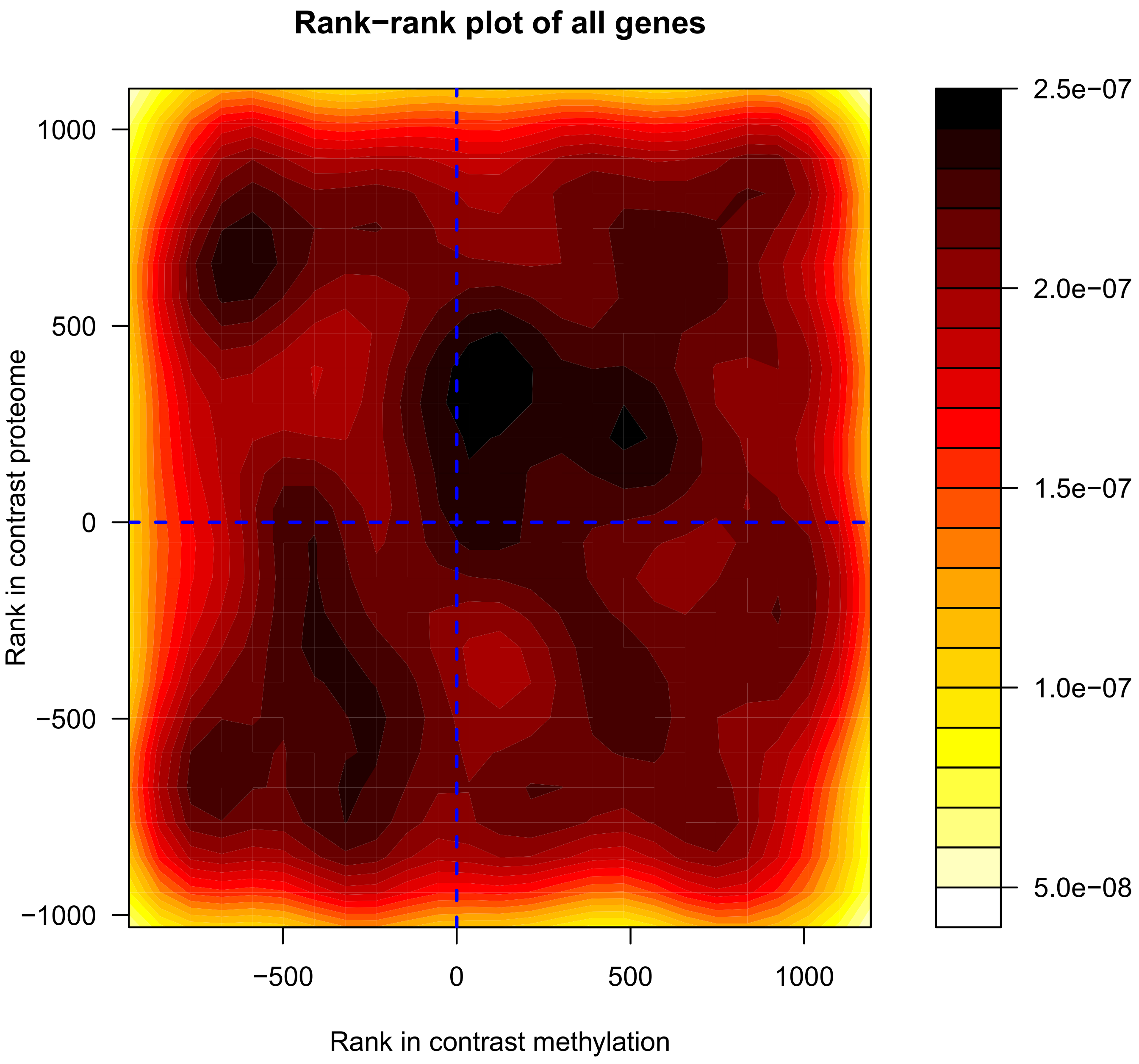

Rank−rank plot of all genes
Rank in contrast proteome
Rank in contrast methylation
1000
500
0
−500
−1000
−500
0
500
1000
2.5e−07
2.0e−07
1.5e−07
1.0e−07
5.0e−08

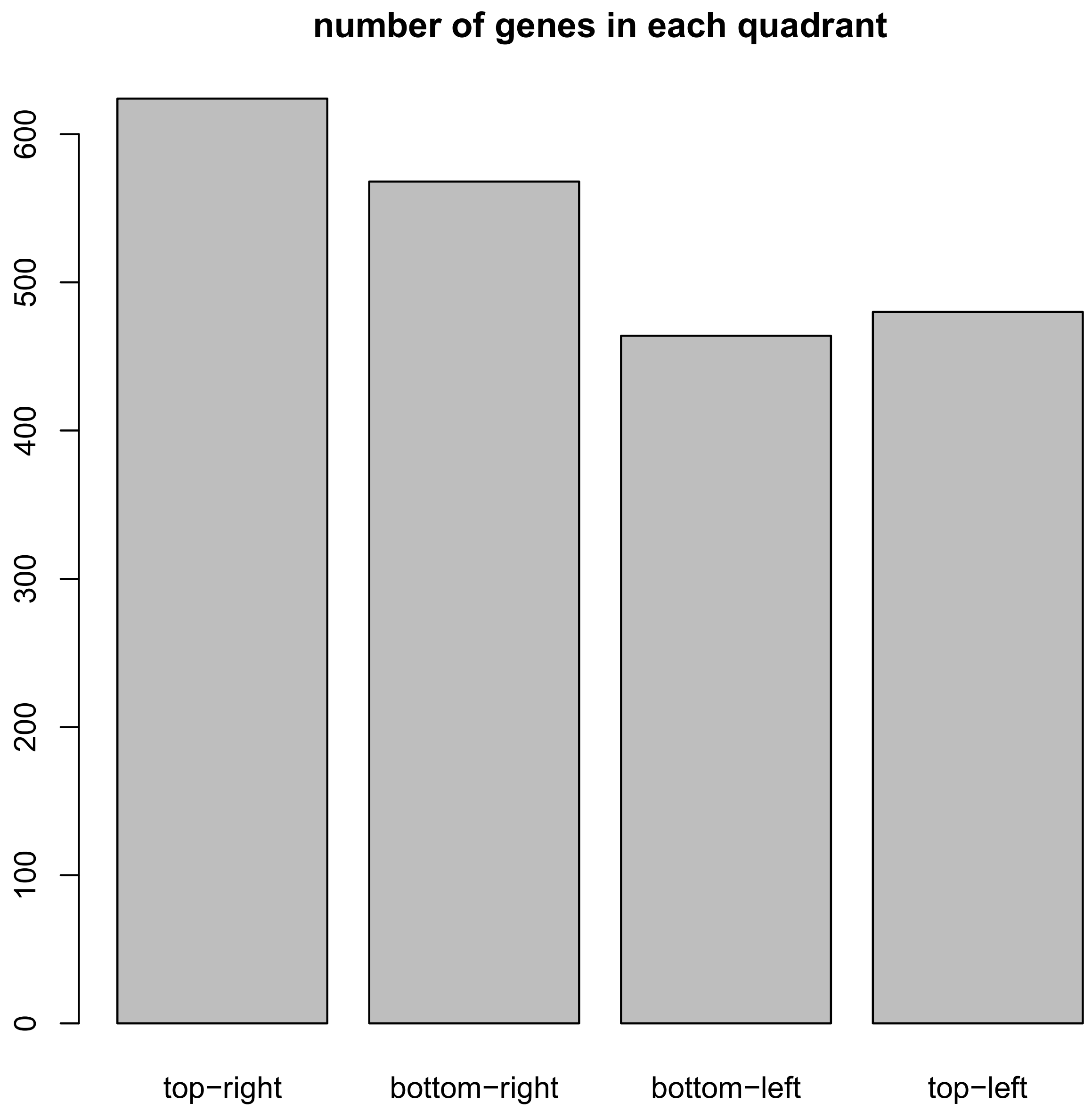
number of genes in each quadrant
0
100
200
300
400
500
600
top−right
bottom−right
bottom−left
top−left

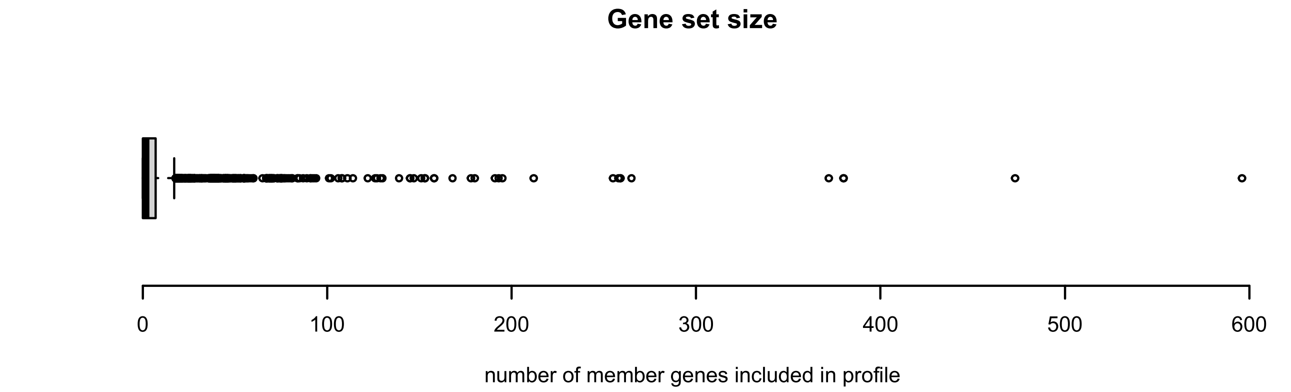
Gene set size
0
100
200
300
400
500
600
number of member genes included in profile

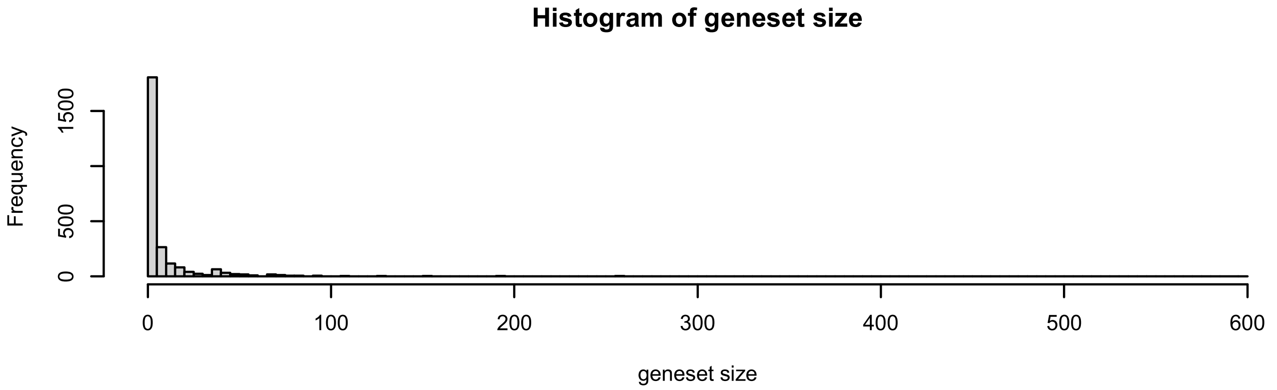
Histogram of geneset size
Frequency
0
500
1500
0
100
200
300
400
500
600
geneset size

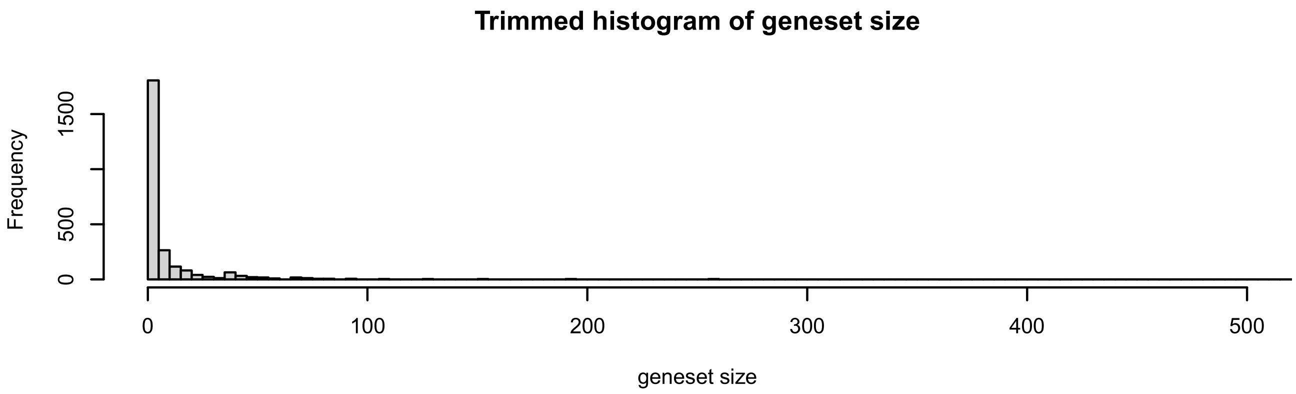
Trimmed histogram of geneset size
Frequency
0
500
1500
0
100
200
300
400
500
geneset size

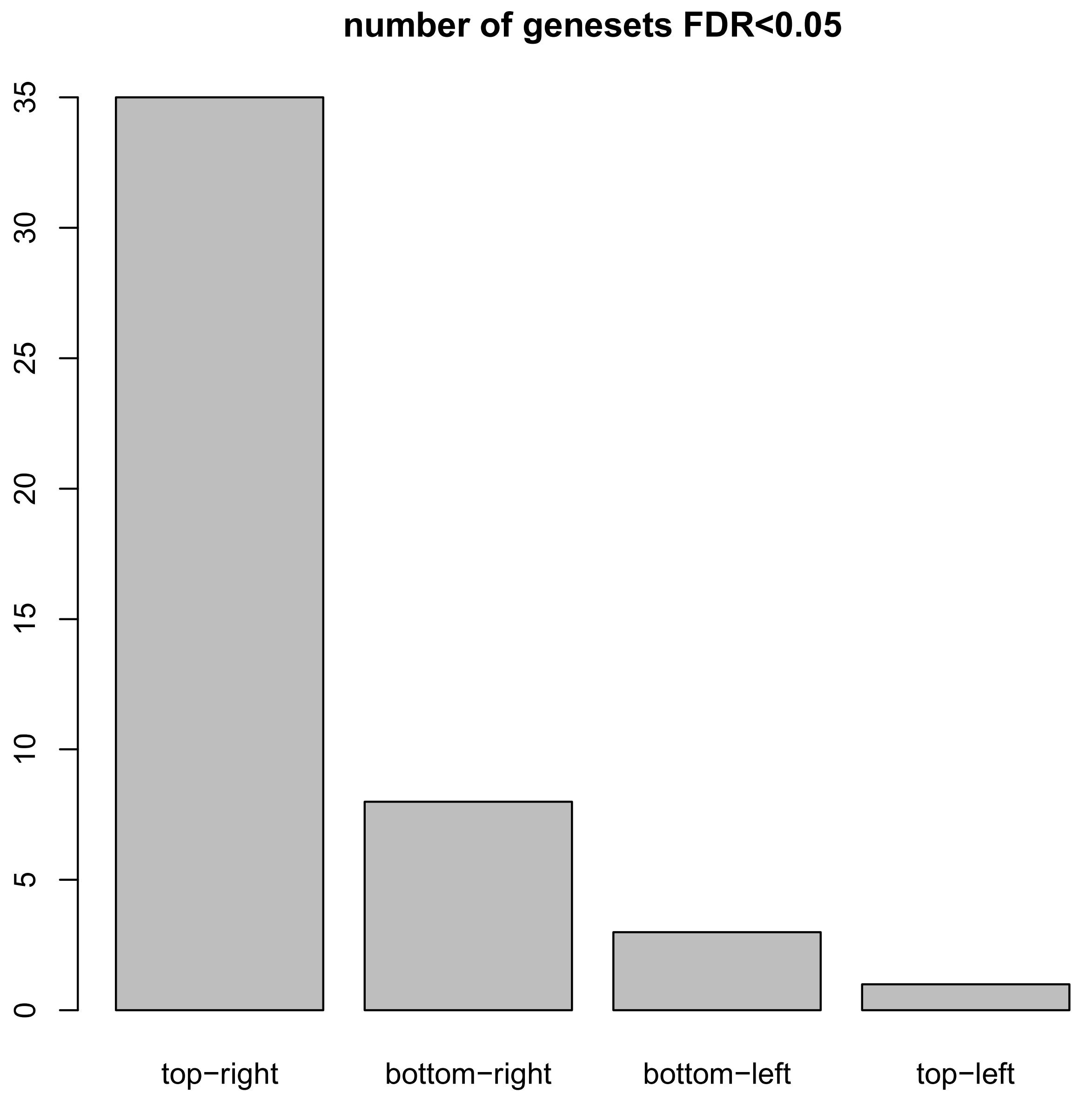

number of genesets FDR<0.05
35
30
25
20
15
10
5
0
top−right
bottom−right
bottom−left
top−left

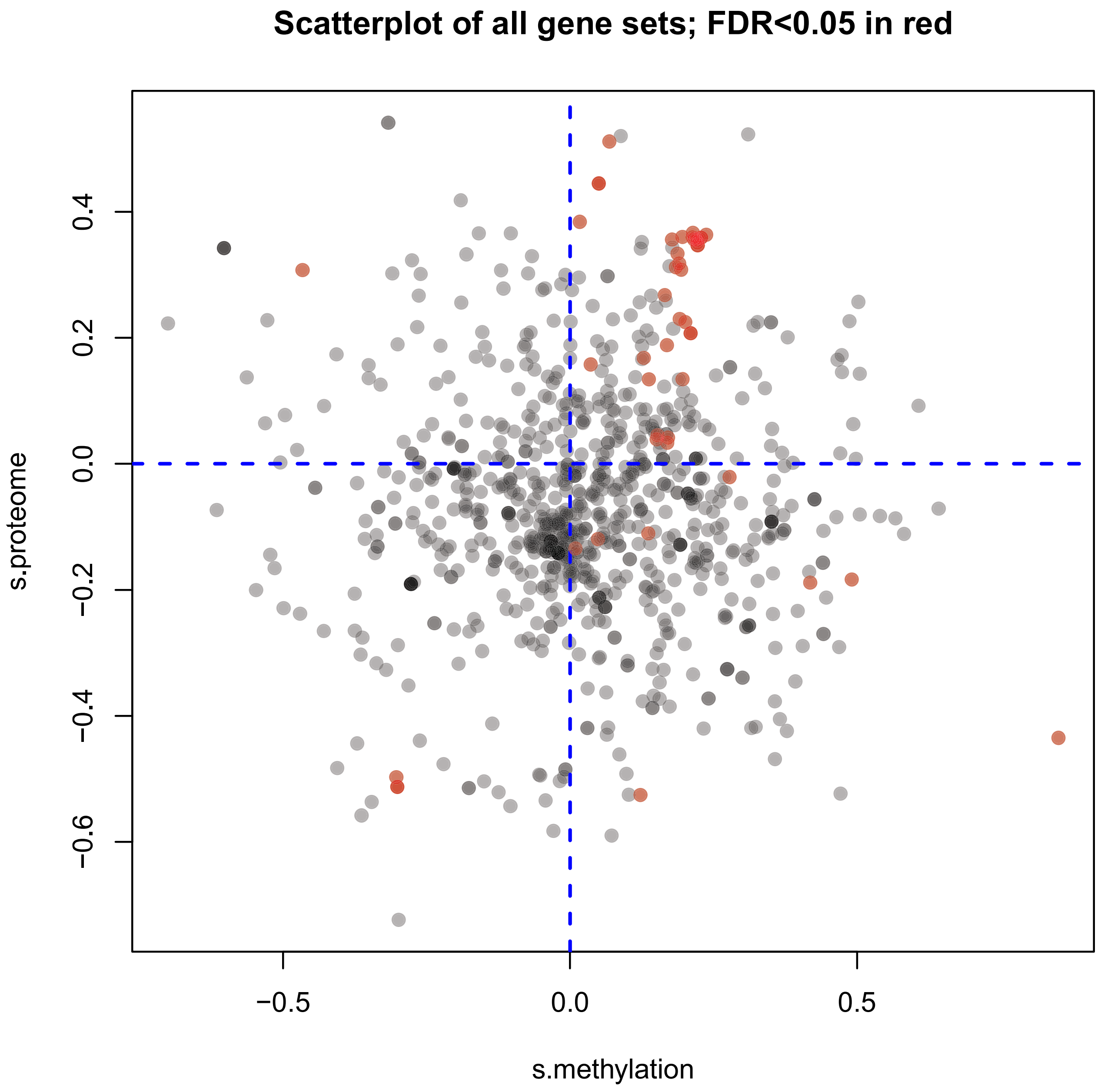

Scatterplot of all gene sets; FDR<0.05 in red
s.proteome
s.methylation
0.4
0.2
0.0
−0.2
−0.4
−0.6
−0.5
0.0
0.5

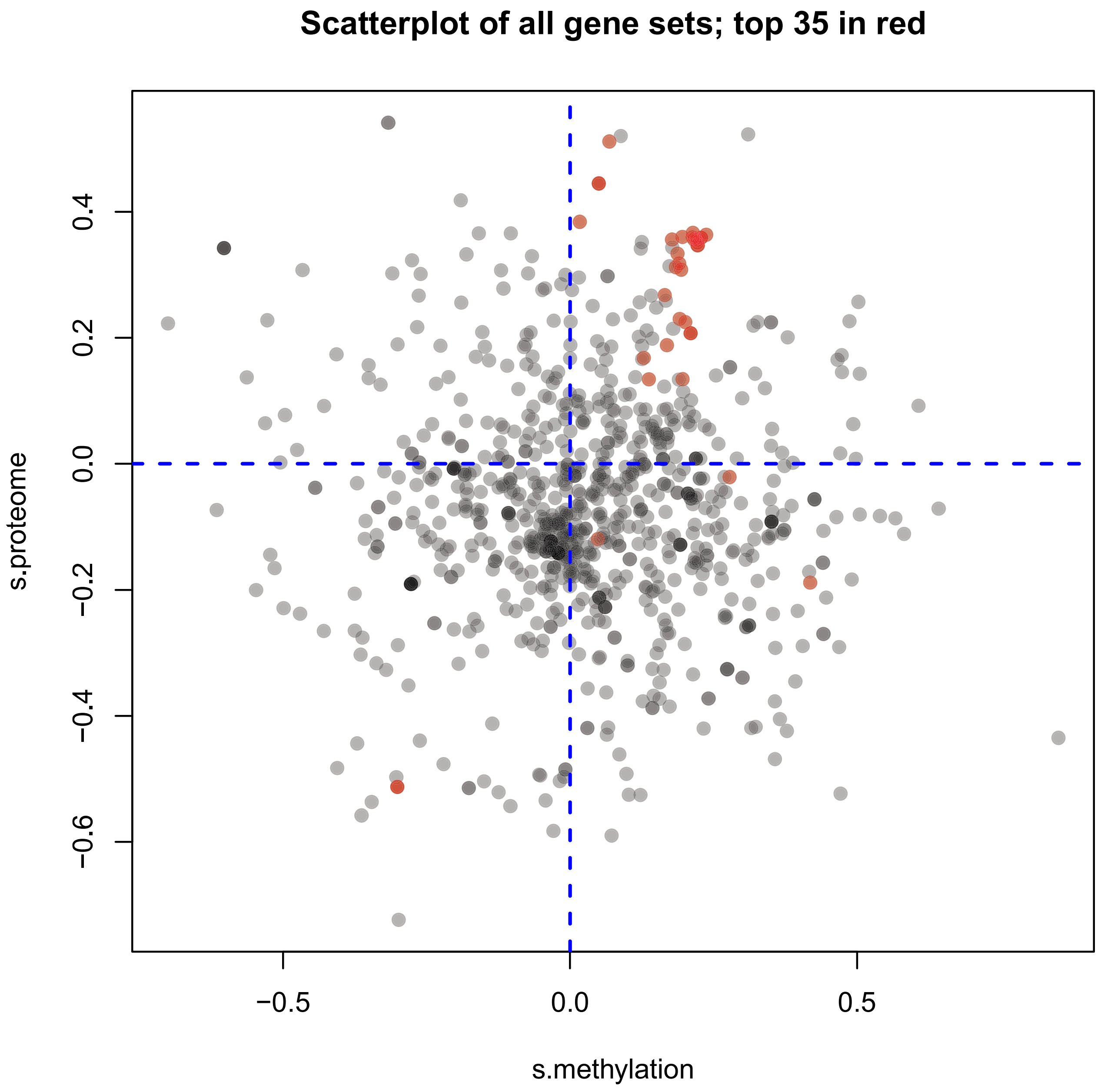

Scatterplot of all gene sets; top 35 in red
s.proteome
s.methylation
0.4
0.2
0.0
−0.2
−0.4
−0.6
−0.5
0.0
0.5

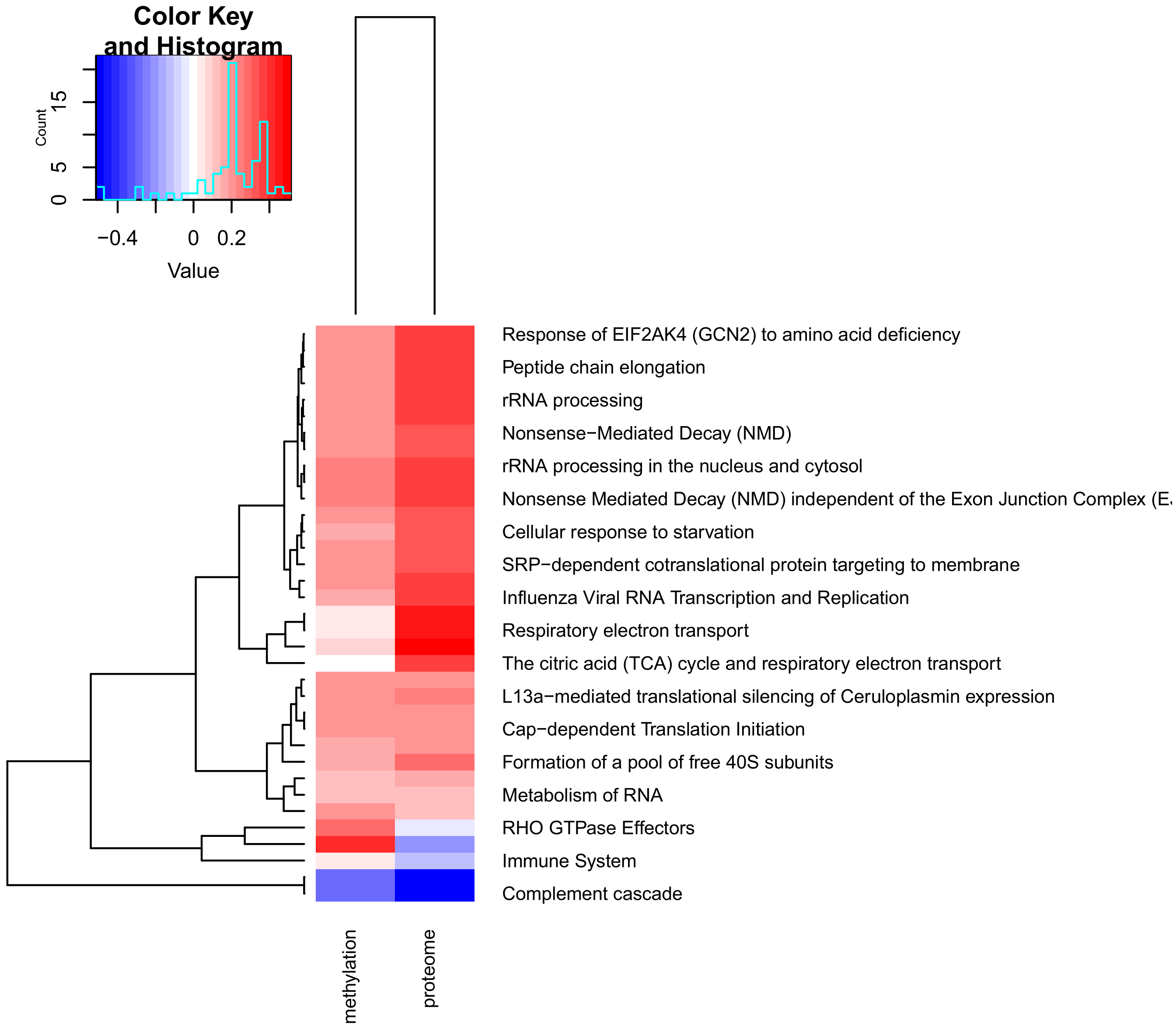
Color Key
and Histogram
Count
0
5
15
−0.4
0
0.2
Value
Response of EIF2AK4 (GCN2) to amino acid deficiency
Peptide chain elongation
rRNA processing
Nonsense−Mediated Decay (NMD)
rRNA processing in the nucleus and cytosol
Nonsense Mediated Decay (NMD) independent of the Exon Junction Complex (E
Cellular response to starvation
SRP−dependent cotranslational protein targeting to membrane
Influenza Viral RNA Transcription and Replication
Respiratory electron transport
The citric acid (TCA) cycle and respiratory electron transport
L13a−mediated translational silencing of Ceruloplasmin expression
Cap−dependent Translation Initiation
Formation of a pool of free 40S subunits
Metabolism of RNA
RHO GTPase Effectors
Immune System
Complement cascade
methylation
proteome

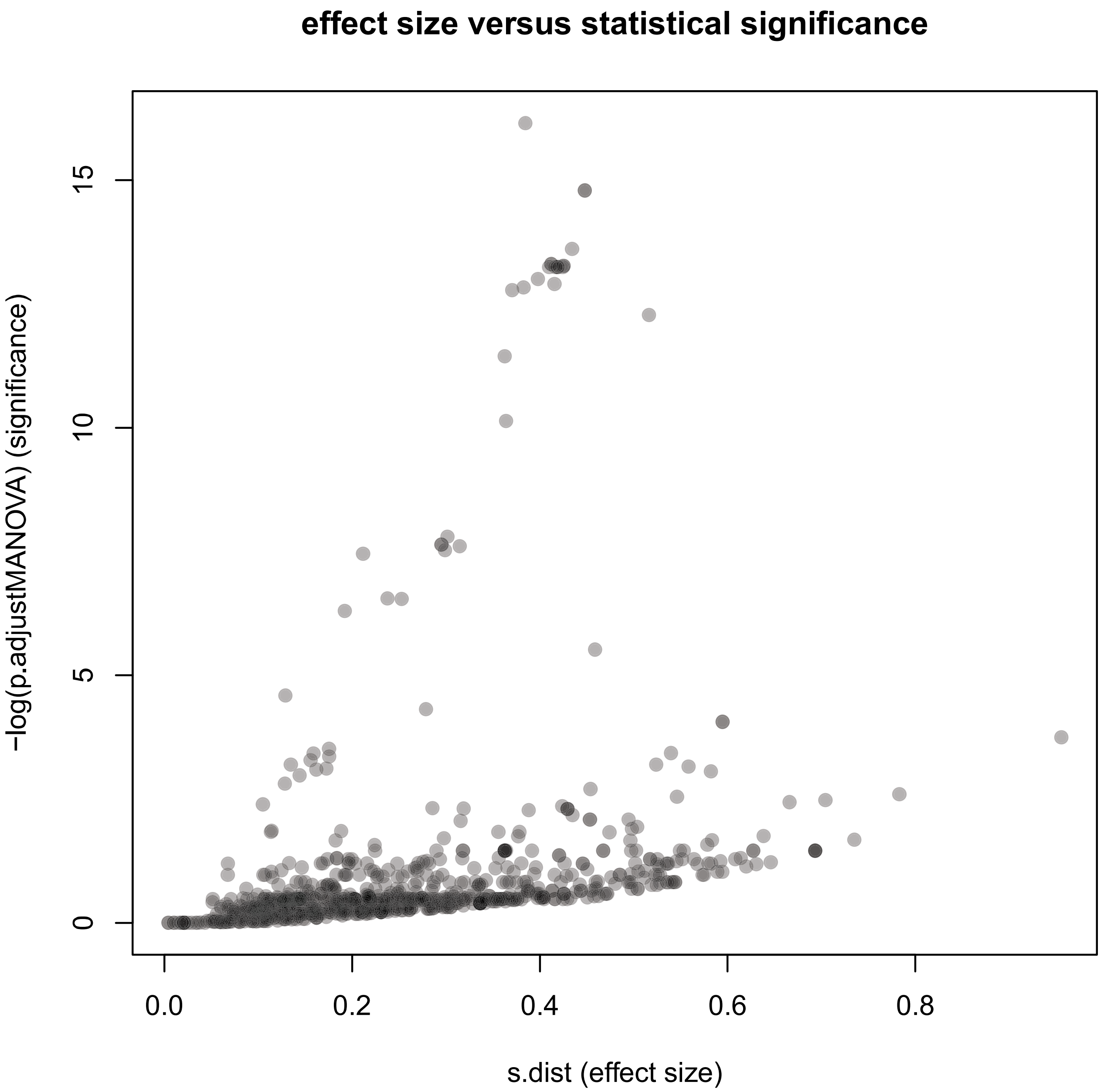
effect size versus statistical significance
−log(p.adjustMANOVA) (significance)
0
5
10
15
0.0
0.2
0.4
0.6
0.8
s.dist (effect size)

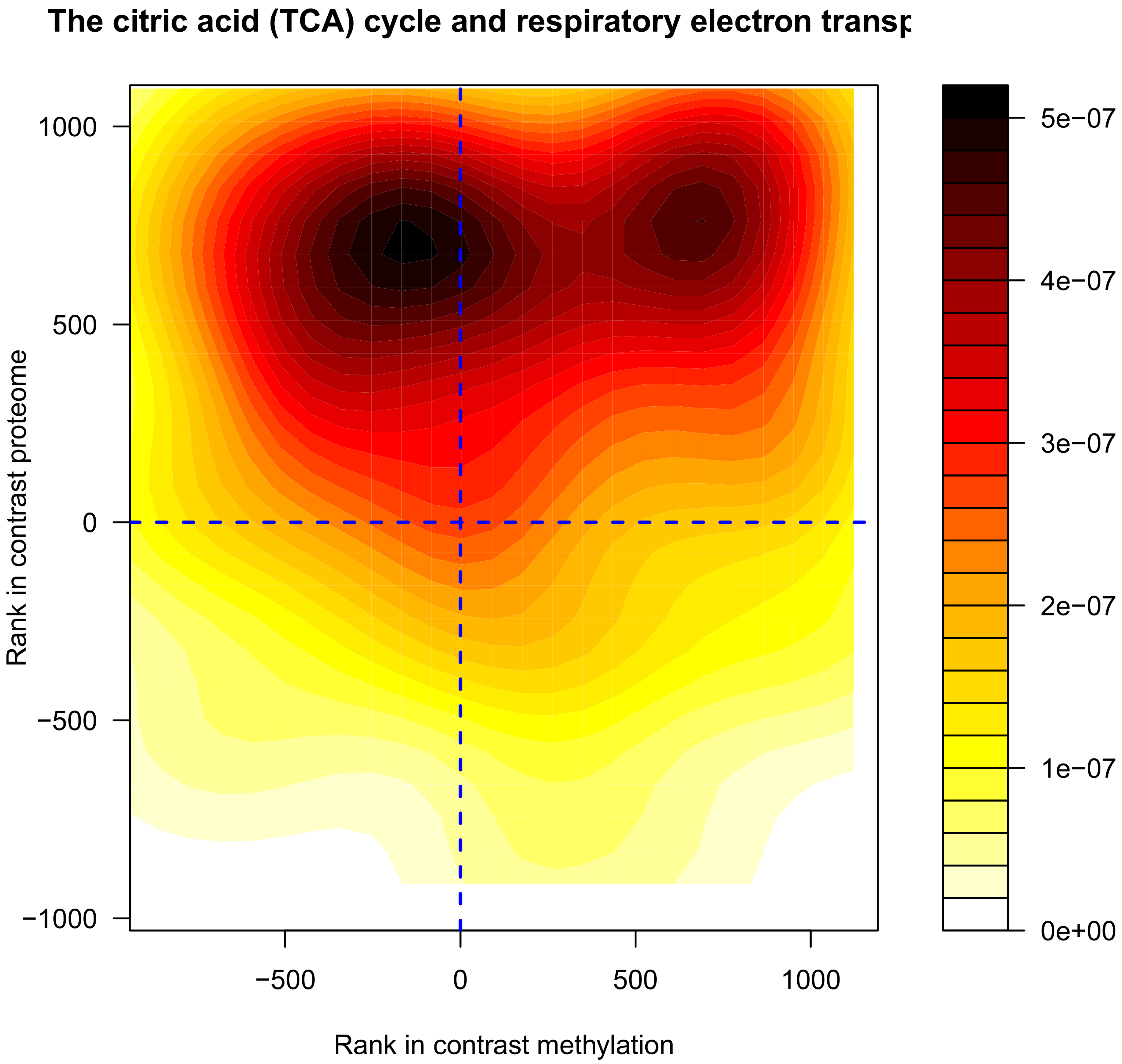
The citric acid (TCA) cycle and respiratory electron transp
Rank in contrast proteome
Rank in contrast methylation
1000
500
0
−500
−1000
−500
0
500
1000
5e−07
4e−07
3e−07
2e−07
1e−07
0e+00

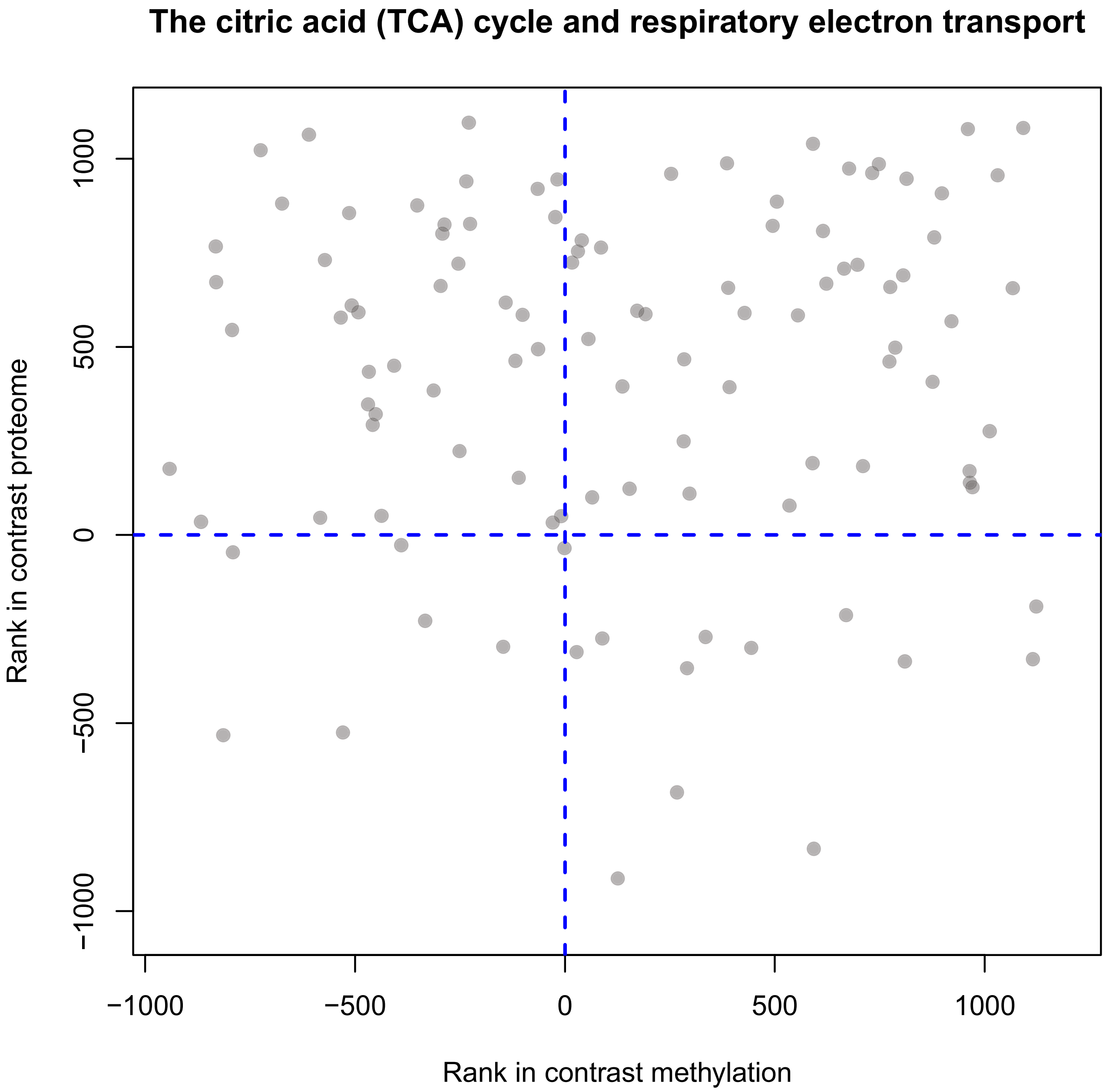

The citric acid (TCA) cycle and respiratory electron transport
Rank in contrast proteome
1000
500
0
−500
−1000
−1000
−500
0
500
1000
Rank in contrast methylation

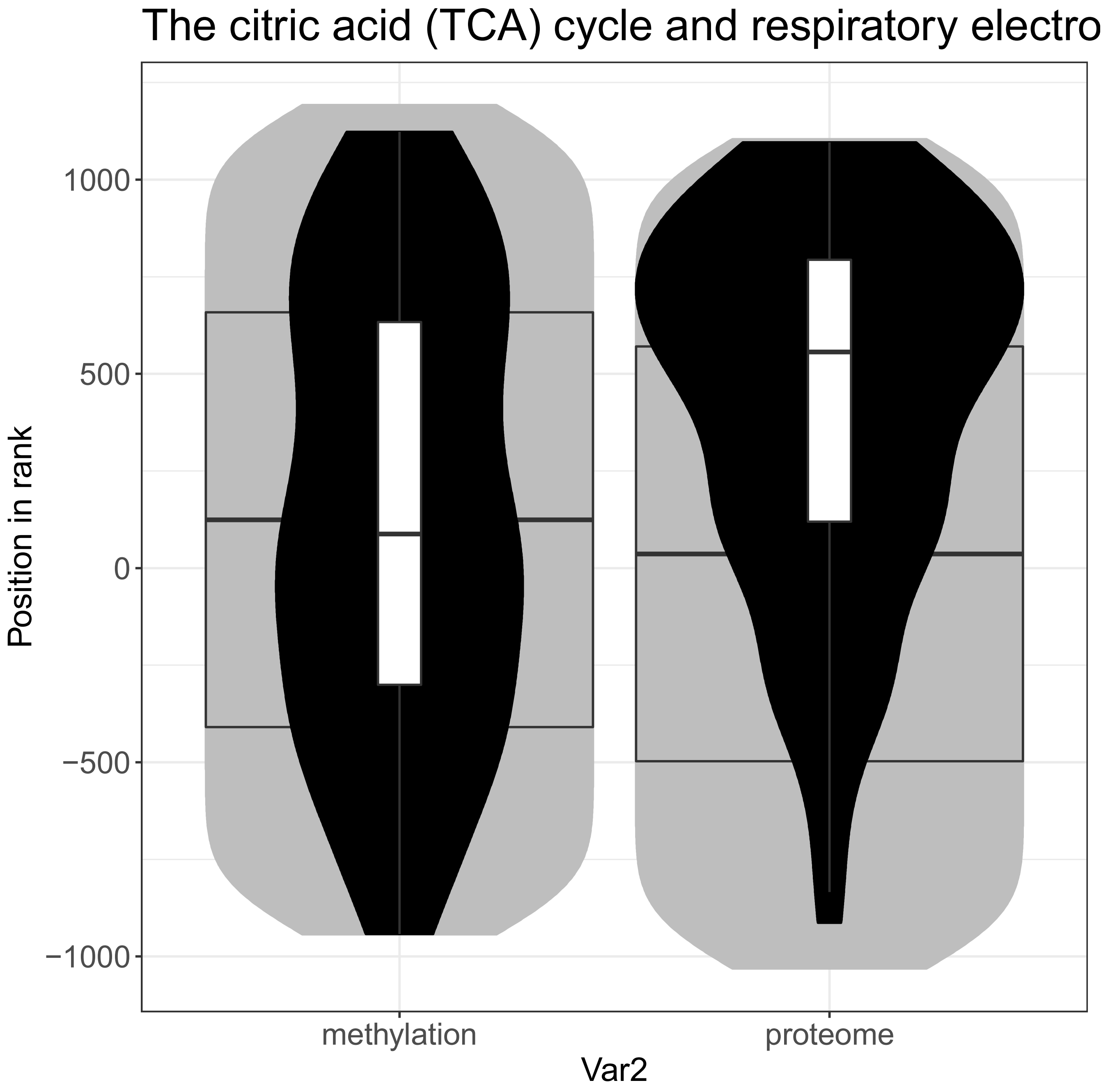

The citric acid (TCA) cycle and respiratory electro
Position in rank
1000
500
0
−500
−1000
methylation
proteome
Var2

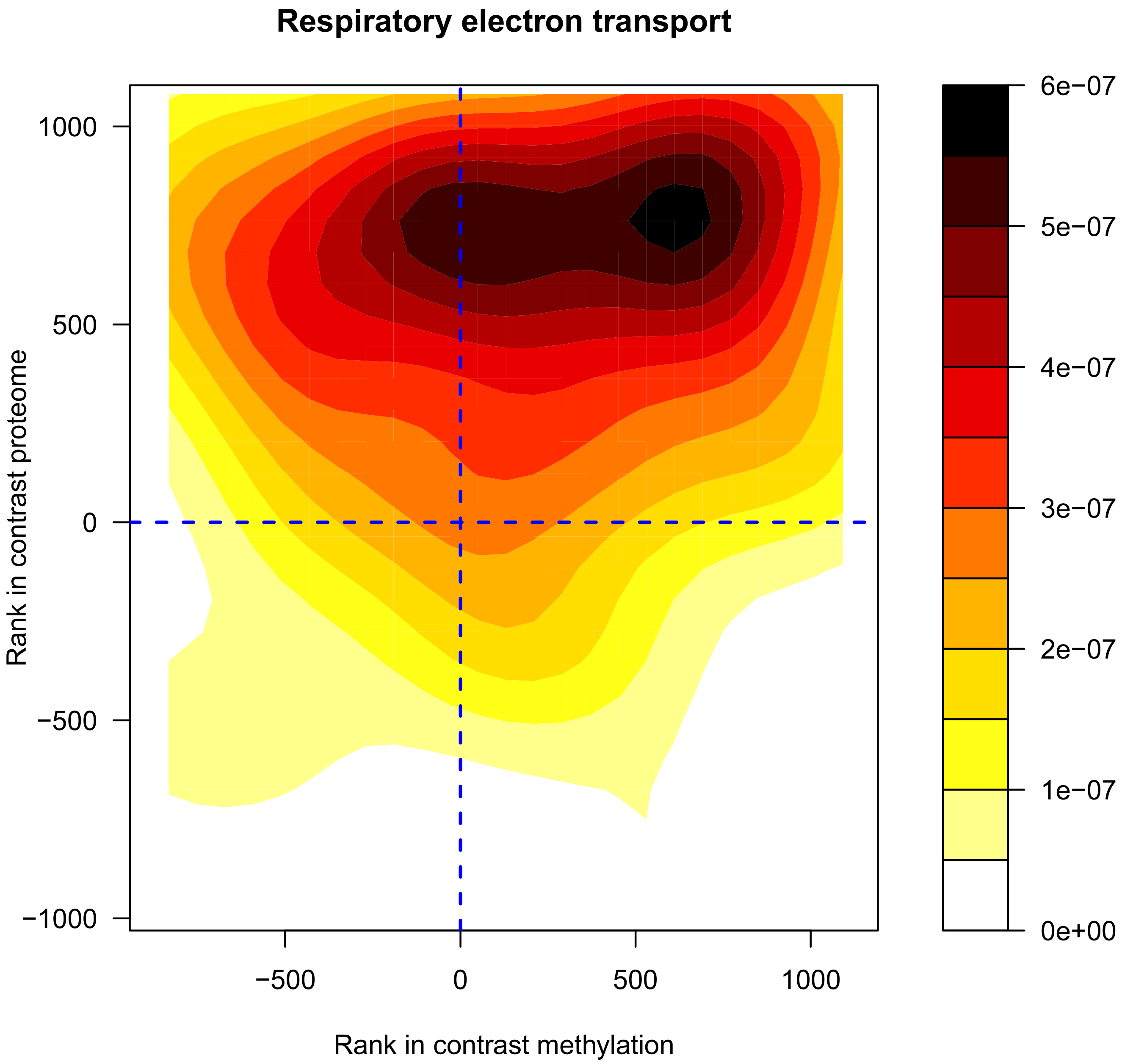

Respiratory electron transport
Rank in contrast proteome
Rank in contrast methylation
1000
500
0
−500
−1000
−500
0
500
1000
6e−07
5e−07
4e−07
3e−07
2e−07
1e−07
0e+00

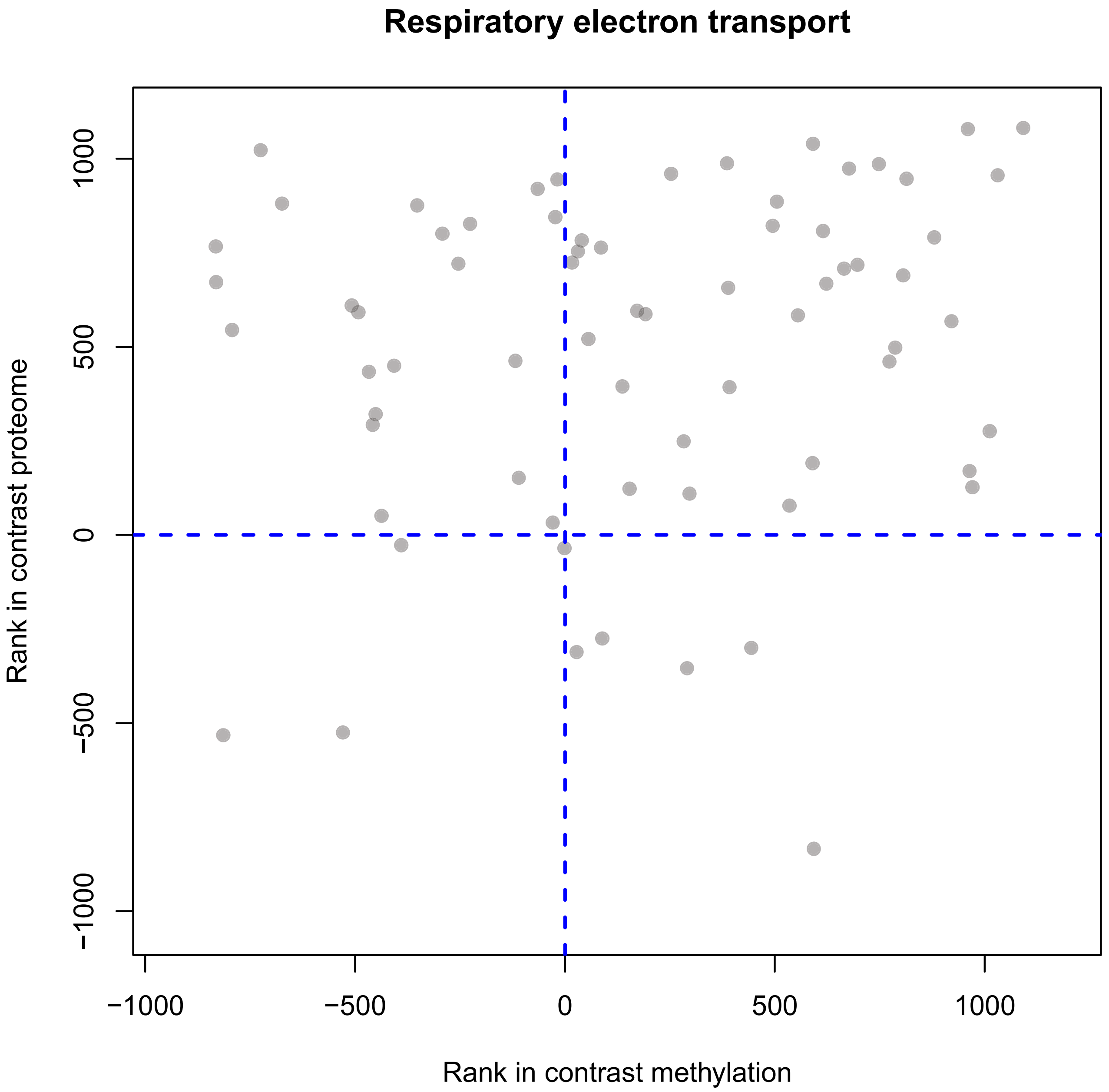
Respiratory electron transport
Rank in contrast proteome
Rank in contrast methylation
−1000
−500
0
500
1000
−1000
−500
0
500
1000

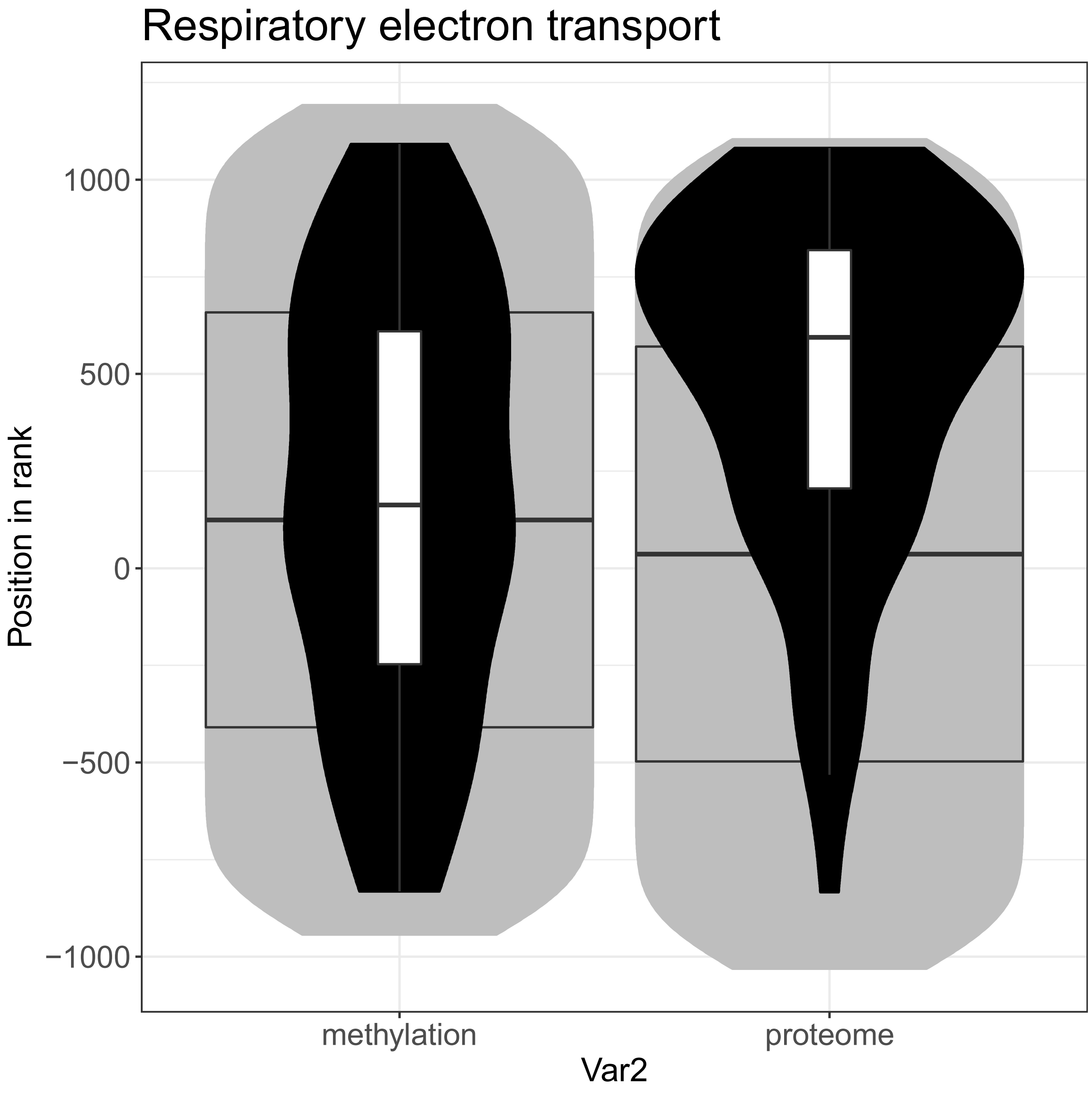

Respiratory electron transport
Position in rank
1000
500
0
−500
−1000
methylation
proteome
Var2
